# Octahedral Zirconium Salan Catalysts for Olefin Polymerization: Substituent and Solvent Effects on Structure and Dynamics

**DOI:** 10.1021/acs.inorgchem.3c02153

**Published:** 2023-09-19

**Authors:** Anna Dall’Anese, Pavel S. Kulyabin, Dmitry V. Uborsky, Antonio Vittoria, Christian Ehm, Roberta Cipullo, Peter H. M. Budzelaar, Alexander Z. Voskoboynikov, Vincenzo Busico, Leonardo Tensi, Alceo Macchioni, Cristiano Zuccaccia

**Affiliations:** † Dipartimento di Chimica, Biologia e Biotecnologie, Università degli Studi di Perugia, Via dell’Elce di sotto 8, 06123 Perugia, Italy; ‡ Department of Chemistry, Lomonosov Moscow State University, 1/3 Leninskie Gory, 119991 Moscow, Russia; § Dipartimento di Scienze Chimiche, Università di Napoli Federico II, Via Cintia, 80126 Napoli, Italy; ∥ DPI, P.O. Box 902, 5600 AX Eindhoven, The Netherlands; ⊥ Dipartimento di Scienze Farmaceutiche, Università degli Studi di Perugia, Via del Liceo 1, 06123 Perugia, Italy

## Abstract

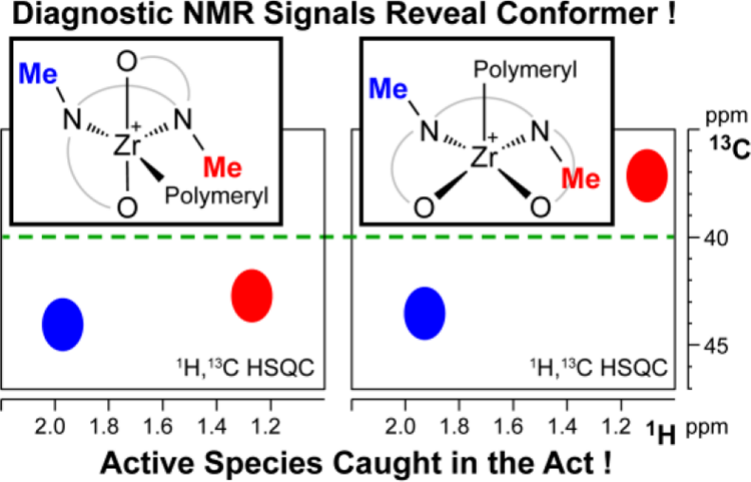

Group 4 metal-Salan olefin polymerization catalysts typically have relatively low activity, being slowed down by a pre-equilibrium favoring a non-polymerization active resting state identified as a *mer-mer* isomer (MM); formation of the polymerization active *fac-fac* species (FF) requires isomerization. We now show that the chemistry is more subtle than previously realized. Salan variations bearing large, flat substituents can achieve very high activity, and we ascribe this to the stabilization of the FF isomer, which becomes *lower* in energy than MM. Detailed in situ NMR studies of a fast (*o*-anthracenyl) and a slow (*o*-^*t*^Bu) Salan precursors, suitably activated, indicate that preferred isomers in solution are different: the fast catalyst prefers FF while the slow catalyst prefers a highly distorted MM geometry. Crystal structures of the *activated o*-anthracenyl substituted complex with a moderately (chlorobenzene) and, more importantly, a weakly coordinating solvent (toluene) in the first coordination sphere emphasize that the active FF isomer is preferred, at least for the benzyl species. Site epimerization (SE) barriers for the fast catalyst (Δ*S* > 0, dissociative) and the slow catalyst (Δ*S* < 0, associative) in toluene corroborate the solvent role. Diagnostic NMe ^13^C chemical shift differences allow unambiguous detection of FF or MM geometries for seven activated catalysts in different solvents, highlighting the role of solvent coordination strength and bulkiness of the *ortho*-substituent on the isomer equilibrium. For the first time, active *polymeryl* species of Zr-Salan catalysts were speciated. The slow catalyst is effectively trapped in the inactive MM state, as previously suggested. Direct observation of fast catalysts is hampered by their high reactivity, but the product of the first 1-hexene insertion maintains its FF geometry.

## Abstract 2

Cationic group 4 Salan catalysts for olefin polymerization may speciate into various possible geometrical isomers, some of which may be inactive. Rationalizing how the nature of ligand and solvent modulates the equilibria between isomers, and then catalytic performance, is challenging. Detailed NMR/DFT analysis of cationic precursors allowed to individuate diagnostic, easily accessible, NMR parameters capable of discriminating between active and inactive isomers under catalytic conditions.

## Introduction

In homogeneous-phase olefin polymerization catalysis, the vast majority of catalytic systems are highly electrophilic cationic complexes of group 4 metals.^1−4^ Since catalytic reactions are conducted in low-polarity solvents, ion pairs are considered the true active species, and the strength of the cation-anion interaction is known to strongly affect catalytic performance.^5,6^

Typical metallocene and *ansa*-metallocene systems have the great advantage that the active species retain the pseudo-tetrahedral (rigid) structure of the precursors.^7,8^ Although synthetically challenging, the ligand(s) can be rationally engineered, based on conceptually simple molecular descriptors, to optimize catalytic performance.^9^ Moreover, it is also possible to analyze in detail how the interactions between the cation, anion, and solvent modulate the structure, dynamics, and reactivity of the active species.^10^ For example, using both systems of industrial interest and models derived from them, it has been possible, through nuclear magnetic resonance (NMR) studies, to investigate the specific effect of the solvent on the epimerization processes of the active site,^11−14^ as well as the relationship between the thermodynamic tendency of ion pairs to form higher-order ion aggregates^15^ and the kinetic effect of the latter on the single olefin insertion reaction in the metal–carbon bond.^16^

Most group 4 post-metallocene catalysts are inherently more flexible and the corresponding activated species often speciate into various possible geometrical isomers, some of which may be inactive.^17^ On the other hand, many post-metallocenes feature an easier precatalyst synthesis and superior performance at high temperatures.^18^ Rationalizing how the equilibria between isomers or conformers^19^ modulate the overall catalytic performance is difficult. In some cases, the ligand framework may additionally even undergo in situ modification by reaction with monomer(s)^20−23^ or other additives (e.g., main group metal alkyls).^24^

**Figure 1:**
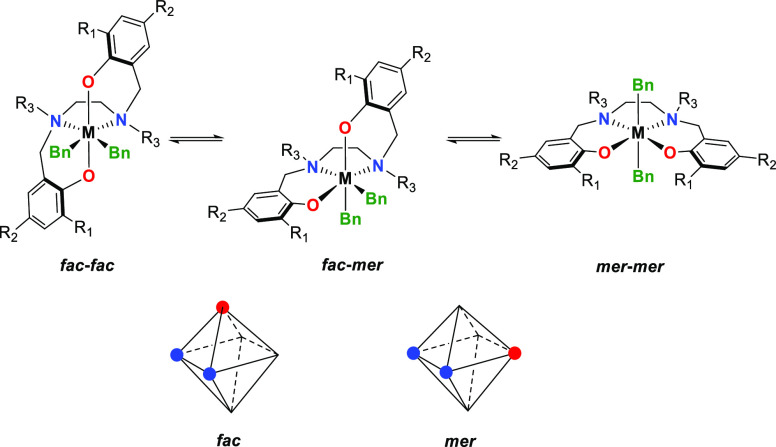
Illustrations of the *fac* and *mer* isomerism for octahedral [ONNO] Salan-type catalysts (top) and the possible geometries of [ONN] fragments giving rise to the *fac* and *mer* nomenclature (bottom).

Bis(phenoxy-amine) complexes (“Salan-type”) of group 4 metals represent a typical example in this respect (Figure 1). First introduced by Kol and co-workers,^25^ these octahedral systems have been of particular interest as molecular catalysts resembling, in some respect, the octahedral catalytic site of heterogeneous Ziegler-Natta (ZN) Ti catalysts.^26^

Facile Salan synthesis by a one-pot Mannich condensation between amines, formaldehyde, and substituted phenols allows easy structural amplification, especially in comparison to the often-complex metallocene synthesis. Most studies on Salan complexes focused on tuning polymerization performance by ligand design; a wide range of polypropylene properties can be accessed through variations of R_1_ and R_2_ substituents (Figure 1).^27,28^ The tuning space with respect to activity is remarkable: introduced originally as isospecific, slow and *living* polymerization catalysts (R_1_ = ^*t*^Bu), polymerization activity increases by five orders of magnitude with R_1_ = anthracenyl.^29^ Studies on the structural analysis of these catalysts in solution are rare and greatly complicated by an inherently flexible ligand framework. The [ONNO] ligand can wrap around the metal in several ways, leading to multiple energetically accessible isomers (*fac*-*fac* (FF), *fac*-*mer* (FM), and *mer*-*mer* (MM), after the *fac* or *mer* geometrical arrangement of the two [ONN] fragments, see Figure 1).^30,31^

The *C*_2_-symmetric FF isomer is generally favored in neutral precatalysts LMR_2_ (R = Bn,^25,28,32^ Me_2_CHO,^27,33^ M = group 4 metal), but solution NMR spectroscopy and density functional theory (DFT) concluded that the MM isomer is the most stable isomer for pentacoordinated active cations, adopting a distorted square pyramidal geometry, independently of the nature of R^1^ and R^2^.^31^ Even with a “sticky counteranion” like [MeB(C_6_F_5_)_3_]^−^, an outer sphere ion pair (OSIP) is formed.^30^ As the coordination vacancy and growing polymeryl chain are in a *trans* arrangement, the MM isomer is polymerization inactive. For insertion to occur, the complex must rearrange to the active FF isomer, likely passing through an FM isomer (Figure 1). If the FF isomer is higher in energy than the MM isomer, then the MM ⇆ FM pre-equilibrium is of course an integral part of the effective propagation barrier, and DFT studies have shown that the energy difference between the MM and FF isomers correlates well with activity as long as the anion is included computationally.^29,34^ Non-specific solvent effects were included via the conductor-like screening model (COSMO).

**Scheme 1:**
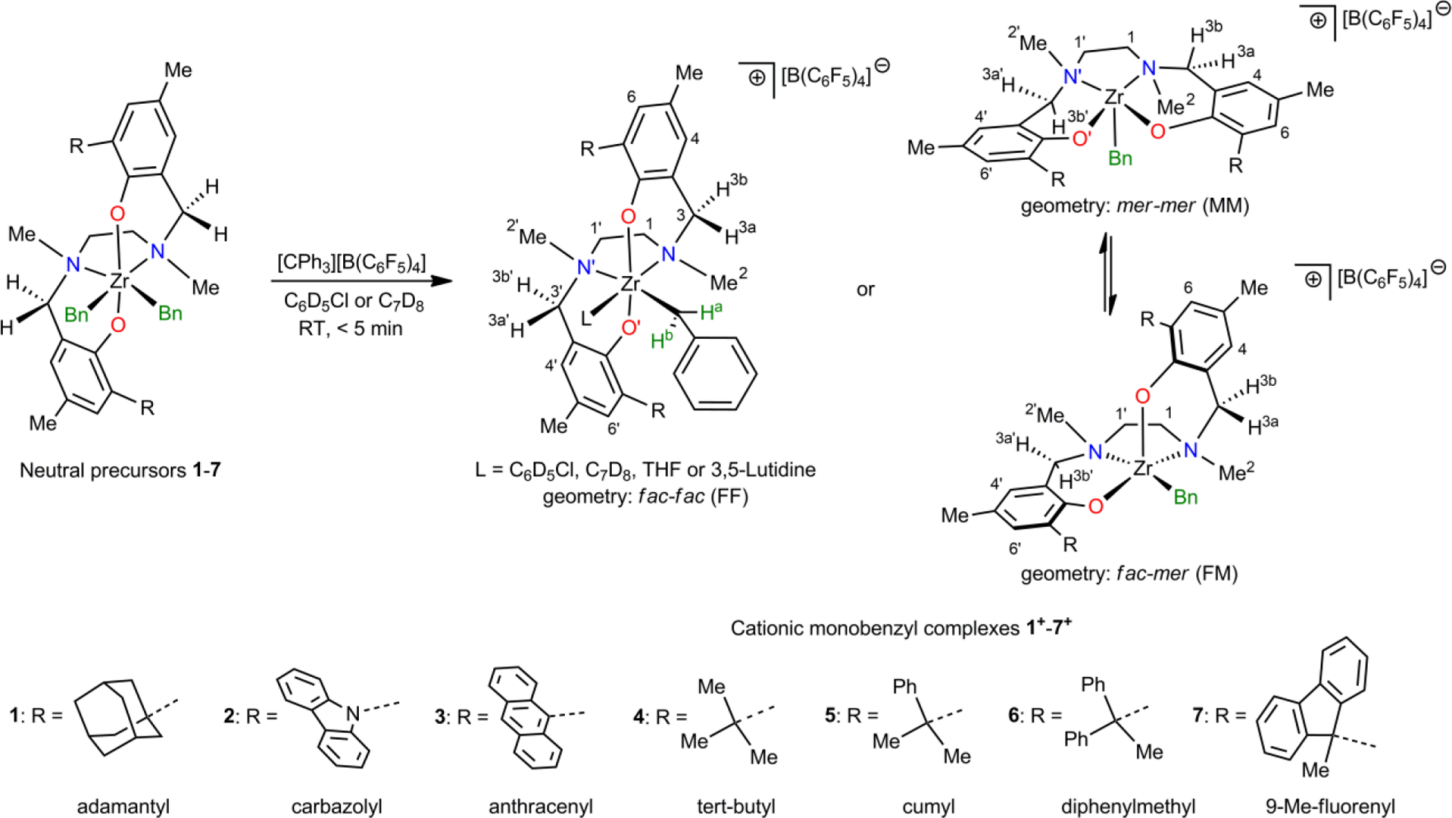
Complexes Investigated in This Work and Simplified Labeling Scheme for NMR Assignment.

Although usually considered as a bulk media affecting ion dissociation by means of their polarity, weakly coordinating solvents like toluene can play a more specific role by coordinating and stabilizing the cationic species, especially for “open” catalysts such as half-metallocenes^35,36^ and constrained-geometry catalysts (CGC).^37^ The notion that this is also occurring for relatively crowded cationic olefin polymerization complexes is fairly new. For instance, we have recently investigated how the specific catalyst-solvent interaction regulates ion pair symmetrization (IPS) barriers in metallocene complexes, i.e., how the mutual position exchange between the coordinative vacancy and the alkyl group in the first coordination sphere of the metal is influenced by solvent coordination.^14^ Extending the approach to post-metallocenes, we now report an in-depth analysis of seven Zr-Salan complexes (Scheme 1), differing in the *ortho*-substituent (R^1^) on the phenoxy-amine ring. These complexes mirror the broad tuning range in propene polymerization in this catalyst class, ranging from slow and living (**1**, **4**) to very highly active (**2**, **3**, **7**), from highly isotactic selective (**1**, **7**) to poorly isotactic selective (**2**) to chain-end controlled syndiotactic selective (**3**), and from (very) low (**1**–**6**) to high molar mass capability (**7**).

**1**–**7** were activated with trityl borate ( ([CPh_3_]^+^[B(C_6_F_5_)_4_]^−^, TTB, Scheme 1) in both C_7_D_8_ and C_6_D_5_Cl and investigated by in situ NMR spectroscopy, applying 1D and 2D NMR techniques, in order to determine the solution structure of the complexes. For the first time, solid-state structures of solvent-coordinated activated complexes were obtained, further supporting NMR findings in solution. NMR dynamics and DFT studies elucidate the solvent role. Importantly, diagnostic ^13^C-NMR chemical shift differences for the N*Me* atoms in different isomers uncovered for LMBn^+^ complexes (L = ligand) allow, for the first time, NMR identification of the predominant resting state isomer for LM-polymeryl^+^ species.

## Results and Discussion

### Synthesis and Characterization of Neutral Complexes **1–7**

Complexes **1**, **2**, **3**, **5**, and **7** were synthesized according to established literature protocols.^38,39^ The synthesis of complexes **4** and **6** is detailed in the Supporting Information.

**Scheme 2:**
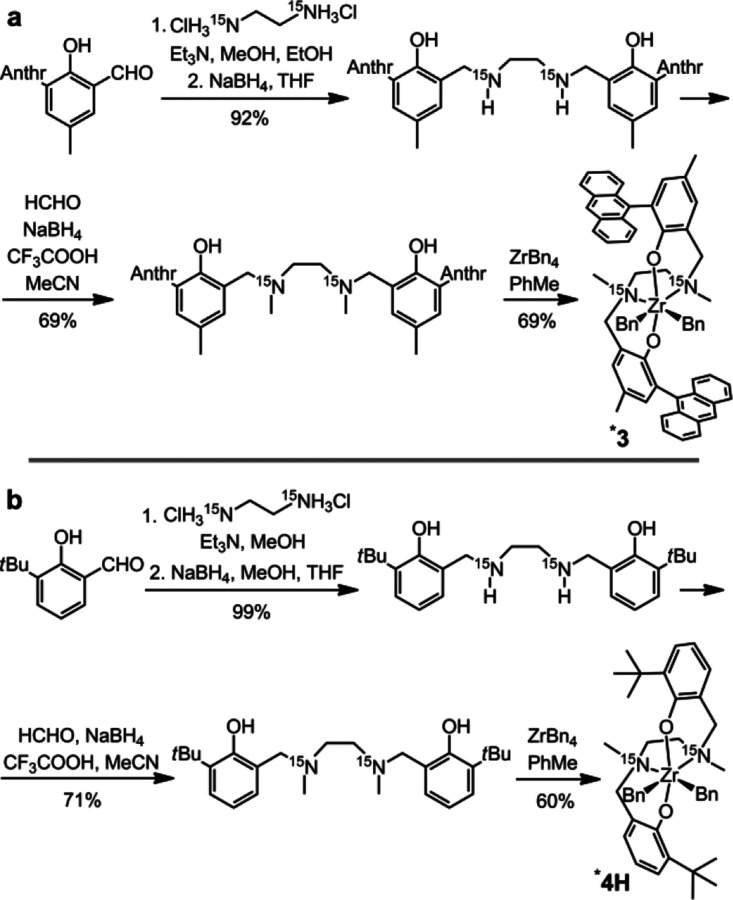
Three-Step Synthesis of the ^15^N-Enriched Complexes *3 and *4H

The most available and convenient starting material for ^15^N-labeled Salan ligands is ethylenediamine-^15^N_2_ dihydrochloride. The standard approach suggests the synthesis of a Salan ligand from DMEDA, paraformaldehyde, and a phenol in one step, albeit with a low or moderate yield (e.g., 15% for the ligand for **3**(38)). Since the three-step synthesis of DMEDA-^15^N_2_^40^ itself would lead to inevitable losses of the expensive labeled precursor, an alternative method for ligand synthesis was developed. Thus, the ^15^N-enriched *3 could be synthesized in 45% yield over three steps (Scheme 2a). Yields for the ^*t*^Bu-substituted **4** could not be sufficiently improved to allow economical synthesis of the ^15^N-enriched complex. However, the closely related *para*-H derivative *4H was synthesized in 43% yield over three steps (Scheme 2b).

### Propene Polymerization Using Complexes **1–7**

Complexes **1**–**7** were tested in propene polymerizations under rigorously identical conditions, using tri-*iso-*butyl aluminum (TiBA) as scavenger and TTB as activator, at *T*_p_ = 60 °C and *p*_propene_ = 6.6 bar, in a high-throughput experimentation setup (Freeslate parallel pressure reactor setup with 48 reaction cells (PPR48), fully contained in a glove-box).^41−45^

Polymerization procedures are detailed in the Experimental Section. Complexes **4** and **6** were explicitly tested for the purpose of this study; the results for complexes **1**–**3**, **5**, and **7** are taken in part from ref (46). The main polymerization results are summarized in Table 1.

**Table 1:** Propene Polymerization Results with Precatalysts **1–7** and TiBA/TTB as Activator/Scavenger at *T*_p_ = 60 °C and *p*_propene_ = 6.6 Bar^a^

precatalyst	*R*_p_^b^	σ^d^	*M*_n_^e^	*M*_w_^e^	PDI
**1**	0.04	0.997_5_	3.1	5.4	1.7
**2**	21.3	0.80	1.3	1.8	1.5
**3**	16.7	c	1.5	2.3	1.5
**4**	0.01	0.93	0.7	0.8	1.2
**5**	0.15	0.986	5.1	8.6	1.7
**6**	0.07	0.974	3.0	4.9	1.6
**7**	19.8	≥0.9998	69	143	2.1

a Other experimental conditions: 5 mL toluene, 5 μmol TiBA, [TTB]/[Zr] = 0.9.

b In kg mmol_Zr_^–1^ h^–1^

c Chain-end controlled syndiotactic polypropylene

d Enantioselectivity of propene insertion, calculated according to the enantiomorphic site model.^47^

e In kDa. All datapoints are averages of duplicate experiments, for further details see Supporting Information.

The broad performance tuning range for Salan catalysts is evident in the results presented in Table 1. Productivities span three orders of magnitude; polypropylene stereoregularity covers the whole range, from chain-end controlled syndiotactic to near perfect site-controlled isotactic. Molar mass capability ranges from *M*_n_ values of 0.7 kDa (**4**) to 69 kDa (**7**).

### Structures of Mono-Benzyl Cationic Species from **3** and **4**

Complexes **3** and **4** were activated with one equivalent of TTB in C_7_D_8_ and C_6_D_5_Cl and analyzed with in situ NMR spectroscopy to investigate substituent and solvent influences on the structure and dynamics of the cationic species. Benzyl group abstraction cleanly forms the mono-benzyl cationic species (Scheme 1); the solution geometry results from the interplay of ligand and solvent coordination strength.^36,48^ The cationic species **3**^+^ (“fast”) and **4^+^** (“living”) show distinctly different polymerization behaviors (Table 1) and were therefore natural starting points to investigate their solution structure. Their ^15^N-enriched derivatives, *3 and *4H, were synthesized to facilitate speciation of the active species.

#### Activation of **3**

^1^H and ^13^C NMR spectra of **3^+^** in C_6_D_5_Cl show extensive peak broadening at 298 K; however, at 233 K, distinct ^1^H and ^13^C NMR resonances for each half of the tetradentate ligand can be resolved, revealing the loss of *C*_2_ symmetry (Figure S17). The resonances of each half can be grouped following the ^1^H,^1^H and ^1^H,^13^C scalar correlations. Two singlets at δ_H_ = 1.93 and 1.37 ppm, both integrating to three protons each, show scalar correlations with carbons at δ_C_ = 47.7 and 45.7 ppm, respectively; these are assigned to the NMe moieties. We arbitrarily indicate as Me^2^ the methyl group having a signal at 1.93 (47.7) ppm and as Me^2^′ the one with resonances at 1.37 (45.7) ppm (see Scheme 1 for the NMR labeling of protons and carbons). Me^2^ (δ_C2_ = 47.7 ppm) shows ^1^H,^13^C long-range coupling with two carbons at δ_C_ = 65.0 ppm and 54.7 ppm, which in turn shows a single bond correlation with two doublets at 4.02 and 2.99 ppm, and at δ_H_ = 2.97 and 1.79 ppm, respectively (Figures S18 and S19). The carbon resonance at 54.7 ppm does not display any additional long-range coupling with aromatic protons and is therefore assigned to carbon C^1^ of the ethylene bridge. Consequently, the signals at δ_H_ = 2.97 and 1.79 ppm belong to H^1a^ and H^1b^. H^1a^ appears as a doublet of doublets with ^2^*J*_HH_ ≈ ^3^*J*_HH_ ≈ 13.6 Hz and, based on Karplus relationships, is assigned to the proton in axial position. A long-range coupling between the carbon at δ_C_ = 65.0 ppm and the aromatic proton resonance at δ_H_ = 6.80 ppm allows assignment of the former to C^3^ and the latter to H^4^. Thus, the signals at δ_H_ = 4.02 and 2.99 ppm are due to H^3a^ and H^3b^. Starting from H^4^, the remaining resonances of the phenolate and anthracenyl moieties are assigned by crossing the data from ^1^H,^1^H COSY, ^1^H,^13^C HSQC, and ^1^H,^13^C HMBC NMR experiments (Figures S18–S20). Extensive overlaps of aromatic resonances of the anthracenyl substituent prevent full assignment of this moiety. Similarly, starting from the Me^2^′ resonance (δ_H_ = 1.37 ppm, δ_C_ = 45.7 ppm), the resonances of the other half of the ligand could be assigned (H^1a^′ = 2.57 ppm; H^1b^′ = 1.44 ppm; C^1^′ = 51.7 ppm; H^3a^′ = 2.04 ppm; H^3b^′ = 2.25 ppm; C^3^′ = 62.0 ppm; H^4^′ = 6.54 ppm, and so on; see Supporting Information).

The benzyl ligand appears coordinated in η^2^ fashion as indicated by the observation of seven different resonances: two are expected for the CH_2_ benzyl moiety (δ_H_ = 2.16 ppm (H^a^) and 1.01 ppm (H^b^); δ_Cbenzyl_ = 71.2 ppm) based on symmetry considerations, but the observation of five distinct aromatic resonances (δ_H_ = 6.39 ppm, H^p^, 5.89 ppm, H^o^′; 5.73 ppm, H^m^; 5.61 ppm. H^m^′; 4.01 ppm, H^o^) is diagnostic for a restricted rotation around the CH_2_–C_*ipso*_ bond. The shift of C_*ipso*_ from 154.3 ppm, in the neutral **3** precursor, to 128.8 ppm in **3^+^**, and the relatively small ^2^*J*_HH_ (∼7 Hz) between H^a^ and H^b^, lend further support.

**Scheme 3:**
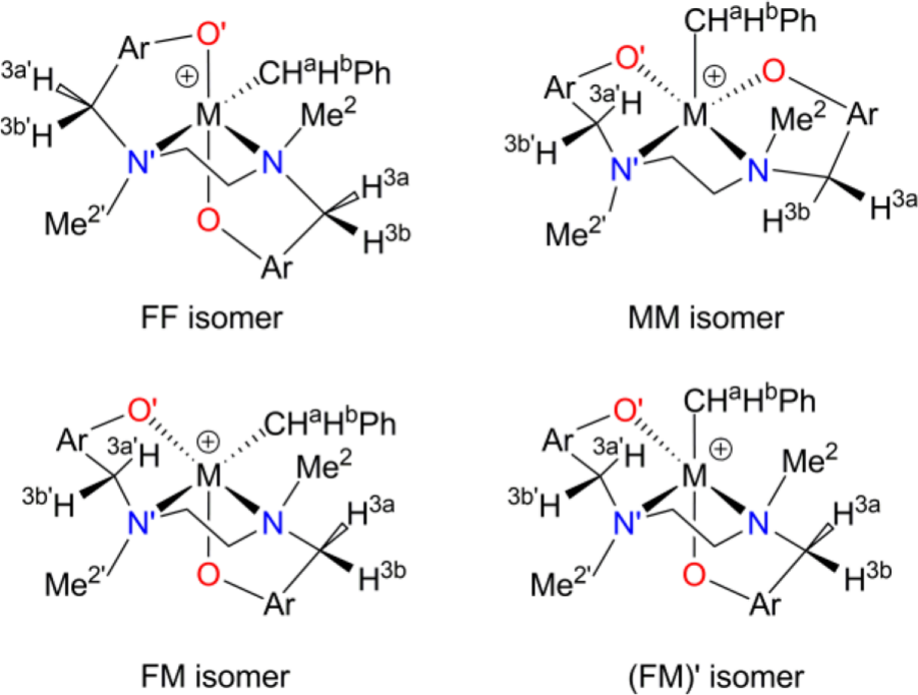
Limiting Coordination Modes of the [ONN′O′] Ligand in Alkyl Monocationic Group 4 Metals.

The coordination mode of the tetradentate [ONN′O′] ligand around Zr, i.e., the geometry adopted in solution by complex **3^+^**, can be inferred by analyzing relevant dipolar interactions in the ^1^H ROESY NMR spectrum with the help of DFT optimized structures. For cationic group 4 metals, in general, the [ONN′O′] ligand can coordinate with three limiting modalities (Scheme 3); FM and FM′ isomers differ in the position of the benzyl ligand, *trans* to N′ (in FM) and *trans* to O (in FM′).

**Figure 2:**
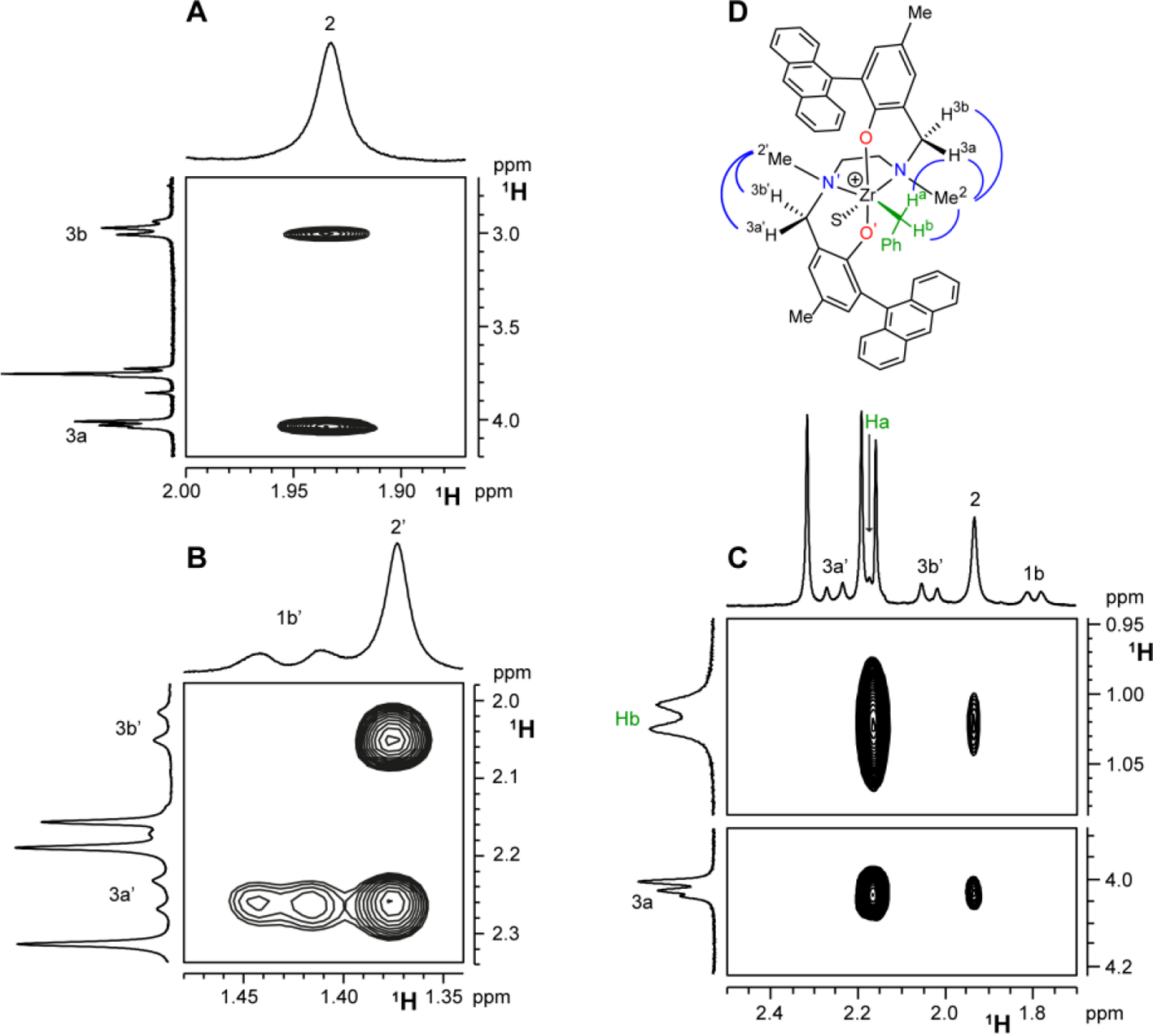
Three sections of the ^1^H ROESY NMR spectrum of **3^+^_FF_** (C_6_D_5_Cl, 233 K) showing (A) the NOE contacts, of similar intensity, of Me^2^ with both H^3a^ and H^3b^; (B) the NOE contacts, of similar intensity, of Me^2^′ with both H^3a^′ and H^3b^′; (C) the NOE contacts between H^b^ and Me^2^ and between H^a^ and H^3a^. Relevant dipolar interactions are summarized in D by blue arches.

Selective H^a^/H^3a^ and H^b^/Me^2^ dipolar interactions observed in the ^1^H ROESY NMR spectrum (Figure 2) indicate that both benzylic protons (H^a^ and H^b^) are spatially close to protons of one half of the ligand, clearly inconsistent with both MM and FM′ geometries.

**Scheme 4:**
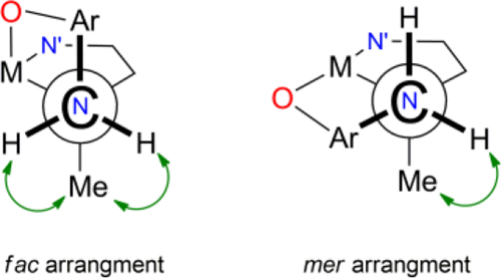
Simplified Newman Projection Showing the Spatial Relationship of NMe with respect to its adjacent CH_2_ Protons in the *fac* or *mer* Arrangements of the Ligand.

Distinguishing structure FF from the FM geometry is less straightforward. DFT structures reveal a general feature: when the three NN′O′ (or N′NO) arms of the ligand are arranged in a *fac* configuration, the methyl group on N (or N′) is equally distant from its adjacent H^3^ protons, whereas if the three NN′O′ (or N′NO) arms are arranged in the *mer* configuration, one of the H^3^ protons becomes antiperiplanar to the Me moiety and thus much more distant (Scheme 4).

In the case of **3^+^** in C_6_D_5_Cl, both Me^2^ and Me^2^′ show dipolar interaction of very similar intensity with their corresponding H^3a^, H^3b^ and H^3a^′, H^3b^′ protons, thus indicating that each ligand half adopts a *fac* configuration. The single species observed in solution has FF geometry (Figure 2). This is corroborated by the lack of NOE contacts between Me^2^/Me^2^′ with H^3^ protons on the other side of the ligand. Selective H^a^/H^o^ and H^b^/H^o^′ NOE contacts observed within the benzylic fragment agree with the proposed η^2^ coordination mode. The relative orientation of the benzyl ligand can be inferred from the lack of dipolar interactions between aromatic protons of the benzyl moiety and aliphatic resonances of the ligand, indicating that the aromatic ring points toward the formally vacant coordination site.

Whether a C_6_D_5_Cl solvent molecule occupies the “vacant” site cannot be directly inferred from NMR data but is confirmed by X-ray analysis on a single crystal of **3^+^_FF_** (vide infra). NMR structural analysis of **3^+^_FF_** generated in C_7_D_8_ is more complex because ^1^H NMR resonances of the benzyl moiety are not detected, likely as a result of σ-bond metathesis (SBM) with the deuterated solvent leading to a fully deuterated benzyl ligand.^49^

Generating **3^+^_FF_** in C_7_H_8_, isolating it by precipitation with *n*-pentane, and redissolving it in C_7_D_8_ at 233 K, allows to observe aromatic benzyl resonances at chemical shifts similar to those in C_6_D_5_Cl (Figure S21). Indicative for very low SBM barriers, those resonances disappear quickly even at low temperatures, hampering full NMR elucidation. Proton and carbon NMR resonances of the ONN′O′ ligand of **3^+^_FF_** do not differ significantly from those observed in C_6_D_5_Cl. Both Me^2^ and Me^2^′ show dipolar interactions of very similar intensity with their corresponding H^3a^, H^3b^ and H^3a^′, H^3b^′ protons, implying that the C_7_D_8_ complex also adopts the **3^+^_FF_** geometry (see Supporting Information). This is further supported by single crystal X-ray analysis.

^15^N NMR data of the labeled complex *3^+^_FF_ in C_6_D_5_Cl and C_7_D_8_ and related Lewis base stabilized cationic species, *3^+^_FF_ -THF and *3^+^_FF_-Lut (Lut = 3,5-lutidine, see Supporting Information) in C_6_D_5_Cl, were recorded to further elucidate coordination effects. (Table 2).^50^ The chemical shift of the nitrogen atom *cis* to the benzyl group is sensitive to the nature of L (L = Lewis base or solvent) decreasing with increasing coordination strength of L: δ_N_ = 49.47, 51.35, 54.09, and 56.77 ppm for L = Lut, THF, C_6_D_5_Cl, and C_7_D_8_, respectively. The chemical shift of N′, the nitrogen atom *trans* to the benzyl group, is only minimally affected by the nature of L: δ_N′_ = 49.75, 51.17, 51.09 and 51.86 ppm for L = Lut, THF, C_6_D_5_Cl, and C_7_D_8_, respectively. These observations are fully consistent with the hypothesis that a donor molecule remains coordinated to the metal in complexes **3^+^_FF_ -L** (L = C_7_D_8_, C_6_D_5_Cl, THF and Lut).

**Table 2:**
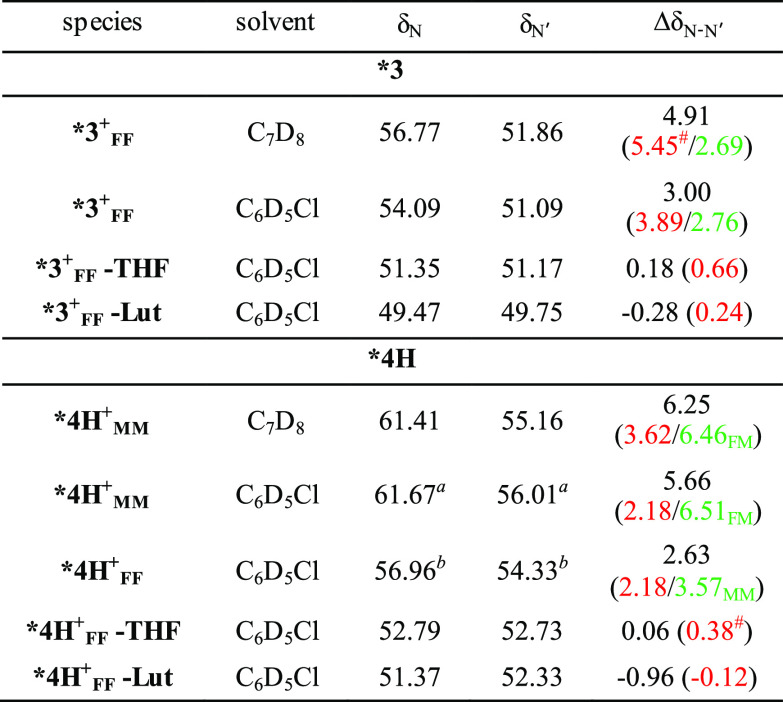
^15^N NMR Chemical Shifts of N and N′ (δ_N_ and δ_N′_, Externally Referenced to Ammonia in ppm) for Selected Cationic Complexes Derived from *3 and *4H and Experimental (Black) and Computed (Red for “FF” and Green for “MM” or “FM” Geometries) Chemical Shift Difference between N and N′ (Δδ_N-N′_)

a At 273 K where a single species of *4H^*+*^ is dominating in solution (vide infra).

b At 218 K for the second species of *4H^+^ present in solution (vide infra).

#### Activation of **4**

**Figure 3:**
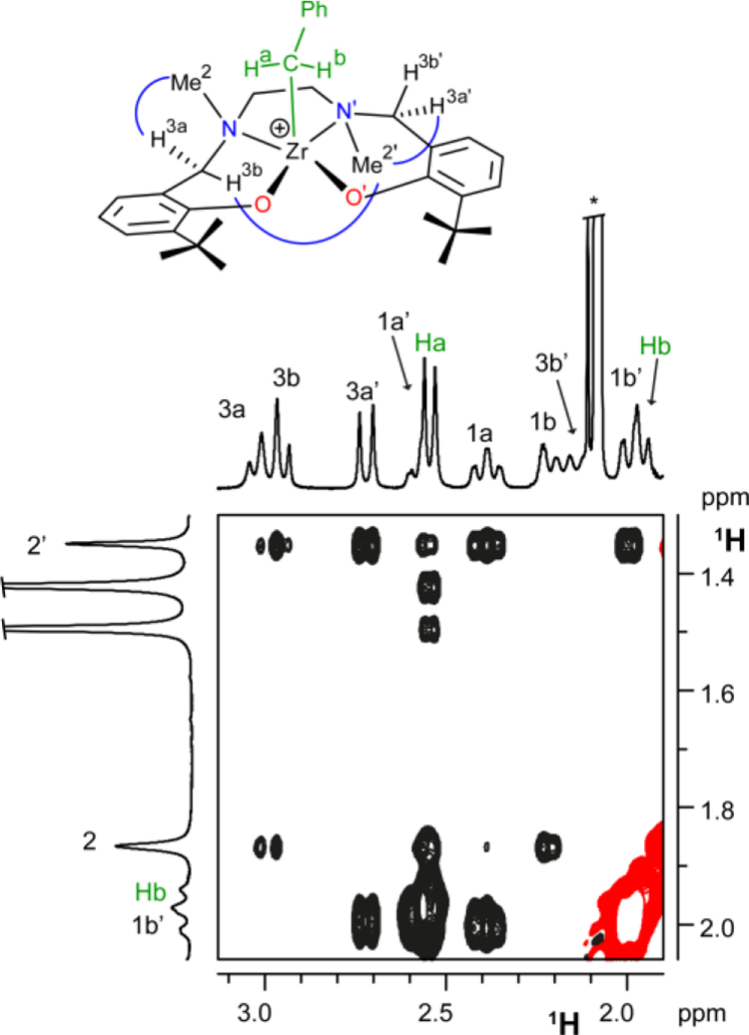
Section of the ^1^H ROESY NMR spectrum of *4H^+^_MM_ (C_7_D_8_, 263 K) showing (a) the selective interactions between Me^2^′ and H^3a^′; (b) the selective interactions between Me^2^ and H^3a^; (c) the strong interaction between Me^2^′ and H^3b^. Relevant dipolar interactions are summarized in the structure depiction by blue arches.

The solution structure of **4^+^**, and that of the related ^15^N labeled complex *4H^+^, were investigated using the same methodological approach; aside from obvious differences, both complexes show nearly superimposable NMR spectra. For temperatures higher than 253 K, **4H^+^** displays several NMR features that are different from those of **3^+^_FF_**. Firstly, well-resolved spectra are obtained already at 268 K, both in C_6_D_5_Cl and C_7_D_8_, suggesting a lower degree of dynamicity. Secondly, both Me^2^ and Me^2^′ show dipolar interactions (Figure 3) with only one adjacent H^3^ proton, suggesting each half of the ligand preferentially adopts a *mer* configuration (Scheme 4). This is further supported by the observation of a relatively strong dipolar interaction between Me^2^′ on one side of the ligand and H^3b^, on the other side (Figure 3). Finally, only three aromatic resonances are observed for the benzyl ligand, indicating a fast rotation around the C_*ipso*_–CH_2_ bond consistent with a η^1^ coordination modality.

N and N′ of *4H^+^ resonate at significantly higher frequencies with respect to *3^+^_FF_ (C_7_D_8_: δ_N_ = 61.41, δ_N′_ = 55.16 ppm; C_6_D_5_Cl: δ_N_ = 61.67, δ_N′_ = 56.01 ppm), both in C_7_D_8_ or C_6_D_5_Cl. The chemical shifts are scarcely affected by the nature of the solvent, in agreement with the lack of solvent coordination. The higher values of δ_N_ can be attributed to the switch of N from *trans* to solvent (as in *3^+^_FF_) to *trans* to oxygen. The values of δ_N′_, instead, suggest that this atom could be in an intermediate situation, somewhere between *trans* to benzyl (as in *3^+^_FF_) and *trans* to oxygen; both MM and FM limiting geometries (Scheme 3) could contribute to the actual structure of cationic complex *4H^+^_MM_ in solution and, on average, the actual geometry may be described as a distorted trigonal bipyramid having the N and O′ arms in the apical positions.^31,51^

Alternatively, the actual geometry might be so distorted that limiting “ideal geometries” are a bad fit. Importantly, ^15^N NMR data trends suggest substantial structural modifications on passing from *4H^+^ to *4H^+^-THF and *4H^+^-Lut (Table 2 and Supporting Information). ^15^N chemical shifts for *4H^+^_FF_-THF (δ_N_ = 52.79 ppm; δ_N′_ = 52.73 ppm) and *4H^+^_FF_-Lut (δ_N_ = 51.37 ppm; δ_N′_ = 52.33 ppm) are found at lower frequencies, with values very similar to those observed for complexes *3^+^_FF_-THF and *3^+^_FF_-Lut, indicating stronger Lewis bases trigger a switch of geometry.

#### Solid-State Structure of *3^+^_FF_

Crystals of *3^+^_FF_ suitable for X-ray analysis were obtained by layering *n*-pentane on top of concentrated solutions of *3^+^_FF_ in both C_7_D_8_ and C_6_D_5_Cl. Although single crystal X-ray structures for neutral Salan complexes are known,^25,33,52^ to the best of our knowledge, this is the first example of a solid-state structural analysis for a cationic Salan complex.

**Figure 4:**
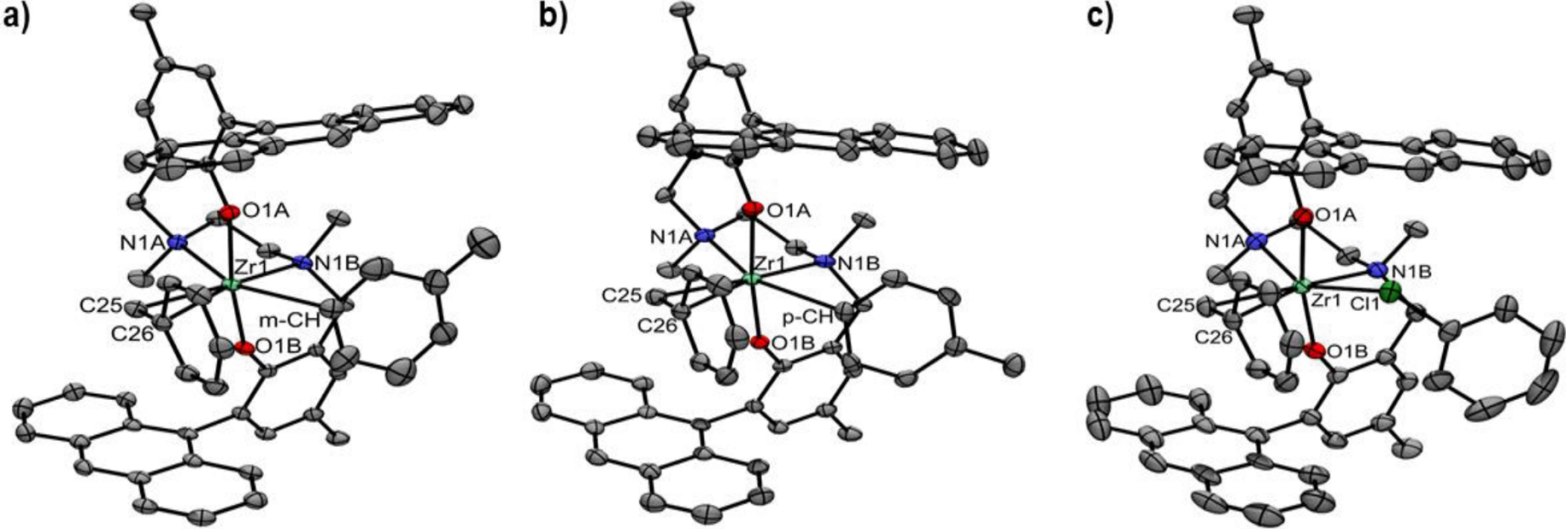
X-ray structures of: (a) *3^+^_FF_·C_7_D_8_ (*meta-*C interaction), (b) *3^+^_FF_·**C_7_D_8_** (*para-*C interaction), (c) *3^+^_FF_·**C_6_D_5_Cl**. Ellipsoids drawn at the 50% probability level; hydrogen atoms have been omitted for clarity.

The unit cell of *3^+^_FF_·C_6_D_5_Cl contains two enantiomeric cations with coordination environments of the Zr centers exhibiting minimal differences. The octahedral coordination geometry is slightly distorted with the tetradentate [ONN′O′] ligand coordinated in the *fac*-*fac* configuration (Figure 4). The O–Zr–O′ angles amount to 168.9°; the O–Zr–N′ (93.4–93.9°), and the O′–Zr–N (100.8–100.9°) angles are found close to 90°. The benzyl ligand and a chlorobenzene molecule in *cis* relative position complete the coordination environment. Similarly to other Zr(IV) complexes,^48^ the solvent molecule is coordinated to the metal via the chlorine atom (Zr–Cl 2.779 Å).

Metrical parameters indicate η^2^ hapticity^53^ of the benzyl ligand with the Ph ring pointing away from the ligand framework, toward the coordinated chlorobenzene molecule. Indeed, for both cations, the difference between the Zr–CH_2_ distances (2.266(3) Å; 2.272(4) Å) and the Zr–C_*ipso*_ distances (2.522(3) Å; 2.540(4) Å) are lower than 0.5 Å, whereas the Zr–CH_2_–C_*ipso*_ angles (81.90 deg.; 82.77 deg) are much lower than 97 deg.^53^ Consistently, the Zr–O and Zr–O′ distances (*trans* to each other) are identical within the experimental error (1.973(2) and 1.971(2) Å). The Zr–N (*trans* to the solvent) distances, 2.408(3) and 2.404(3) are noticeably shorter than the Zr–N′ (*trans* to the benzyl) distances, 2.483(3) and 2.485(3) Å).

In the case of *3^+^_FF_·C_7_D_8_, the unit cell contains four cation-anion pairs. Again, Zr centers in *3^+^_FF_C_7_D_8_ display a slightly distorted octahedral geometry with the tetradentate [ONN′O′] ligand framework coordinated in *fac-fac* configuration. The coordination environment is completed by an η^2^ coordinated benzyl ligand and a toluene molecule in *cis* relative position. Zr–C_ipso_ distances are slightly longer with respect to *3^+^_FF_·C_6_D_5_Cl (2.593(4), 2.583(4), 2.574(4), and 2.582(4) Å). The four cations differ in the orientation of the toluene molecule, not in absolute configuration: in three of them, toluene is interacting with one of the *meta* carbons (average Zr–C_*meta*_ distance of 2.89 Å, Figure 4a), while in the fourth cation the *para* carbon is closer to Zr (Zr–C_*para*_ distance of 2.87 Å, Figure 4b). In line with trends of ^15^N NMR chemical shifts, the Zr–N′ (*trans* to the benzyl) distances are similar to those of *3^+^_FF_·C_6_D_5_Cl, whereas the Zr–N (*trans* to the solvent) are, on average, slightly longer (2.405(3), 2.416(3), 2.427(3), 2.422(3) Å).

Overall, the X-ray data of *3^+^_FF_ confirms the NMR findings: the tetradentate [ONN′O′] ligand framework is coordinated in the *fac-fac* configuration both in solution and the solid state; coordination of a solvent molecule stabilizes this geometry. Unfortunately, all attempts to obtain single crystals of **4^+^** suitable for X-ray analysis have been unsuccessful so far.

#### DFT Insights into the Salan Isomer Equilibria

The findings discussed above clearly indicate that the structure adopted in solution by the active cationic species is not always the same. It was hypothesized that Salan complexes are affected by a MM/FF pre-equilibrium. This is the case for **4** in weakly coordinating solvents, a slow catalyst also studied previously.^29^ However, the results for **3** (and **4** in strongly coordinating solvents) indicate that this equilibrium can be shifted to FF and the actual solution structure is strictly related to the interplay between the substituents on the [ONN′O′] ligand and the nature of the solvent.

We interrogated the equilibria between the different isomers of **3^+^** and **4^+^** computationally employing Grimme’s conformer rotamer ensemble sampling tool (CREST)^54^ followed by final DFT optimization at the TPSSh-D_zero_(PCM)/TZ//TPSSh/DZ level of theory.^55−65^ Experimental evidence unequivocally points to *3^+^_FF_(solvent) as the lowest conformer in toluene and chlorobenzene. Including the solvent in the coordination sphere, we identified one FF conformer for *3^+^_FF_-C_6_D_5_Cl and two nearly isoenergetic FF conformers for *3^+^_FF_-C_7_D_8_, differing in the toluene orientation (*meta*- or *para*-C binding), mirroring the X-ray findings. In the toluene case, binding is weak as an FF isomer with toluene in the outer coordination sphere was found to be only 1.3 kcal/mol higher in Gibbs free energy than the isomer with toluene in the first coordination sphere. Predicted ^15^N NMR chemical shifts differences match experimentally observed ones nicely (see Table 2) and agree with the solvent-coordinated complex. Gas phase sampling of the conformational space in the absence of solvent reveals four nearly isoenergetic isomers: two FF, one FM and one MM isomers.

**Figure 5:**
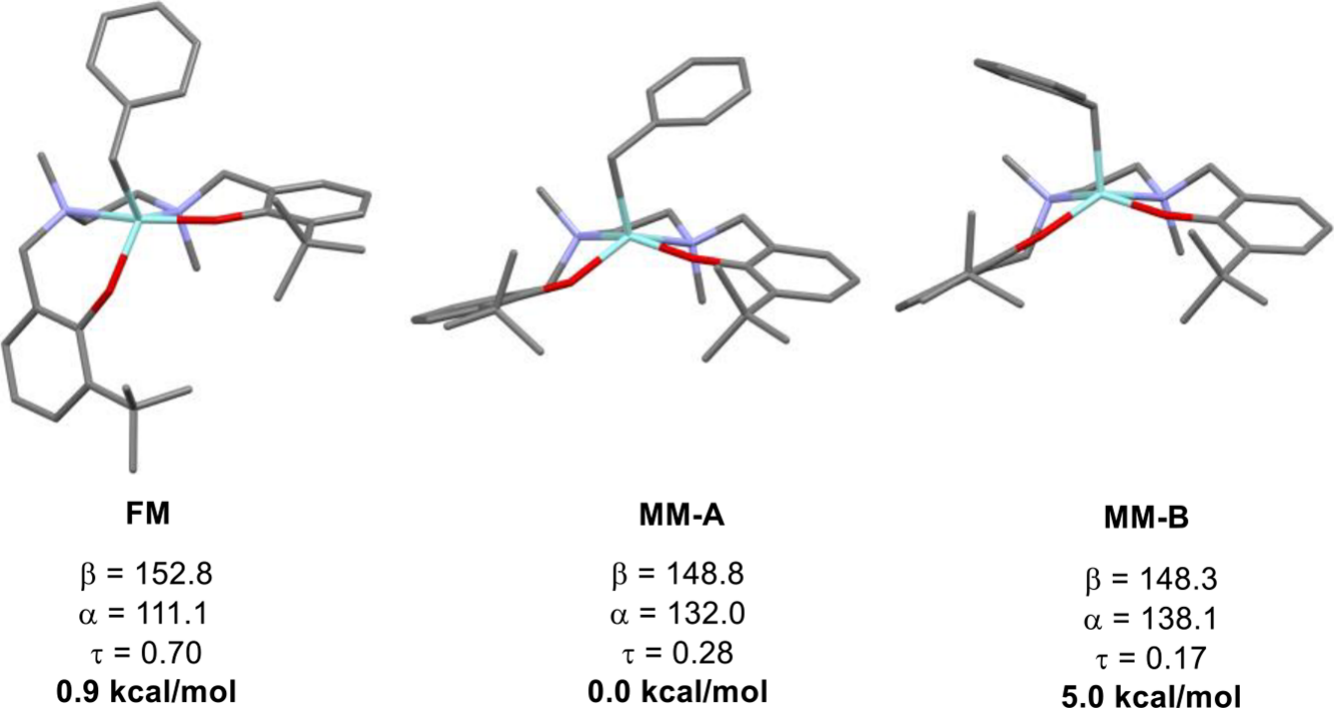
Key bond angles, geometry index τ, and relative Gibbs free energies at T = 298 K for selected FM and MM isomers of **4**^+^.

The situation is less clear-cut for **4^+^**, both computationally and experimentally. Gas phase sampling of the conformational space indicates six non-solvent-coordinated isomers within a Gibbs free energy window of 5.2 kcal/mol, three FM, two MM, and one FF isomer. The two low-energy isomers are MM (0.0 kcal/mol) and FM (0.9 kcal/mol); predicted ^15^N-NMR chemical shifts are more comparable with the FM isomer. All isomers are significantly distorted from ideal geometries with Addison τ parameters,^66^ ranging from 0.17 to 0.70, indicating significant distortion from ideal pyramidal (MM, τ = 0) and trigonal bipyramidal (FM, τ = 1) geometries (Figure 5). It should be noted that the less distorted MM isomer (τ = 0.17) is 5.0 kcal/mol higher in energy than the more distorted one (τ = 0.28). To identify general trends, we compared predicted ^15^N-NMR shift differences between the N and N′ atoms for solvent-coordinated and non-coordinated isomers of **3^+^**, **4^+^** and **7^+^**. As observed experimentally, predicted chemical shift differences (Δδ_Ν-Ν′_). decrease with donor strength of the solvent in solvent-coordinated FF complexes. However, similarly, Δδ_Ν-Ν′_ becomes smaller in the MM isomers, the less distorted the isomer is according to the τ parameter. FM isomers also show highly flexible shift differences.

Likewise, absolute shifts are also highly flexible. It appears that the complex interplay between benzyl group orientation, agostic interactions and relative orientations of backbone Ph, and linker geometry precludes identification of general trends that would allow positive determination of the prevalent isomer solely based on ^15^N chemical shifts.

#### NMe Carbon Chemical Shifts as Diagnostic Indicators for [ONN′O′]MR^+^ Complex Geometry

Extension of the NMR methodological approach described for **3^+^_FF_** and **4^+^_MM_** to the cationic species derived from the other precursors **1**–**2**, **5**–**7** proved to be difficult; ^1^H NMR spectra often exhibit broad and superimposed resonances preventing unambiguous NOE contact detection.

**Figure 6:**
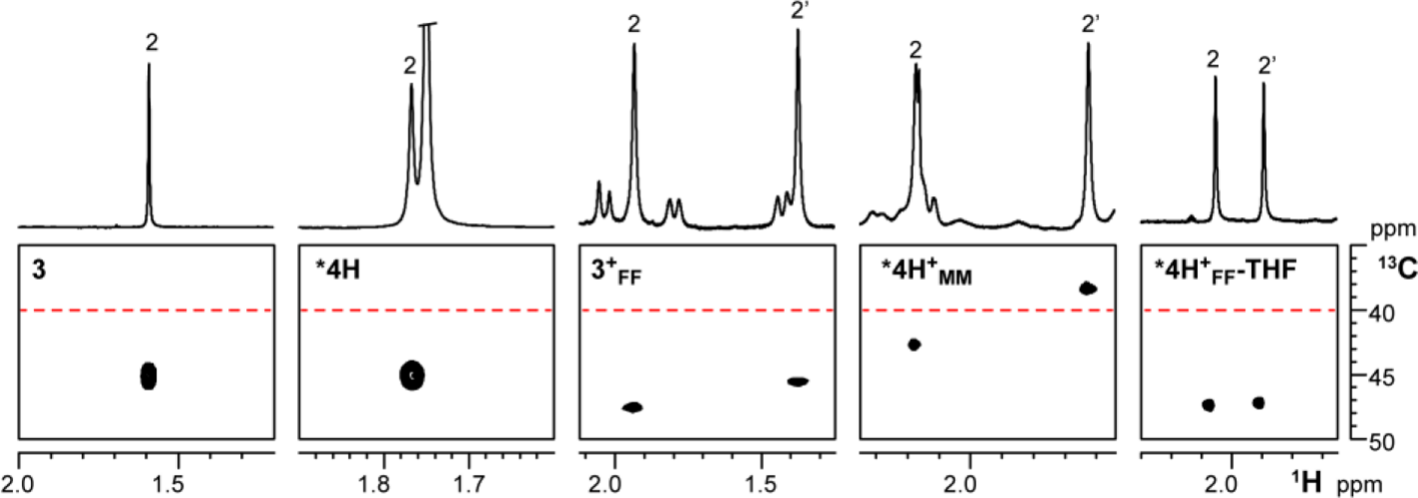
Sections of the ^1^H,^13^C HSQC NMR spectra of **3** (C_6_D_6_, 298 K), *4H (C_6_D_6_, 298 K), **3^+^_FF_** (C_6_D_6_Cl, 233 K) *4H^+^_MM_ (C_6_D_5_Cl, 263 K) and *4^+^_FF_-THF (C_6_D_5_Cl, 292 K) showing the relationship between the coordination geometry adopted by the [ONN′O′] ligand and the ^13^C NMR chemical shifts of C^2^ and C^2^′.

^13^C NMR chemical shifts of the NMe moieties appear to follow a rather regular trend. C^2^ and C^2^′ resonate at δ_C_ values well above 40 ppm (Figure 6) for all neutral and cationic complexes with FF geometry. In contrast, for both complex **4^+^_MM_** and *4H^+^_MM_, C^2^′ appears at frequencies lower than 40 ppm (δ_C2′_ in the range 38.4–37.6 both in C_7_D_8_ and C_6_D_5_Cl for *T* > 253 K, see Table S1 and Figure 6). For **8^+^_MM_**, a complex analogous to **4^+^_MM_** but having an OMe substituent in *para* position of the phenolate rings and a Zr–Me instead of a Zr–Bn moiety, previously shown to adopt the MM geometry based on ^19^F, ^1^H HOESY NMR data and DFT calculations, δ_C2_ and δ_C2′_ are observed at 42.7 ppm and at 37.6 ppm, respectively, allowing to exclude anisotropic effects originating from the benzyl ligand. Thus, ^13^C NMR chemical shifts of C^2^ and C^2^′, easily measurable NMR parameters, may indeed discriminate FF from MM/FM geometries.

DFT prediction of ^13^C-NMR NMe chemical shifts confirms their diagnostic value. For **3^+^**, **4^+^**, and **7^+^**, 24 non-solvent-coordinated and 14 solvent-coordinated isomers were identified overall. All identified FF isomers (with or without solvent) show predicted ^13^C-NMR shifts >43 ppm, while all MM and FM isomers show one <43 ppm, predominantly <40 ppm. In FF isomers, both methyl groups on the nitrogens are oriented toward the back of the catalyst, therefore pointing away from the empty site. Rearrangement from *fac* to *mer* of one of the fragments orients its methyl toward the empty site, accompanied by a lowering of the chemical shift. In the MM isomer, only one of the methyls is oriented toward the empty site; the other one remains *anti* to the empty site, and the chemical shift is unaffected. Therefore, the observation of a chemical shift <40 ppm is diagnostic for the complex not being in *fac*-*fac* geometry, although exact determination of its geometry (MM or FM or FM′, Scheme 3) is not possible solely based on ^13^C-NMR NMe chemical shifts. Given the insight from experiments and computations discussed above, it appears highly likely that the prevalent geometry for the non *fac*-*fac* isomer is very distorted from the idealized situation depicted in Scheme 3. For simplicity (and continuity with previous literature), we continue to refer to it as MM.

^13^C NMR chemical shifts of C^2^ and C^2^′ for neutral and cationic complexes in different solvents are collected in Table S1; a summary is reported in Table 3. Similarly to **3^+^_FF_**, complex **2^+^_FF_**, bearing an *N*-carbazolyl substituent directly bonded to the phenolate rings, adopts the FF geometry irrespective of the type of solvent. In contrast, complexes **1^+^_MM_** and **5^+^_MM_-7^+^_MM_**, having an aliphatic substituent on phenolate rings, show δ_C2′_ lower than 40 ppm in C_7_D_8_, similarly to **4^+^_MM_**.

**Table 3:** Geometries of Activated Complexes Derived from **1**–**7**

complex	geometry (toluene)	geometry (C_6_D_5_Cl)	*R*_p_^a^
**1^+^**	FM/MM	FF	0.04
**2^+^**	FF	FF	21.3
**3^+^**	FF	FF	16.7
**4^+^**	FM/MM	FF < 248 K	0.01
**5^+^**	FM/MM	FF < 298 K	0.15
**6^+^**	FM/MM	FF < 268 K	0.07
**7^+^**	FM/MM	FF	19.8

a In kg mmol_Zr_^–1^ h^–1^.

**Figure 7:**
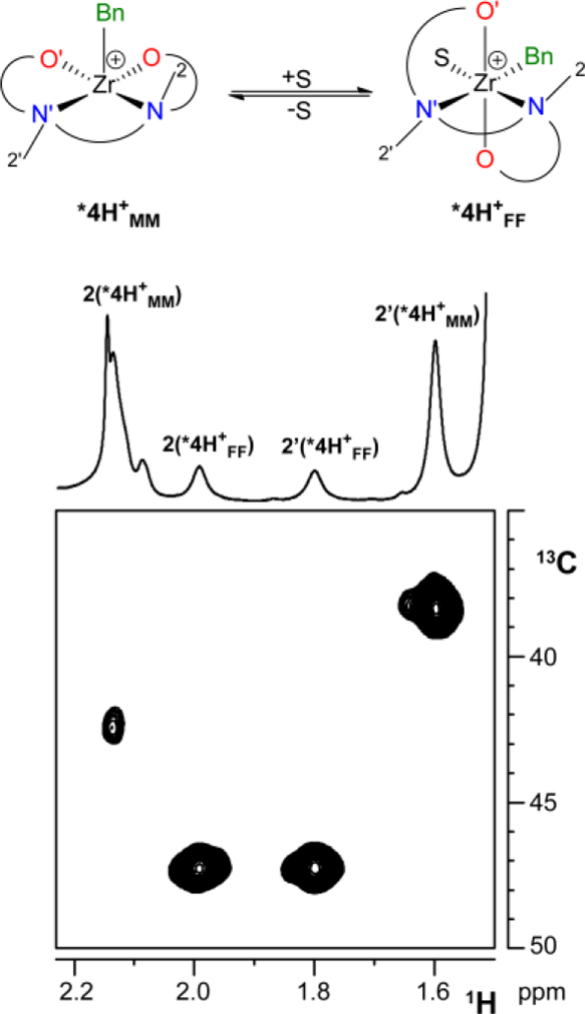
Section of the ^1^H,^13^C HSQC NMR spectrum of an equilibrium mixture of *4H^+^_FF_ and *4H^+^_MM_ (C_6_D_5_Cl, 243 K).

When passing from C_7_D_8_ to the more coordinating C_6_D_5_Cl, the behavior of cationic species derived from precursors **1**, **4**, **5**–**7** becomes more intricate, depending on the nature of the substituent and temperature. For instance, below 248 K in C_6_D_5_Cl, a new set of resonances appears in the ^1^H NMR spectrum of *4H^+^_MM_ (the same holds true for **4^+^_MM_**). Despite full NMR characterization being hampered by extensive broadening and spectral overlaps, the new species features two diagnostic singlets at δ_H_ = 1.98 and 1.80 ppm, which are scalarly coupled with carbon atoms resonating at 47.1 ppm (Figure 7).

These resonances are assigned to Me^2^ and Me^2^′ moieties and allow us to conclude that the new species is *4H^+^_FF_, which forms from *4H^+^_MM_ by solvent coordination. At 218 K, the chemical shift difference of Δδ_N-N′_ = 2.63 ppm (Table 2) corroborates this assignment as it is (a) much lower than for *4H^+^_MM_ and (b) similar to **3^+^_FF_** in the same solvent (Δδ_N-N′_ = 3 ppm). We will discuss the equilibrium details between *4H^+^_MM_ and *4H^+^_FF_ later.

On the other side, in C_6_D_5_Cl, complex **1^+^_FF_** displays δ_C2_ and δ_C2′_ higher than 40 in the whole investigated temperature range (223–298 K): possibly, the increase of the substituent steric bulk is sufficient to destabilize the MM geometry to the point that binding of C_6_D_5_Cl and switching to FF geometry become thermodynamically more favorable. Similar to **4^+^**, the Me^2^ and Me^2′ 13^C NMR chemical shifts are temperature dependent for cationic complexes **5^+^** and **6^+^** in C_6_D_5_Cl (See Supporting Information), indicating the prevalence of the solvent-stabilized **5^+^_FF_**/**6^+^_FF_** isomers and the five-coordinated **5^+^_MM_**/**6^+^_MM_** isomers, at low and high temperatures, respectively.

### Dynamics of Cationic Complexes in Solution

In solvent-stabilized *ansa*-metallocene cationic complexes, inversion of configuration at the metal center, i.e., site epimerization (SE) or IPS, occurs via a mechanism where solvent decoordination is rate-limiting.^67−69^ Cationic FF Salan complexes show some structural similarity with *ansa*-metallocene cations and solvent choice should affect these barriers as well. Then again, how these processes occur for systems adopting MM geometry and how they are related to the nature of the solvent is less clear.

**Figure 8:**
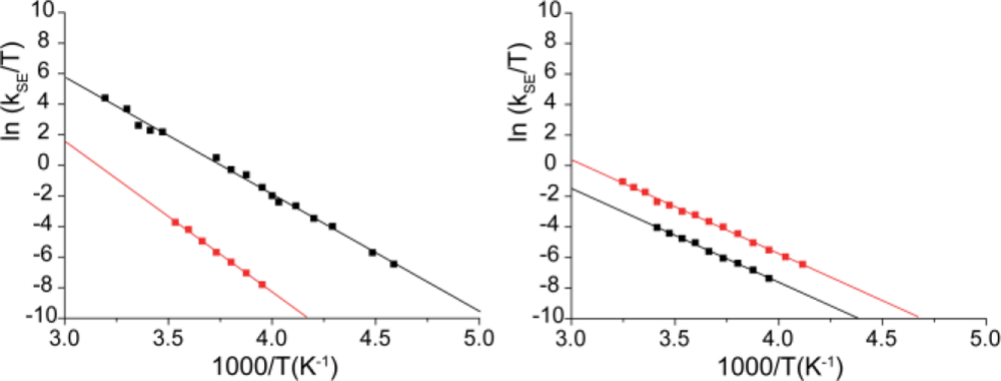
Eyring plots of ln(*k*_SE_/*T*) vs 1/*T* for complexes **3^+^_FF_** (left) and *4H^+^_MM_ (right). Data in C_7_D_8_ and C_6_D_5_Cl are shown in black and red, respectively.

Rate constants of the SE process (*k*_SE_, in s^–1^) can be measured by means of dynamic NMR methods, such as 2D magnetization transfer experiments (EXSY) and line shape analysis, by monitoring chemical exchange between magnetically inequivalent nuclei belonging to the two halves of the coordinated [ONN′O′] ligand.^70,71^ We studied complexes derived from **3** and **4** (or *4H) in C_7_D_8_ and C_6_D_5_Cl and *k*_SE_ values as a function of temperature and solvent are summarized in Tables S2 and S3 (Supporting Information); the activation parameters, obtained from Eyring analysis (see Figure 8) are reported in Table 4.

**Table 4:** Activation Parameters for Site Epimerization (SE) in Cationic Complexes Derived from **3**, **4**, and *4H

species	solvent	Δ*H*^‡^	Δ*S*^‡^	Δ*G*^‡^_(298)_	Δ*G*^‡^_(333)_
**3^+^_FF_**	C_7_D_8_	15.2	10.0	12.2	11.9
**3^+^_FF_**	C_6_D_5_Cl	19.6	14.8	15.2	14.7
**4^+^_MM_**	C_7_D_8_	13.6	–9.3	16.4	16.7
*4H^+^_MM_	C_7_D_8_	12.2	–13.5	16.2	16.7
*4H^+^_MM_	C_6_D_5_Cl	12.2	–9.8	15.1	15.5
*4H^+^_FF_	C_6_D_5_Cl	16.6	15.2	12.1	11.6

In C_7_D_8_, the activation enthalpy (Δ*H*^‡^) and entropy (Δ*S*^‡^) of the SE process for **3^+^_FF_** are found to be 15.2 kcal mol^–1^ and 10 cal mol^–1^ K^–1^, respectively (Figure 8, left). The small, but positive, Δ*S*^‡^ value confirms that **3^+^_FF_** has solvent molecules coordinated to the formally “vacant” coordination site and that SE occurs through a dissociative interchange mechanism, where de-coordination of coordinated toluene appears to be the rate-limiting step, followed by rapid benzyl migration from one side to the other and re-coordination of toluene.

For all investigated temperatures, the SE process of **3^+^_FF_** in C_6_D_5_Cl is much slower than in C_7_D_8_; for example, *k*_SE_ = 0.10 s^–1^ in C_6_D_5_Cl vs 59.9 s^–1^ in C_7_D_8_ at 253 K. Eyring analysis showed that the enthalpic contribution (Δ*H*^‡^ = 19.6 kcal mol^–1^) to the overall barrier is about 4.5 kcal mol^–1^ larger than in C_7_D_8_, whereas the Δ*S*^‡^ value (14.8 cal mol^–1^ K^–1^) is somewhat more positive than in C_7_D_8_ (Figure 8, left). This implies stronger C_6_D_5_Cl binding to the metal center, but based on the similar entropy contribution, a dissociative interchange mechanism is also operative here. The results mirror previous findings for *ansa*-metallocenes.

For temperatures higher than 253 K, isomers **4^+^_MM_** and *4H^+^_MM_ are prevalent in solution and the kinetics of the SE process are remarkably different from that of **3^+^_FF_** (Figure 8, right). Firstly, *k*_SE_ are much less sensitive to the nature of solvent: for instance, at 253 K, *k*_SE_ is equal to 0.08, 0.16, and 1.03 s^–1^ for **4^+^_MM_** in C_7_D_8_, *4H^+^_MM_ in C_7_D_8_, and *4H^+^_MM_ in C_6_D_5_Cl, respectively. Secondly, the more coordinating C_6_D_5_Cl accelerates the SE process with respect to C_7_D_8_. Finally, deconvolution of enthalpic and entropic contributions to the kinetic barrier reveals (i) similar and relatively small Δ*H*^‡^ values (13.6 kcal mol^–1^ for **4H^+^_MM_** in C_7_D_8_, 12.2 kcal mol^–1^ for *4H^+^_MM_ in C_7_D_8_ and 12.2 kcal mol^–1^ for *4H^+^_MM_ in C_6_D_5_Cl) and (ii) similar and negative Δ*S*^‡^ values (−9.3 cal mol^–1^ K^–1^ for **4H^+^_MM_** in C_7_D_8_, −13.5 cal mol^–1^K^–1^for *4H^+^_MM_ in C_7_D_8_ and −9.8 cal mol^–1^K^–1^for *4H^+^_MM_ in C_6_D_5_Cl). In contrast to **3^+^_FF_**, the negative values of Δ*S*^‡^ imply a different SE mechanism, associative in nature, occurs (see below). Importantly, despite MM complexes having no solvent coordinated, solvent evidently plays a role in the SE mechanism; however, the modality by which the solvent triggers SE for MM systems is less obvious.

**Figure 9:**
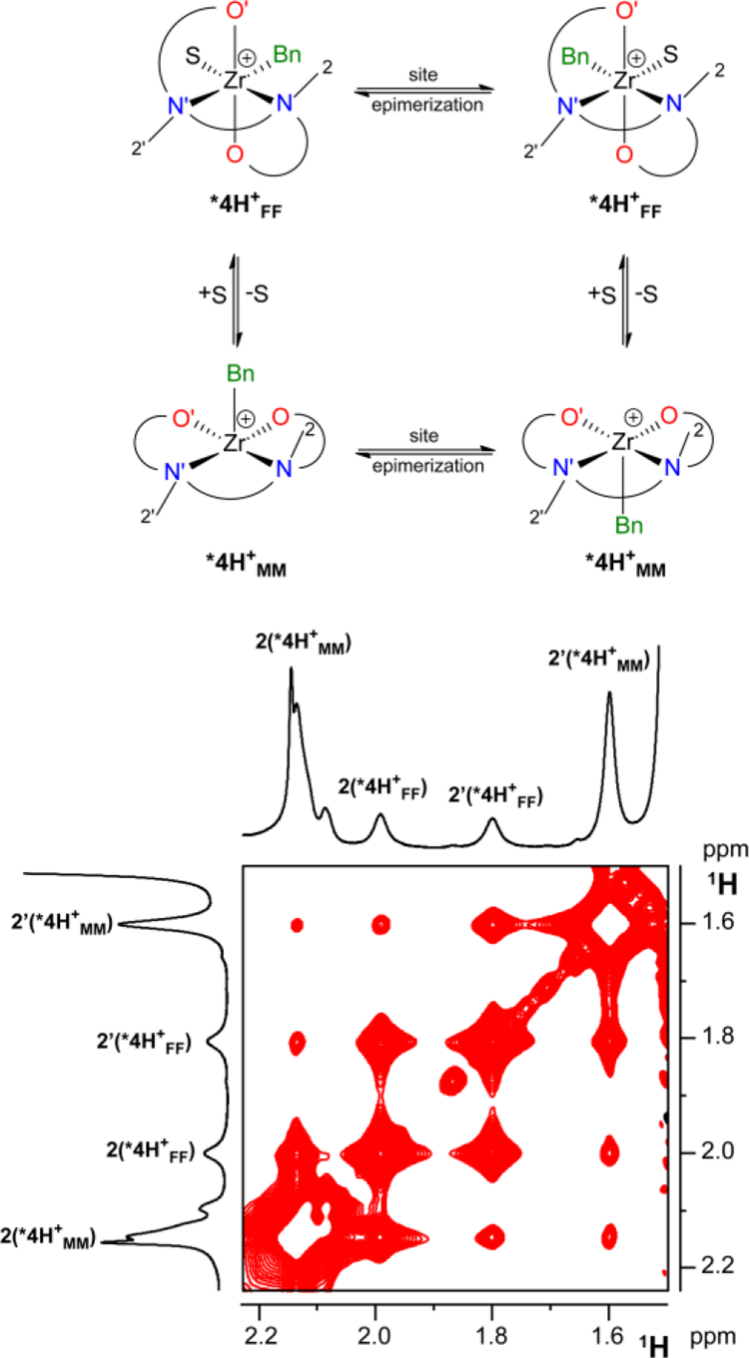
Section of the ^1^H EXSY NMR spectrum of a mixture of *4H^+^_MM_ and *4H^+^_FF_ (C_6_D_5_Cl, 243 K) showing the pattern of magnetization transfer relative to the interconversion between *4H^+^_MM_ and *4H^+^_FF_ as well as chemical exchange due to site epimerization within the same geometrical isomer.

Crucial information in this respect was obtained by further analyzing the equilibrium between *4H^+^_MM_ and *4H^+^_FF_ and the corresponding dynamic behavior in C_6_D_5_Cl at temperatures below 248 K. The presence of separated NMR resonances for Me^2^ and Me^2^′ moieties of both *4H^+^_MM_ and *4H^+^_FF_, in the 218–248 K temperature range, not only allowed to separately determine *k*_SE_ values for *4H^+^_MM_ and *4H^+^_FF_, but also allowed: (i) to experimentally determine the thermodynamic constant (*K*_eq_) for the equilibrium depicted in eq 1, by estimating the concentration of the two species using an external NMR reference, and (ii) to measure the rate constants at equilibrium for forward (*k*_1_) and backward (*k*_–1_) reactions by following the selective intermolecular magnetization transfer between corresponding Me^2^ (or Me^2^′) moieties of *4H^+^_MM_ and *4H^+^_FF_ (Figure 9). Experimental data are collected in Table 5.
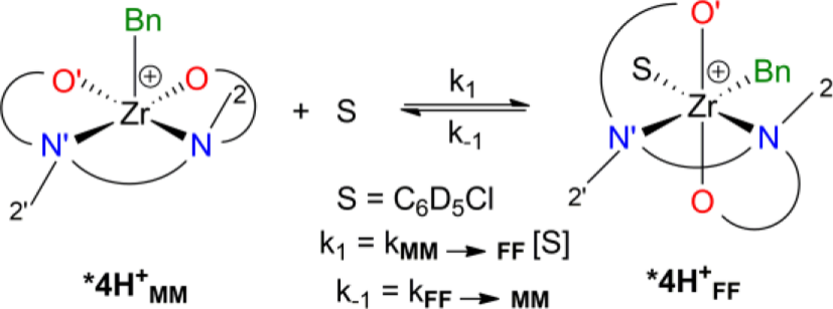
1

**Table 5:** Equilibrium Constant (*K*_eq_) Relative to Eq 1, Corresponding Rate Constants at Equilibrium (*k*_MM→FF_ and *k*_FF→MM_), Rate Constant of Site Epimerization Relative to *4H^+^_FF_ (*k*_SE_ (*4H^+^_FF_)) and Corresponding Thermodynamic Parameters

*T*	*K*_eq_ (M^–1^)	*k*_MM→FF_ (M^–1^ s^–1^)	*k*_FF→MM_ (s^–1^)	*k*_SE_ (*4H^+^_FF_) (s^–1^)
218	0.175			0.25
223	0.115			0.48
228		0.036	0.69	1.16
233	0.050	0.055	1.29	2.44
238	0.046	0.075	2.48	4.99
243	0.032	0.104	4.12	12.13
248		0.216	11.98	29.80
Δ*H*^0^ or Δ*H*^‡^	–7.1	9.0	14.9	16.6
Δ*S*^0^ or Δ*S*^‡^	–36	–25.1	6.5	15.2
Δ*G*^0^^a^ or Δ*G*^‡^^a^	1.4	14.9	13.4	13.1

a At 233 K.

**Figure 10:**
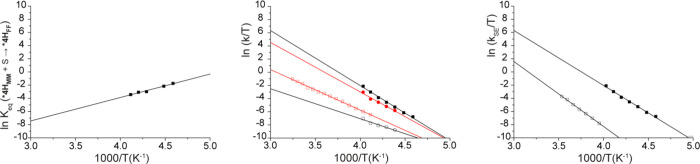
Left: Van’t Hoff plot of ln *K*_eq_ (*4H^+^_MM_ + S → *4H^+^_FF_) vs 1/*T* in C_6_D_5_Cl. Middle: Eyring plots, in C_6_D_5_Cl, of ln(*k*/*T*) vs 1/*T* for: *k*_MM→FF_ (empty black circles); *k*_FF→MM_ (filled red circles); *k*_SE_ for *4H^+^_MM_ (empty red squares); *k*_SE_ for *4H^+^_FF_ (filled black squares). Right: Eyring plots in C_6_D_5_Cl of ln(*k*_SE_/*T*) vs 1/*T* for **3^+^_FF_** (empty black squares) and *4H^+^_FF_ (filled black squares).

Concerning the equilibrium, Δ*H*^0^ and Δ*S*^0^ values were derived from the van’t Hoff plot of ln *K*_eq_ vs 1/*T* and found to be −7.1 kcal mol^–1^ and −36 cal mol^–1^ K^–1^, respectively, resulting in Δ*G*^0^_(233)_ = 1.4 kcal mol^–1^ (Figure 10, left). Interaction of the solvent with *4H^+^_MM_ is exothermic, adding evidence that *4H^+^_MM_ must be formulated as a pentacoordinate species and as expected, the reaction to form *4H^+^_FF_ is entropically disfavored.

As far as rate constants at equilibrium are concerned, the macroscopic, second order rate constant of the forward reaction, *k*_**MM**→**FF**_, computed by dividing the microscopic rate constant of magnetization transfer (*k*_1_) by the concentration of C_6_D_5_Cl, was found to be little dependent on temperature (Figure 10, middle), with Δ*H*^‡^, Δ*S*^‡^, and Δ*G*^‡^_(233)_ values of 9.0 kcal mol^–1^, −25.1 cal mol^–1^ K^–1^, and 14.9 kcal mol^–1^, respectively.

On the contrary, the rate constant for the unimolecular backward reaction, *k*_**FF**→**MM**_, is equal to the microscopic rate constant (*k*^–1^) and clearly increases with increasing temperature (Figure 10, middle). The corresponding activation parameters, Δ*H*^‡^ = 14.9 kcal mol^–1^, Δ*S*^‡^ = 6.5 cal mol^–1^ K^–1^, reflect the dissociative nature of the process, resulting in Δ*G*^‡^_(233)_ = 13.4 kcal mol^–1^.

Finally, *k*_SE_ values relative to *4H^+^_FF_ were much larger than those measured for *4H^+^_MM_, the major species present in solution at temperatures higher than 253 K (Figure 10, middle). Comparison of activation parameters (Δ*H*^‡^ = 16.6 kcal mol^–1^ and Δ*S*^‡^ = 15.2 cal mol^–1^ K^–1^ for *4H^+^_FF_; Δ*H*^‡^ = 12.2 kcal mol^–1^; Δ*S*^‡^ = −9.8 cal mol^–1^ K^–1^ for *4H^+^_MM_) confirms that the change in geometry is accompanied by an apparent change of the SE mechanism, involving specific interactions with the solvent molecule.

**Figure 11:**
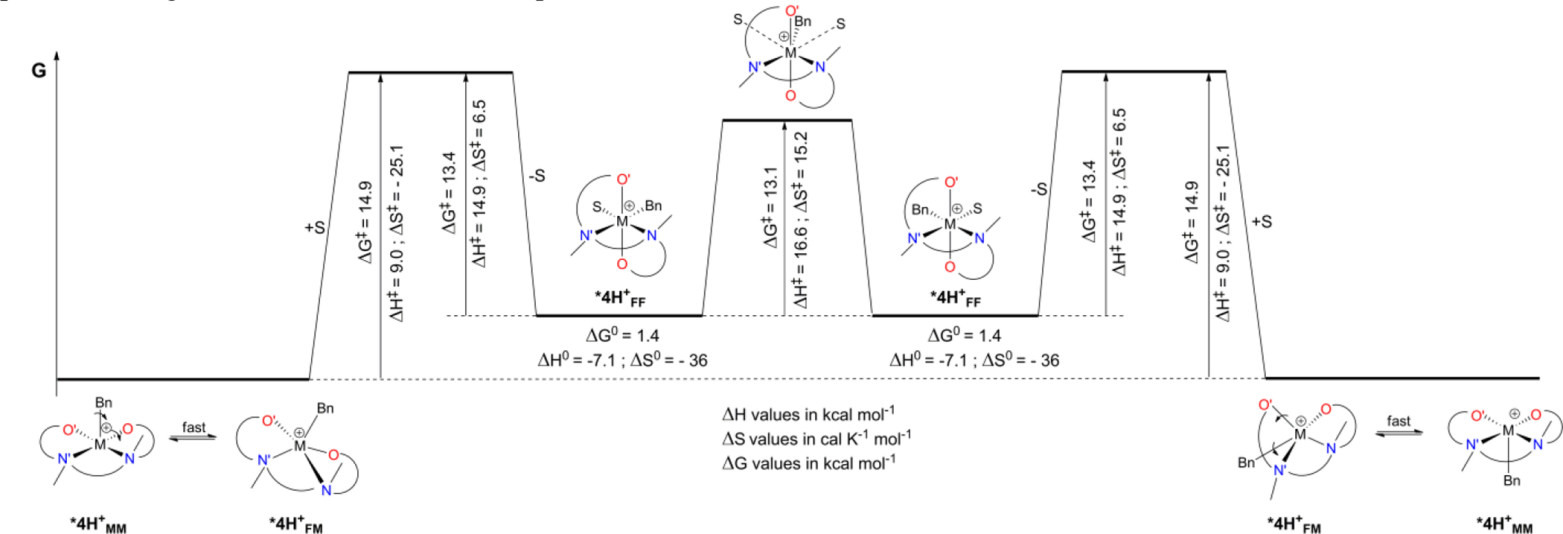
Experimentally-determined free energy profile vs reaction coordinates for the various processes involving *4H^+^. All Δ*G*^0^ and Δ*G*^‡^ values are computed at 233 K. Given the highly distorted nature of FM and MM isomers and their minimal energy differences, we assume a fast, barrierless or near barrierless FM/MM equilibrium.

Combining kinetic and thermodynamic experimental data in the temperature range 218–248 K allows to delineate an experimental semi-quantitative free energy profile (at 233 K) as depicted in Figure 11.

Under these conditions, the highest barrier is related to the conversion of *4H^+^_MM_ into the solvent-stabilized *4H^+^_FF_ isomer. Since coordination of the solvent *trans* to the benzyl moiety in the *4H^+^_MM_ isomer is unlikely to produce any intermediate relevant for the subsequent SE, it is reasonable to assume that solvent coordination occurs on the *fac*-*mer* isomer (*4H^+^_FM_). Whether there is a pre-equilibrium between *4H^+^_MM_ and *4H^+^_FM_, as softly supported by ^15^N chemical shifts and DFT computations (see above), or the *4H^+^_FM_ isomer is interacting with the solvent only in the transition state, is difficult to prove experimentally. Note that the *4H^+^_FF_ is thermodynamically less stable than the starting *4H^+^_MM_ isomer for entropic reasons. Once formed, the barrier of the SE process for the *4H^+^_FF_ isomer is indeed slightly lower than the barrier to convert it back to *4H^+^_MM_ by solvent dissociation. In other words, these data qualitatively confirm that the solvent triggers the apparent SE process of *4H^+^_MM_ by allowing the formation of the intermediate *4H^+^_FF_ isomer.

Quantification of this effect at higher temperature is strongly complicated by the interplay between the position of the thermodynamic equilibrium in eq 1 and coalescence of NMR resonances of *4H^+^_MM_ and *4H^+^_FF_ isomers. However, the experimental negative Δ*S*^‡^ values for the SE process between *4H^+^_MM_ diastereomers, as obtained from *k*_SE_ in the 243–308 K temperature range, still represent an indication that this process must be triggered by an associative step, i.e., some kind of interaction with the solvent molecules is needed.

**Figure 12:**
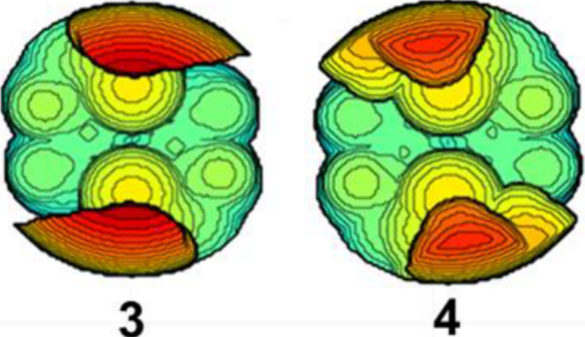
Topologic steric maps of the dichloride precursors show significantly more steric bulk close to the metal for catalyst **4** than for **3**, indicating steric interference between the ^*t*^Bu groups and solvent affects solvent coordination strength.

Dissecting the specific role of the phenolate substituents on the rate of the SE process is only possible in C_6_D_5_Cl at low temperature, where the behavior of **3^+^_FF_** and *4H^+^_FF_ can be compared. The corresponding Eyring plots of k_SE_ are shown in Figure 10, right. Clearly, *k*_SE_ for **3^+^_FF_**, bearing the aromatic anthracenyl substituent, is much slower than that of *4H^+^_FF_, having aliphatic substituents. The activation parameters suggest that the observed difference is mainly due to the enthalpic contribution to the activation barrier (Δ*H*^‡^ = 16.6 vs 19.6 kcal mol^–1^ for *4H^+^_FF_ and **3^+^_FF_**, respectively). Although electronic factors cannot be excluded, sterics seems to play a major role. With respect to the “flat” and aromatic anthracenyl substituent in **3**, which can adopt less sterically demanding orientations, the ^*t*^Bu groups in **4** shield the metal more effectively and thereby hinder the approach of solvent (Figure 12). The decreased solvent-metal interaction favors the (partial) release of the solvent molecule, which is necessary to trigger SE.

### Preliminary Studies of Salan Complexes under True Polymerization Conditions

The identification of an “easy” NMR indicator to determine the structure of Salan catalysts offered the unique opportunity to investigate the geometry adopted by the active species, i.e., polymeryl bearing complexes in the presence of a 1-alkene monomer that are resting states in olefin polymerization catalysis.^72^ Thus, reactions of *3^+^_FF_ and *4H^+^_MM_ with 1-hexene were monitored at low temperatures in C_6_D_5_Cl by NMR spectroscopy.

**Figure 13:**
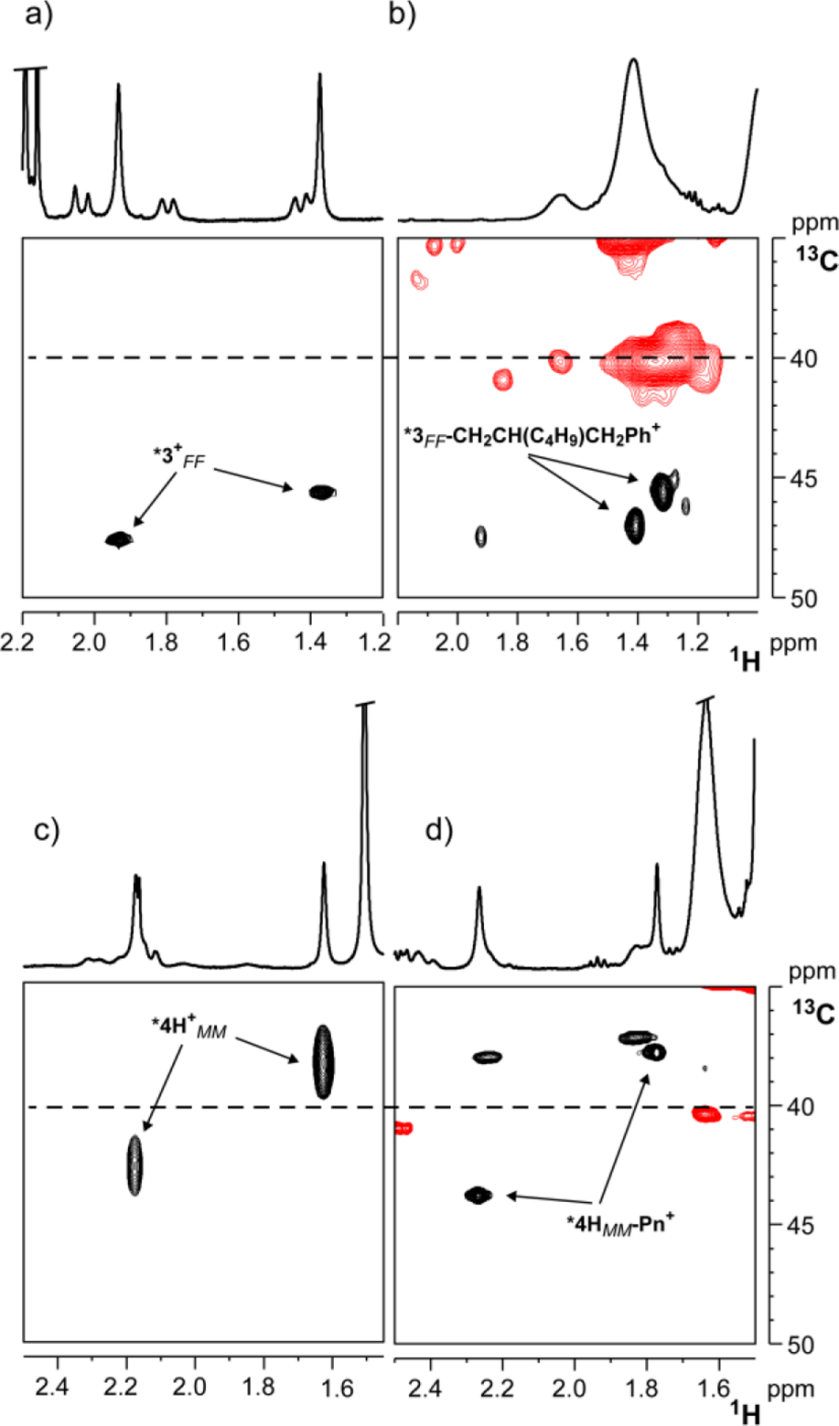
NMe regions in the multiplicity-edited ^1^H, ^13^C HSQC NMR experiments (black cross peaks = CH or CH_3_ moieties, red cross peaks = CH_2_ moieties) of: (a) *3^+^_FF_ (233 K); (b) *3_FF_-CH_2_-CH(C_4_H_9_)CH_2_Ph^+^ (233 K); (c) *4H^+^_MM_ (253 K); (d) *4H-P_n_^+^_MM_.

Addition of ∼30 equiv of 1-hexene to *3^+^_FF_ at 233 K resulted in the immediate consumption of the starting material with the concomitant formation of a main species, displaying ^13^C NMR chemical shifts of both NMe moieties above 40 ppm (45.6 and 47.0 ppm, respectively; Figure 13a,b). NMR characterization of such species points to the formation of *3_FF_-CH_2_-CH(C_4_H_9_)CH_2_Ph^+^, the product of the first insertion of 1-hexene into the Zr–benzyl bond, that maintains the polymerization active *fac-fac* geometry observed for the starting cationic species *3^+^_FF_ (see Supporting Information). Noteworthy, no NMe carbon resonances below 40 ppm could be detected. Minor species having the NMe carbons resonating above 40 ppm can instead be observed. Although their characterization is hampered by their very low concentration in solution, we speculate that they can arise from zirconium polymeryl species, *3_FF_-Pn^+^, retaining the *fac-fac* geometry.

The reaction of 1-hexene with *4H^+^_MM_ at 253 K (at this temperature, the equilibrium between *4H^+^_MM_ and *4H^+^_FF_ is strongly shifted to the left) proceeds much slower than in the case of *3^+^_FF_. After several minutes *4H^+^_MM_ is almost completely consumed and the new species dominating in solution features NMe carbon resonances at 37.7 and 43.8 ppm (Figure 13c,d), leading us to formulate it as the Zr-oligomeryl species, *4H_MM_-P_n_^+^. This insertion product, like the starting benzyl derivative, prefers the polymerization inactive *mer-mer* geometry (see Supporting Information for additional NMR data supporting this assignment).

## conclusions

The productivities of group 4-Salan olefin polymerization catalysts are strongly influenced by the nature of the ligand substituents, which appear to control the conformational pre-equilibrium between the active (*fac-fac*) and inactive (*mer-mer* or *fac-mer*) isomers of the cationic species. Understanding the factors that regulate those equilibria, and then which isomer dominates catalyst speciation, is experimentally difficult, especially in the presence of monomer, i.e., under catalytic conditions. Detailed in situ NMR studies of cationic benzyl species representative of fast (**3^+^**, *o*-anthracenyl) and slow (**4^+^**, *o*-^*t*^Bu) Salan catalysts allowed to dissect the intricate interplay between the nature of substituents and solvent on their structure and dynamic behavior in solution.

Cation **3^+^**, bearing the aromatic substituent, adopts the six-coordinate *fac-fac* geometry both in C_7_D_8_ (low polar and weakly coordinating) and C_6_D_5_Cl (slightly more polar and moderately coordinating). A solvent molecule is coordinated *cis* to the benzyl ligand as further demonstrated by single crystal X-ray analysis. Consistently, the SE process mirrors those observed for typical (and more rigid) metallocene cations, i.e., it has a dissociative nature and the barrier is higher in C_6_D_5_Cl due to the higher enthalpic cost for loosening cation-solvent interactions. For temperatures above 253 K, cation **4^+^**, bearing the aliphatic substituent, adopts the five-coordinated *mer-mer* (or *fac-mer*) geometry with no solvent coordination. Consistently, SE minimally depends on the nature of solvent; noteworthy, the barrier is characterized by a negative entropic contribution, suggesting an associative nature of the process. Thermodynamic and kinetic studies of the **4^+^_MM_** + S ⇋ **4H^+^_FF_** equilibrium in C_6_D_5_Cl (218–243 K) allowed to experimentally demonstrate that coordination of solvent to **4^+^_MM_** is rate determining along the pathways of SE of **4^+^_MM_**, offering a rationale for its associative nature.

Detailed analysis of NMR data in combination with DFT computations allowed to individuate diagnostic, and easily accessible, NMR parameters (^13^C of NMe moieties) capable of discriminating between *fac-fac* and *mer-mer* (or *fac-mer*) isomers. On one hand, this allowed to experimentally determine the prevalent species present in solution for benzyl cations bearing different substituents; the results correlate nicely with catalytic productivities. Salans bearing large but flat substituents (such as **3**) lead to fast catalysis, not limited by the presence of MM species. More spherical substituents cause a thermodynamic preference for MM geometries and the resulting low concentration of FF active species leads to slow catalysis (as for **4**). Salan **7**, with its sp^3^ substituent atom but large and flat “body”, is probably a borderline case.

More importantly, these detailed NMR studies allowed, for the first time, to determine the geometry of active *polymeryl* species in the presence of a large excess of monomer. Similar to the starting benzyl cation, “slow” **4-polymeryl^+^** is observed in its inactive *mer-mer* resting state, as previously suggested by DFT. On the contrary, the “fast” **3^+^** cation is initially converted to **3_FF_-CH_2_-CH(C_4_H_9_)CH_2_Ph^+^**, the product of the first 1-hexene insertion, observed in its FF geometry. No signature for species having the *mer-mer* (or *fac-mer*) ligand arrangement could be detected in solution, suggesting that also the propagating species, **3_FF_-P_n_^+^**, prefer a *fac-fac* geometry.

## Experimental Section

### General Considerations

All manipulations and synthesis of air- and moisture sensitive chemicals were performed under rigorous exclusion of oxygen and moisture in flame-dried Schlenk-type glassware interfaced to a high vacuum line (10^–5^ Torr), or in a nitrogen-filled MBraun glove-box (<0.5 ppm O_2_ and H_2_O). Hydrocarbon solvents used for synthesis were dried over 4 Å molecular sieves and degassed by bubbling with dry argon. Ethereal solvents used for synthesis were distilled from sodium/benzophenone. Molecular sieves (4 Å, MS) were activated for 24 h at ca. 200–230 °C under dynamic vacuum. All solvents used in the studies of the activated complexes were freeze-pump-thaw degassed on the high vacuum line, dried over the appropriate drying agent (Na/K alloy for benzene, pentane, toluene, benzene-d_6_ and toluene-d_8_; CaH_2_ for chlorobenzene-d_5_, 1,2-difluorobenzene) and vacuum transferred into dry storage Schlenk flasks equipped with PTFE valves. [CPh_3_][B(C_6_F_5_)_4_] was obtained from Boulder Scientific Company and used as received. TiBA (tri-isobutyl aluminum) was purchased from Sigma-Aldrich and used as received. High-resolution mass spectra (HRMS) were recorded on an Agilent Technologies 6530 Q-TOF LC/MS system paired with Agilent 1260 HPLC and using Agilent JetStream or APCI ion source. Ethene (Linde, 99.95%) and propene (Rivoira, 99.6%) were purified by flowing them through a column containing activated 4 Å molecular sieves and an activated Cu catalyst (BASF R0-11G). 1-Hexene (Sigma-Aldrich, 99%) was purified by passing it through a mixed-bed column of the activated Cu catalyst and 4 Å molecular sieves. Toluene (Romil) was dried using an MBraun SPS-5 solvent purification unit. 1,2-Dichlorobenzene (Romil, >99.8% isomeric purity) was used as received.

### NMR Spectroscopy Experiments

All samples for NMR measurements were prepared inside the glove-box; flame-dried NMR tubes equipped with a PTFE valve (J-Young NMR tubes) were used. One- and two-dimensional, homo- and hetero-nuclear NMR spectra were recorded on a Bruker Avance III 400 spectrometer equipped with a smartprobe and using standard pulse sequences. Unless otherwise stated, referencing is relative to external TMS (^1^H and ^13^C), NH_3_ (^15^N) and CCl_3_F (^19^F). Variable temperature ^1^H EXSY NMR measurements were acquired by using the PFG version of the NOESY sequence (noesygptp), setting a relaxation delay of 1 s, with mixing time values (τ_m_) ranging between 2.7 and 800 ms depending on the rate of chemical exchange. Typically, a matrix of 1024 × 1024 data points was used for acquisition and the raw data were processed using zero-filling to 2048 data points in both spectral dimensions. The spectral window and the number of transients were optimized depending on distribution, relevant resonances, and sample concentration. Typically, at least two experiments with different τ_m_ values were acquired for each temperature, and the rate constant values were obtained from the average of all the values. For the IPS dynamical motions, rate constants (*k*_IPS_, s^–1^) were evaluated by the method proposed by Perrin and Dwyer,^70^ and were calculated from the integration of the 2D spectra by using the EXSYCALC software.^73^ In some cases, exchange rate constants were also estimated at higher temperatures, where exchanging resonances first coalesce and then narrow, using methods of lineshape analysis and standard equations for two-sites exchange in the absence of scalar coupling.^71^ Activation parameters of dynamical motions were estimated from the corresponding Eyring plots; errors on activation enthalpy and activation entropy were determined from the quality of linear fitting and computed at 95% confidence interval. For the ^15^N NMR experiments, standard sequences provided by Bruker were employed, in particular, the zgig sequence for 1D ^15^N NMR spectra, and the hsqcgpph sequence for 2D ^15^N NMR, with an optimization of the 90° pulse at 21 μs at 74 PLW1, with a long-range constant of 7 Hz. To describe the multiplicity of the signals, the following abbreviations are used: s, singlet; bs, broad singlet; d, doublet; bd, broad doublet; dd, doublet of doublets; t, triplet; and m, multiplet.

### Synthetic Procedures

Complexes **1** and **3**,^38^**2** and **7**,^39^**5**,^74^**4** and **6**,^46^ 3-(*tert*-butyl)-2-hydroxybenzaldehyde,^75^ and 3-(anthracen-9-yl)-2-(methoxymethoxy)-5-methylbenzaldehyde^76^ were prepared according to the published procedures. Ethylenediamine-^15^N_2_ dihydrochloride (98% atom 15 N, 99% (CP), CAS: 84050-98-6) was purchased from Aldrich. All other reagents were purchased from commercial sources and used as received.

### Polymerization Experiments and Polymer Characterization

Polymerization experiments were performed at 60 °C and propene partial pressure of 6.6 bar in a high-throughput experimentation setup (PPR48, fully contained in a glove-box), according to protocols reported in refs (40−45). In all cases, TiBA was used as a scavenger and [CPh_3_][B(C_6_F_5_)_4_] (TTB) as an activator.

All polymers were characterized by means of high-temperature gel permeation chromatography (GPC) and ^13^C NMR spectroscopy. GPC curves were recorded with a Freeslate Rapid GPC setup, equipped with a set of two mixed-bed Agilent PLgel 10 μm columns and a Polymer Char IR4 detector. Calibration was performed with the universal method, using 10 monodisperse polystyrene samples (*M*_n_ between 1.3 and 3700 kDa).

Quantitative ^13^C NMR spectra were recorded using a Bruker Avance III 400 spectrometer equipped with a high-temperature cryoprobe for 5 mm OD tubes, on 45 mg mL^–1^ polymer solutions in tetrachloroethane-d_2_ (with BHT added as a stabilizer, [BHT] = 0.4 mg mL^–1^). Acquisition conditions were: 45° pulse; acquisition time, 2.7 s; relaxation delay, 2.0 s; 2 K transients. Broad-band proton decoupling was achieved with a modified WALTZ16 sequence (BI_WALTZ16_32 by Bruker).

### DFT Calculations

Geometries of dichloride precursor complexes and activated complexes with and without solvent were fully optimized using the Gaussian 16 software package.^55^ The BOpt software package was employed for data collection.^58^ Following the protocol proposed in ref (59), the TPSSh^60^/cc-pVDZ(-PP)^61,62,77^ level of theory, using a small core pseudo potential on Zr,^64,65^ was employed for optimization. This protocol has been successfully used, in combination with TPSSh-D_zero_/cc-pVTZ(-PP) single-point energy corrections, to address polymerization related problems in post-metallocene chemistry.^78,79^ The density fitting approximation (Resolution of Identity, RI)^80−83^ and standard Gaussian16 quality settings [Scf = Tight and Int(Grid = UltraFine)] were used at the optimization stage and for single-point energy (SP) calculations. All structures represent true minima (as indicated by the absence of imaginary frequencies). Grimme’s dispersion correction^84^ with zero damping and the polarizable continuum model (PCM)^85^ for solvent corrections were employed at the SP stage. Final energies were then combined with thermal corrections (enthalpy and entropy, 298 K, 1 bar) to obtain final free energies; entropy corrections were scaled by 0.67 to account for reduced freedom of movement in solution.^86−88^ Buried volume descriptors were calculated using the SambVca 2.1 program.^89^ The CREST program^54^ was used to sample the conformational space and generate guess structures for full DFT optimization.

Further information about the synthesis of the ligands and the complexes, NMR spectra, polymerizations, and DFT calculations is reported in the Supporting Information section.

## Supporting Information Available

General experimental procedures; X-ray data for ***3^+^_FF_·C_7_D_8_** and ***3^+^_FF_·C_6_D_5_Cl**, computational details and results; NMR characterizations; experimental SE rate constants as a function of temperature (PDF)
Combined coordinate file for optimized DFT structures (ZIP)
