# Supplementary material for: Octahedral Zirconium Salan Catalysts for Olefin Polymerization: Substituent and Solvent Effects on Structure and Dynamics
Source: Inorg Chem. 2023 Sep 19;62(39):16021–37. doi: 10.1021/acs.inorgchem.3c02153 (PMC10548417; doi:10.1021/acs.inorgchem.3c02153)
Supplement: Supplementary file 1 — ic3c02153_si_001.pdf [file ic3c02153_si_001.pdf]

Supporting Information

Octahedral zirconium Salan catalysts for olefin polymerization: substituent and solvent effects on structure and dynamics

Anna Dall'Anese,[a,d] *Pavel S. Kulyabin,*[b,d] *Dmitry V. Uborsky,*[b,d,*] *Antonio Vittoria,*[c,d] *Christian Ehm,*[c,d,*] *Roberta Cipullo,*[a,d] *Peter H. M. Budzelaar,*[c,d] *Alexander Z. Voskoboynikov,*[b,d] *Vincenzo Busico,*[c,d] *Leonardo Tensi,*[e] *Alceo Macchioni,*[a,d] *Cristiano Zuccaccia.*[a,d,*]

[a] Dipartimento di Chimica, Biologia e Biotecnologie, Università degli Studi di Perugia, Via dell'Elce di sotto 8, 06123 Perugia, Italy
[b] Department of Chemistry, Lomonosov Moscow State University, 1/3 Leninskie Gory, 119991 Moscow, Russia
[c] Dipartimento di Scienze Chimiche, Università di Napoli Federico II, Via Cintia, 80126 Napoli, Italy
[d] DPI, P.O. Box 902, 5600 AX Eindhoven, the Netherlands
[e] Dipartimento di Scienze Farmaceutiche, Università degli Studi di Perugia, Via del Liceo 1, 06123 Perugia, Italy

Email: cristiano.zuccaccia@unipg.it; christian.ehm@unina.it; duborsky@med.chem.msu.ru.

Table of content

Experimental section..S2
- **General considerations**..S2
- **NMR Spectroscopy Experiments**..S2
- **Synthetic procedures**..S3
- **Activation of Salan complexes**..S19
- **NMR spectra of activated complexes**..S26
- **Reactivity of $*3^{+}_{FF}$ with 1-hexene**..S32
- **Reactivity of $*4H^{+}_{MM}$ with 1-hexene**..S36
- **Table S1 (^{13}C NMR data of C2 and C2')**..S41
- **Table S2 (Site Epimerization rate constants in C_7D_8)**..S41
- **Table S3 (Site Epimerization rate constants in C_5D_5Cl)**..S42
- **X-Ray crystallography**..S43
- **Conformer Sampling and DFT NMR predictions**..S46
- **Additional Polymerization Details**..S51
- **References**..S51

Experimental Section

General considerations

All manipulations and synthesis of air- and moisture sensitive chemicals were performed under rigorous exclusion of oxygen and moisture in flame-dried Schlenk-type glassware interfaced to a high-vacuum line (10^{-5} Torr), or in a nitrogen-filled MBraun glovebox (<0.5 ppm O_2 and H_2O). Hydrocarbon solvents used for synthesis were dried over 4Å molecular sieves and degassed by bubbling with dry argon. Ethereal solvents used for synthesis were distilled from sodium/benzophenone. Molecular sieves (4 Å, MS) were activated for 24 h at ca. 200−230 °C under dynamic vacuum. All solvents used in the studies of the activated complexes were freeze-pump-thaw degassed on the high vacuum line, dried over the appropriate drying agent (Na/K alloy for benzene, pentane, toluene, benzene-d_6 and toluene-d_8; CaH_2 for chlorobenzene-d_5, 1,2-difluorobenzene) and vacuum transferred into dry storage Schlenk flasks equipped with PTFE valves. $[CPh_3][B(C_6F_5)_4]$ was obtained from Boulder Scientific Company and used as received. TiBA (tri-isobutyl aluminum) was purchased from Sigma-Aldrich and used as received. High-resolution mass spectra (HRMS) were recorded on an Agilent Technologies 6530 Q-TOF LC/MS system paired Agilent 1260 HPLC and using Agilent JetStream or APCI ion source. Ethene (Linde, 99.95%) and propene (Rivoira, 99.6%) were purified by flowing them through a column containing activated 4 Å molecular sieves and an activated Cu catalyst (BASF R0-11G). 1-Hexene (Sigma-Aldrich, 99%) was purified by passing it through a mixed-bed column of the activated Cu catalyst and 4 Å molecular sieves. Toluene (Romil) was dried using an MBraun SPS-5 solvent purification unit. 1,2-Dichlorobenzene (Romil, >99.8% isomeric purity) was used as received.

NMR Spectroscopy Experiments

All samples for NMR measurements were prepared inside the glove box; flame-dried NMR tubes equipped with a PTFE valve (J-Young NMR tubes) were used. One- and two-dimensional, homo- and hetero-nuclear NMR spectra were recorded on a Bruker Avance III 400 spectrometer equipped with a smartprobe and using standard pulse sequences. Unless otherwise stated, referencing is relative to external TMS (^1H and ^{13}C), NH_3 (^{15}N) and CCl_3F (^{19}F). Variable temperature ^1H EXSY NMR measurements were acquired by using the PFG version of the NOESY sequence (noesygptp), setting a relaxation delay of 1 s, with mixing time values (τ_m) ranging between 2.7 and 800 ms depending on the rate of chemical exchange. Typically, a matrix of 1024x1024 data points was used for acquisition and the raw data were processed using zero-filling to 2048 data points in both spectral dimensions. The spectral window and the number of transients were optimized depending on distribution and of relevant resonances and the sample concentration. Typically, at least two experiments with different

τ_m values were acquired for each temperature, and the rate constant values were obtained from the average of all the values. For the Ion Pair Symmetrization (IPS) dynamical motions, rate constants (k_{IPS}, s^{-1}) were evaluated by the method proposed by Perrin,[1] and were calculated from the integration of the 2D spectra by using the EXSYCALC software.[2] In some cases, exchange rate constants were also estimated at higher temperature, where exchanging resonances first coalesce and then narrow, using methods of lineshape analysis and standard equations for two-sites exchange in the absence of scalar coupling.[3] Activation parameters of dynamical motions were estimated from the corresponding Eyring plots; errors on activation enthalpy and activation entropy were determined from the quality of linear fitting and computed at 95% confidence interval. For the ^{15}N NMR experiments, standard sequences provided by Bruker were employed, in particular the zgig sequence for 1D ^{15}N NMR spectra, and the hsqcgpph sequence for 2D ^{15}N NMR, with an optimization of the 90° pulse at 21 μs at 74 PLW1, with a long-range constant of 7 Hz.

To describe the multiplicity of the signals, the following abbreviations are used: s, singlet; bs, broad singlet; d, doublet; bd, broad doublet; dd, doublet of doublets; t, triplet; and m, multiplet.

Synthetic procedures

Complexes **1** and **3**,[4] **2** and **7**,[5] **5**,[6] **4** and **6**, 3-(*tert*-butyl)-2-hydroxybenzaldehyde,[7] 3-(anthracen-9-yl)-2-(methoxymethoxy)-5-methylbenzaldehyde,[8] and 2-(2,2-diphenylethyl)-4-methylphenol[10], were prepared according to the published procedures. Ethylenediamine-$^{15}N_2$ dihydrochloride (98% atom 15N, 99% (CP), CAS: 84050-98-6) was purchased from Aldrich. All other reagents were purchased from commercial sources and used as received.

Synthesis of *4H

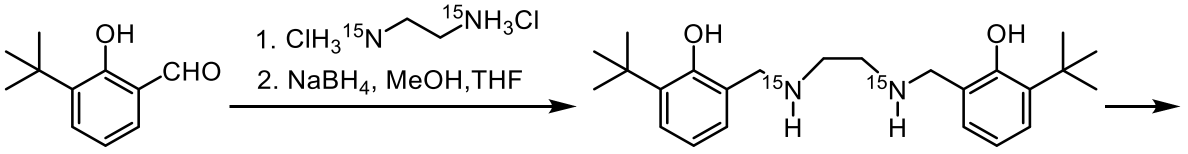

L*4H-H

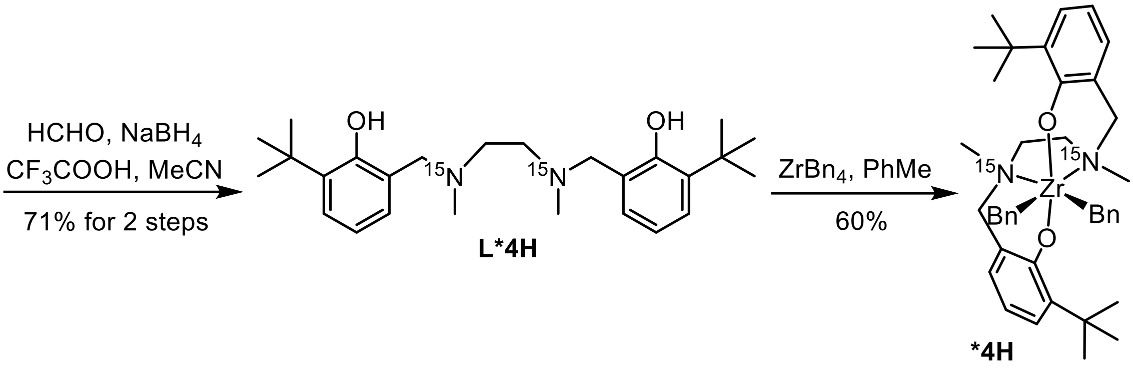

Synthesis of ***L*4H-H***

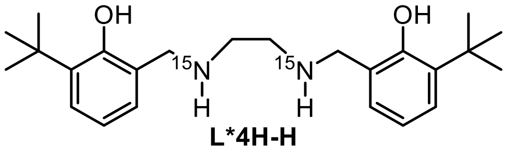

L*4H-H

A mixture of ethylenediamine-$^{15}N_2$ dichloride (50.5 mg, 374 μmol) and ethylenediamine dichloride (151.5 mg, 1.14 mmol) was suspended in 10 mL of dry methanol, and then trimethylamine (422 μL, 3.0 mmol) was added. The resulting mixture was stirred until all the solid was dissolved. The solution was added to the mixture of 3-(*tert*-butyl)-2-hydroxybenzaldehyde (539.2 mg, 3.0 mmol) and 12 mL of dry methanol. The mixture was stirred at room temperature for 2 h, and yellow precipitate was formed. Next, 15mL of dry THF was added to dissolve the precipitate, and the obtained solution was cooled to 0°C with ice bath. After that, sodium tetraborohydride (1.5 g, 39.5 mmol) was added to the solution slowly. The resulting mixture was stirred at room temperature for another 30 min, then poured in 100 mL of water and extracted with dichloromethane (3×30 mL). The combined organic layers were dried over anhydrous Na_2SO_4, and the solvents were evaporated. The residue was sufficiently pure **L*4H** (571 mg, 99% yield, approx. 25% ^{15}N) as a white solid. ^1H NMR (400 MHz, CD_2Cl_2): δ 7.16 (dd, J_1 = 7.9 Hz, J_2 = 1.6 Hz, 2H), 6.86 (m, 2H), 6.69 (m, 2H), 3.97 (s, 4H), 2.82 (s, 4H), 1.38 (s, 18H). ^{13}C NMR (101 MHz, CD_2Cl_2): δ 157.5, 137.0, 127.0, 126.3, 123.1, 118.6, 53.2, 48.1, 34.9, 29.6. HRMS (APCI) m/z calcd. for $C_{24}H_{37}{}^{14}N_2O_2^+$ $[M+H]^+$ and for $C_{14}H_{37}{}^{15}N_2O_2^+$ $[M+H]^+$: 385.2850 and 387.2790; found: 385.2855 and 387.2789 (relative intensity ≈ 2.7/1.0).

Synthesis of ***L*4H***

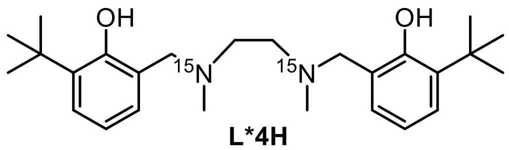

L*4H

A suspension of **L*4H-H** (530 mg, 1.38 mmol) in 24 mL of dry acetonitrile was treated with 3.2 mL of trifluroacetic acid which resulted in homogenization of the mixture. Next, formaldehyde solution, (formalin, 1.1 mL, 14.3 mmol, 37% CH_2O), was added, and the mixture was cooled to 0°C with ice bath. After that, sodium tetraborohydride (1.5 g, 39.5 mmol) was added to the solution at 0°C slowly. The resulting mixture was stirred at room temperature overnight. Then, 50 mL of saturated aqueous $NaHCO_3$ was added, and the mixture was extracted with dichloromethane (3×30 mL). The combined organic layers were dried over anhydrous Na_2SO_4, and the solvents were evaporated. The residue was dissolved in a mixture of 8 mL of dichloromethane and 30 mL of methanol. Then, dichloromethane was rotary evaporated which resulted in precipitation. The precipitate was filtered off, washed twice with 10 mL of methanol, and dried in vacuum. This procedure gave pure **L*4H** (442 mg, 71% yield, approx. 25% ^{15}N) as a white solid. ^{1}H NMR (400 MHz, CD_2Cl_2): δ 10.95 (br.s, 2H), 7.17 (dd, J_1 = 7.8 Hz, J_2 = 1.5 Hz, 2H), 6.84 (dd, J_1 = 7.3 Hz, J_2 = 1.5 Hz, 2H), 6.70 (m, 2H), 3.68 (s, 4H), 2.62 (br.s, 4H), 2.23 (s, 6H), 1.39 (s, 18H). ^{13}C NMR (101 MHz, CD_2Cl_2): δ 157.3, 136.8, 127.1, 126.3, 122.5, 118.6, 62.6, 53.8, 41.5, 34.9, 29.6. HRMS (APCI) m/z calcd. for $C_{26}H_{41}{}^{14}N_2O_2^+$ [M+H]$^+$ and for $C_{26}H_{41}{}^{15}N_2O_2^+$ [M+H]$^+$: 413.3163 and 415.3103; found: 413.3165 and 415.3095 (relative intensity ≈ 2.7/1.0).

Synthesis of ***4H**

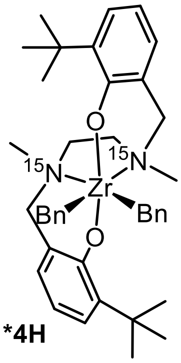

***4H**

In a glovebox with argon atmosphere, ligand **L*4H** (335 mg, 812 µmol) was dissolved in warm toluene (30 mL), and a solution of $ZrBn_4$ (370 mg, 812 µmol) in toluene (5 mL) was added. The mixture was stirred at r.t. overnight. Then, the solution was concentrated in vacuum, the precipitated solid was filtered off, washed with 2 mL of toluene and 5 mL of pentane to give the product as a white solid (335 mg, 60 % yield, approx. 25% ^{15}N). ^{1}H NMR (400 MHz, CD_2Cl_2): δ 7.33 (d, J = 7.2 Hz, 2H), 6.88–6.94 (m, 8H), 6.80 (m, 2H), 6.74 (m, 2H), 6.66 (m, 2H), 3.74 (d, J = 13.7 Hz, 2H), 3.01 (m, 2H), 2.85 (d, J = 13.7 Hz, 2H), 2.60 (d, J = 10.4 Hz, 2H), 2.20 (s, 6H), 2.10 (d, J = 10. 4 Hz, 2H), 1.74 (m, 2H), 1.63 (s, 18H). ^{13}C NMR (101 MHz,

CD_2Cl_2): δ 159.2, 148.8, 138.2, 128.7, 128.4, 127.6, 126.7, 126.2, 121.0, 119.2, 68.9, 63.8, 53.2, 46.2, 35.4, 30.3.

Synthesis of *3

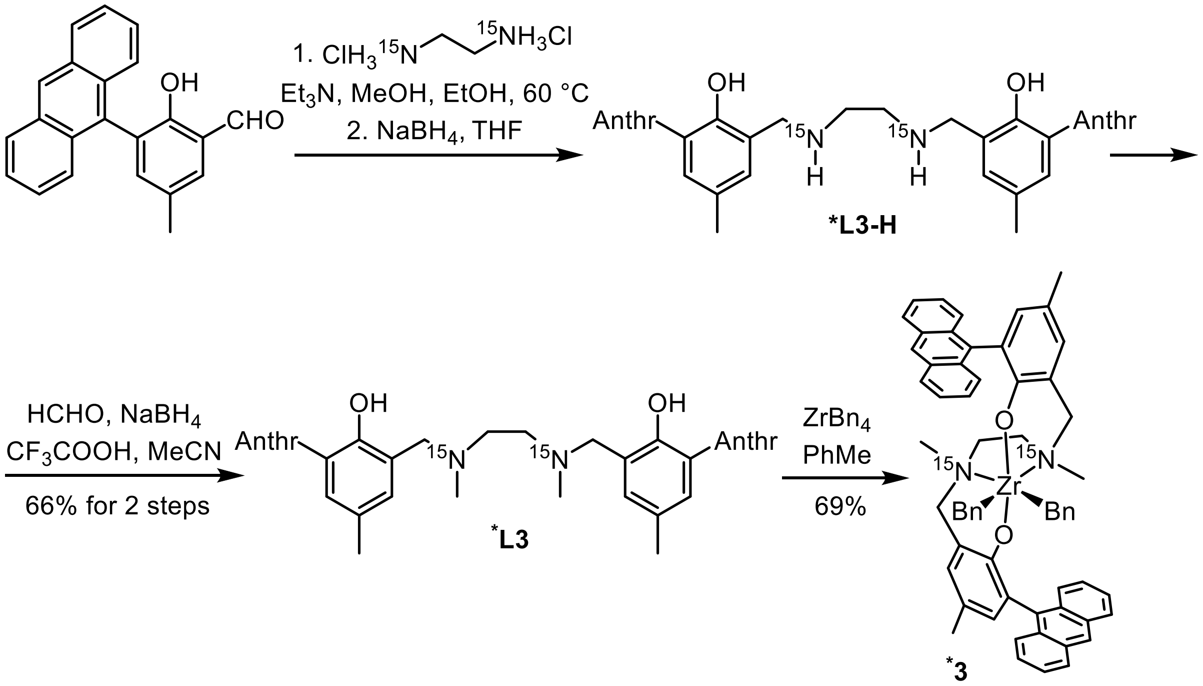

Synthesis of 3-(anthracen-9-yl)-2-hydroxy-5-methylbenzaldehyde

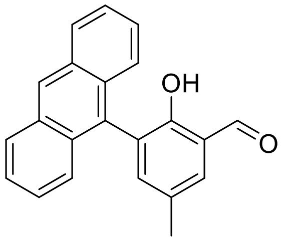

3-(Anthracen-9-yl)-2-(methoxymethoxy)-5-methylbenzaldehyde (5.2 g, 14.6 mmol) was dissolved in 100 mL of THF and 100 mL of methanol following by addidtion of 1 mL of conc. HCl. The resulting solution was stirred at 60°C overnight. Next, volatiles were evaporated and the residue was suspended in 300 mL of water following by extraction with dichloromethane (3×50 mL). The combined organic layers were dried over anhydrous Na_2SO_4, and the solvents were evaporated giving pure product as a yellowish solid (4.4 g, 97% yield). ^{1}H NMR (400 MHz, $CDCl_3$): δ 10.95 (s, 1H), 10.02 (s, 1H), 8.53 (s, 1H), 8.05 (d, J = 8.5 Hz, 2H), 7.58 (m, 3H), 7.46 (m, 2H), 7.39 (m, 3H), 2.44 (s, 3H). ^{13}C NMR (101 MHz, $CDCl_3$): δ 196.7, 157.7, 141.1, 133.6, 131.4, 130.9, 130.2, 129.1, 128.6, 127.3, 127.2, 126.1, 125.7, 125.1, 120.6, 20.3. HRMS (APCI) m/z calcd. for $C_{22}H_{17}O_2^+$ $[M+H]^+$: 313.1223 found: 313.1219.

*Synthesis of ***L3-H***

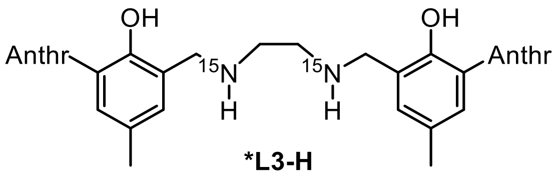

***L3-H**

A mixture of ethylenediamine-$^{15}N_2$ dichloride (51 mg, 378 µmol) and ethylenediamine dichloride (175 mg, 1.32 mmol) was suspended in 10 mL of dry methanol, and then trimethylamine (413 µL, 3.0 mmol) was added. The resulting mixture was stirred until all the solid was dissolved. In a separate flask, a suspension of 3-(anthracen-9-yl)-2-hydroxy-5-methylbenzaldehyde (1.06 g, 3.39 mmol) in a mixture of 45 mL of dry methanol and 45 mL of dry ethanol was heated until all the aldehyde was dissolved. Immediately after homogenization, a solution of the ethylenediamines was added to the hot aldehyde solution which resulted in formation of yellow precipitate. The mixture was stirred at room temperature for 2 h. Next, 210 mL of dry THF was added to dissolve the precipitate, and sodium tetraborohydride (455 mg, 12 mmol) was added to the solution slowly. The resulting mixture was stirred at room temperature for 1 h, and then the solvents were evaporated. The residue was suspended in 200 mL of water, and the crude product was extracted with dichloromethane (3×50 mL). The combined organic layers were dried over anhydrous Na_2SO_4, and the solvents were evaporated. The residue was sufficiently pure ***L3-H** (1019 mg, 92% yield, approx. 23% ^{15}N) as a white solid. ^{1}H NMR (400 MHz, $CDCl_3$): δ 8.47 (s, 2H), 8.02 (d, J = 8.5 Hz, 4H), 7.66 (d, J = 8.7 Hz, 4H), 7.42 (m, 4H), 7.31 (m, 4H), 6.95 (d, J =1.7 Hz, 2H), 6.82 (d, J =1.7 Hz, 2H), 3.91 (s, 4H), 2.70 (s, 4H), 2.27 (s, 6H). ^{13}C NMR (101 MHz, $CDCl_3$): δ 153.7, 133.7, 132.3, 131.5, 130.3, 129.0, 128.5, 128.0, 126.8, 126.5, 125.5, 125.3, 125.0, 122.1, 52.7, 47.9, 20.5. HRMS (APCI) m/z calcd. for $C_{46}H_{41}{}^{14}N_2O_2^+$ $[M+H]^+$ and for $C_{46}H_{41}{}^{15}N_2O_2^+$ $[M+H]^+$: 653.3163 and 655.3103; found: 653.3159 and 655.3092 (relative intensity ≈ 2.7/1.0).

Synthesis of ****L3***.

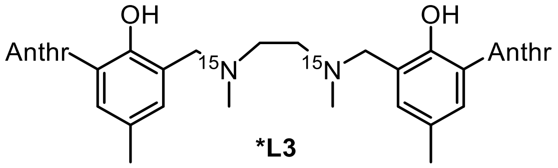

***L3**

A suspension of ***L3-H** (938 mg, 1.44 mmol) in 31 mL of dry acetonitrile was treated with 3.3 mL of trifluroacetic acid which resulted in homogenization of the mixture. Next, formaldehyde solution, formalin (1.1 mL, 14.3 mmol, 37% CH_2O), was added, and the mixture was cooled to 0°C with ice bath. After that, sodium tetraborohydride (1.04 g, 27 mmol) was added to the solution at 0°C slowly. The resulting mixture was stirred at room temperature overnight. Next,

the solvents were evaporated, the residue was suspended in 150 mL of saturated aqueous $NaHCO_3$, and the mixture was extracted with dichloromethane (3×50 mL). The combined organic layers were dried over anhydrous Na_2SO_4, and solvents were evaporated. The residue was dissolved in a mixture of 15 mL of dichloromethane and 45 mL of methanol. Then, dichloromethane was rotary evaporated to a reduced volume until precipitation started. The precipitate was filtered off, washed twice with 10 mL of methanol and dried in vacuum. This procedure gave pure ***L3** (678 mg, 69% yield, approx. 23% ^{15}N) as a white solid. ^1H NMR (400 MHz, $CDCl_3$): δ 10.64 (br.s, 2H), 8.46 (s, 2H), 8.02 (d, J = 8.6 Hz, 4H), 7.63 (d, J = 8.8 Hz, 4H), 7.41 (m, 4H), 7.29 (m, 4H), 6.96 (d, J =1.3 Hz,, 2H), 6.89 (d, J =1.3 Hz, 2H), 3.72 (s, 4H), 2.57 (s, 4H), 2.28 (s, 6H), 2.24 (s, 6H). ^{13}C NMR (101 MHz, $CDCl_3$): δ 153.6, 132.4, 131.5, 130.3, 129.1, 128.5, 128.1, 126.7, 126.4, 125.4, 125.2, 124.9, 121.5, 62.0, 54.3, 42.0, 20.5. HRMS (APCI) m/z calcd. for $C_{48}H_{45}{}^{14}N_2O_2^+$ $[M+H]^+$ and for $C_{48}H_{45}{}^{15}N_2O_2^+$ $[M+H]^+$: 681.3476 and 683.3416; found: 681.3469 and 683.3419 (relative intensity ≈ 2.7/1.0).

*Synthesis of ***3***

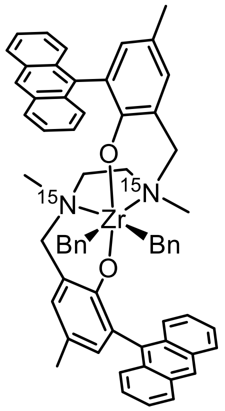

In a glovebox with argon atmosphere, ligand ***L3** (560 mg, 822 µmol) was dissolved in warm toluene (50 mL), and a solution of $ZrBn_4$ (375 mg, 822 µmol) in toluene (5 mL) was added. The mixture was stirred at r.t. overnight. Then, the solution was concentrated in vacuum, and the formed suspension was filtered through a glass frit. The precipitate was washed with 2 mL of toluene and 5 mL of pentane to give the product as a yellowish solid (538 mg, 69% yield, approx. 23% ^{15}N). ^1H NMR (400 MHz, CD_2Cl_2): δ 8.49 (s, 2H), 8.07 (dd, J_1 = 8.4 Hz, J_2 = 5.7 Hz, 4H), 7.67 (dd, J_1 = 14.5 Hz, J_2 = 8.5 Hz, 4H), 7.41–7.54 (m, 6H), 7.27 (m, 2H), 7.05 (d, J = 1.8 Hz, 2H), 6.81 (d, J = 1.8 Hz, 2H), 6.41 (m, 2H), 6.22 (t, J =7.6 Hz, 4H), 5.17 (d, J = 7.2 Hz, 4H), 3.37 (d, J = 13.8 Hz, 2H), 3.11 (m, 2H), 2.67 (d, J = 13.9 Hz, 2H), 2.30 (s, 6H), 1.77 (m, 2H), 1.76 (s, 6H), 0.41 (d, J = 8.5 Hz, 2H), -0.51 (d, J = 8.4 Hz, 2H). ^{13}C NMR (101 MHz, CD_2Cl_2): δ 156.4, 144.6, 135.6, 132.6, 132.01, 131.97, 131.2, 130.51, 130.50, 128.9, 128.8,

128.6, 128.3, 127.99, 127.95, 127.4, 126.8, 126.4, 125.8, 125.6, 125.4, 125.1, 124.7, 121.3, 63.3, 58.4, 45.7, 20.7.

Synthesis of 6

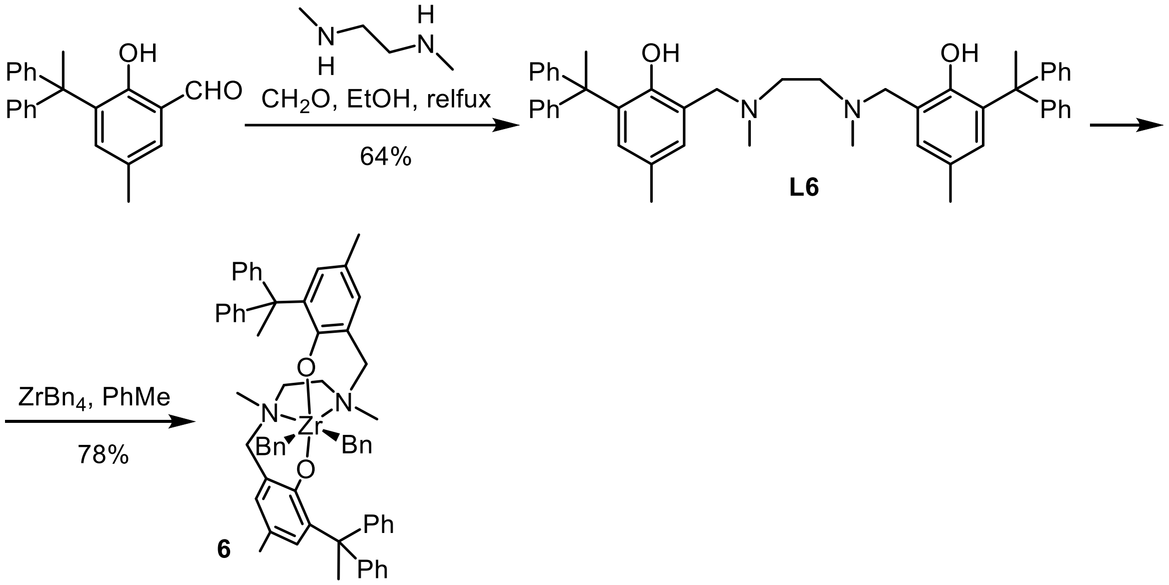

Synthesis of ***L6***

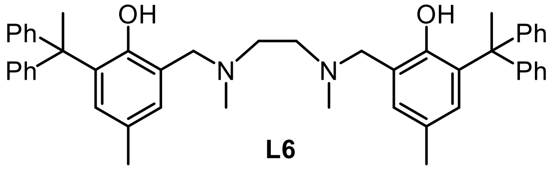

2-(2,2-Diphenylethyl)-4-methylphenol (5.9 g, 20.5 mmol) was dissolved in ethanol (250 mL), paraform (3.01 g, 103 mmol) and *N,N'*-dimethylethylenediamine (DMEDA, 1.1 mL, 10.3 mmol) were added, and the reaction mixture was refluxed for 17 h. After cooling to r.t., the mixture was concentrated *in vacuo*, the precipitate was filtered off, washed with methanol (50 mL) and dried *in vacuo*. The product was obtained as colorless crystals (4.52 g, 64 %). ^{1}H NMR (400 MHz, $CDCl_3$): δ 10.48 (br.s, 2H), 7.23-7.28 (m, 8H), 7.18-7.21 (m, 4H), 7.13-7.15 (m, 8H), 6.68 (d, J = 1.7 Hz, 2H), 6.63 (d, J = 1.7 Hz, 2H), 3.58 (s, 4H), 2.42 (s, 4H), 2.28 (s, 6H), 2.15 (s, 6H), 2.11 (s, 6H). ^{13}C NMR (101 MHz, C_6D_6): δ 153.7, 148.8, 135.2, 130.3, 128.4, 128.0, 127.6, 126.8, 125.5, 121.8, 61.9, 53.4, 51.6, 41.8, 27.7, 20.8. HRMS (APCI) m/z calcd. for $C_{48}H_{53}N_2O_2^+$ $[M+H]^+$: 689.4102 found: 689.4098.

Synthesis of ***6***

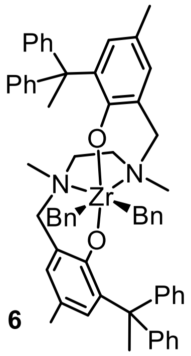

6

In a glovebox with argon atmosphere, ligand **L6** (1.0 g, 1.45 mmol) was dissolved in warm toluene (150 mL) and a solution of $ZrBn_4$ (661 mg, 1.45 mmol) in toluene (15 mL) was added. The mixture was stirred at r.t. overnight. Then, the solution was concentrated in vacuum and the suspension formed was filtered through a glass frit to give the product as a yellow solid (1.09 g, 78 %). ^{1}H NMR (400 MHz, CD_2Cl_2): *δ* 7.20-7.32 (m, 16H), 6.99 (d, *J* = 7.5 Hz, 4H), 6.94 (t, *J* = 7.5 Hz, 4H), 6.71 (m, 2H), 6.94 (d, *J* = 7.5 Hz, 4H), 6.57 (s, 2H), 6.39 (s, 2H), 3.65 (d, *J* = 13.8 Hz, 2H), 2.80 (m, 2H), 2.66 (d, *J* = 13.8 Hz, 2H), 2.54 (s, 6H), 2.12 (m, 2H), 2.04 (s, 6H), 1.55-1.61 (m 4H), 1.51 (s, 6H). ^{13}C NMR (101 MHz, CD_2Cl_2): *δ* 157.0, 151.3, 148.8, 148.4, 136.6, 133.7, 129.9, 129.4, 129.0, 128.4, 128.2, 128.1, 127.2, 126.7, 126.3, 126.1, 121.0, 67.7, 63.7, 53.0, 52.7, 45.1, 28.6, 20.8.

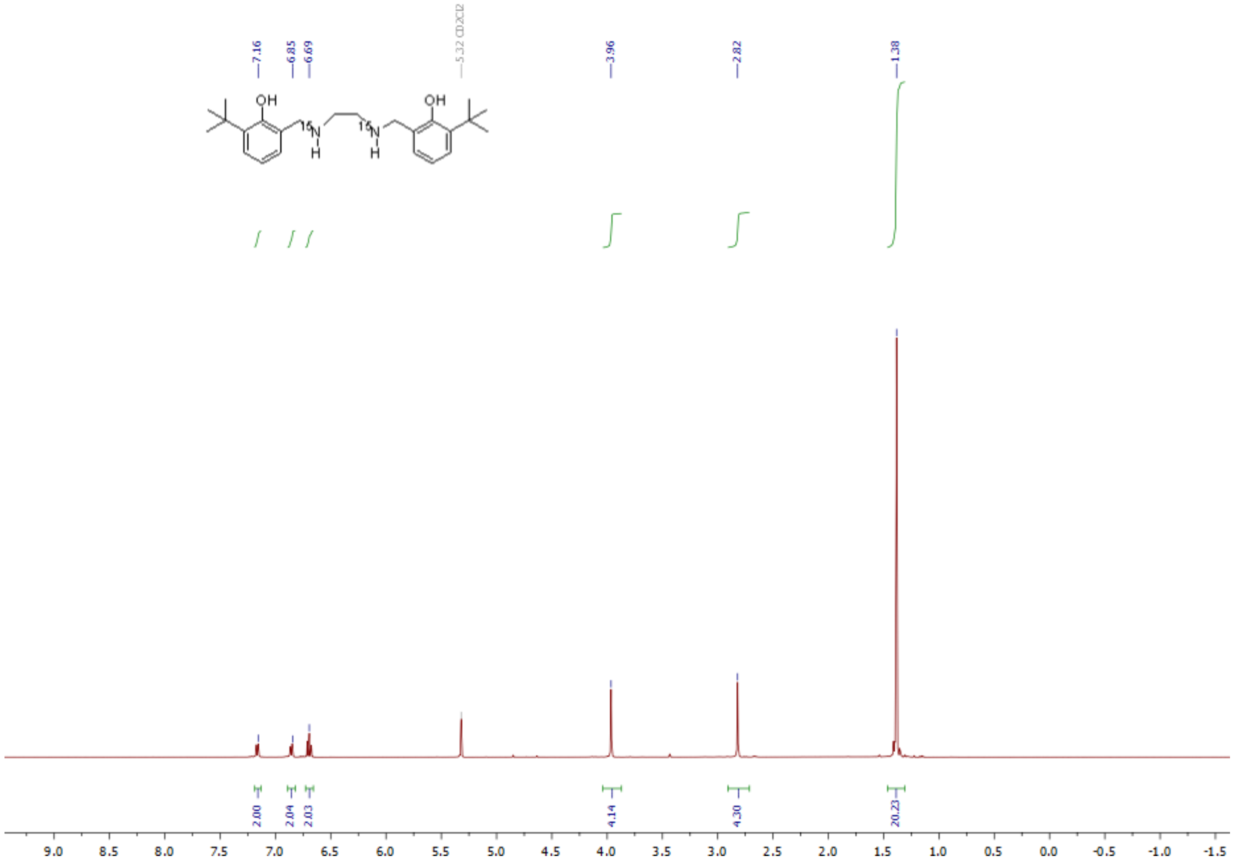

Figure S1. ^{1}H NMR spectrum of ligand ***L4H-H** in CD_2Cl_2 at room temperature.

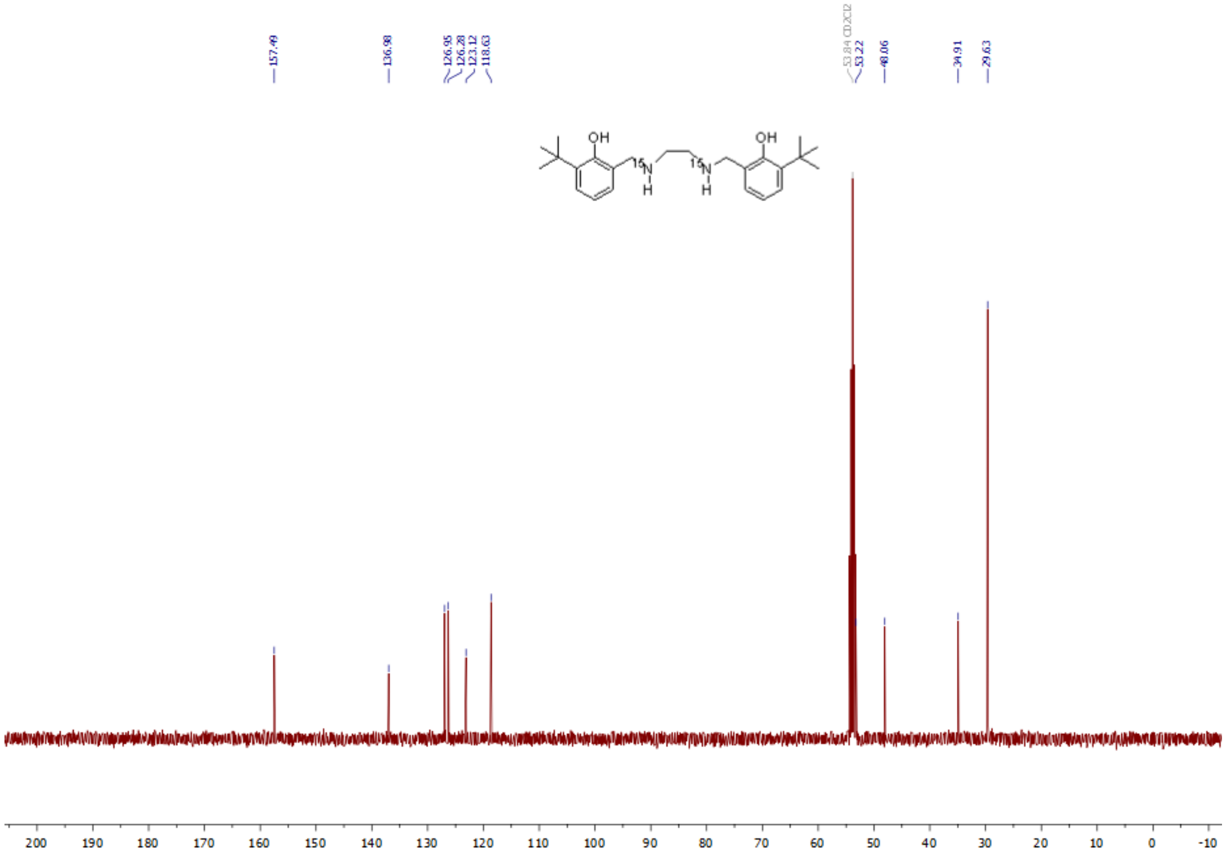

Figure S2. ^{13}C NMR spectrum of ligand ***L4H-H** in CD_2Cl_2 at room temperature.

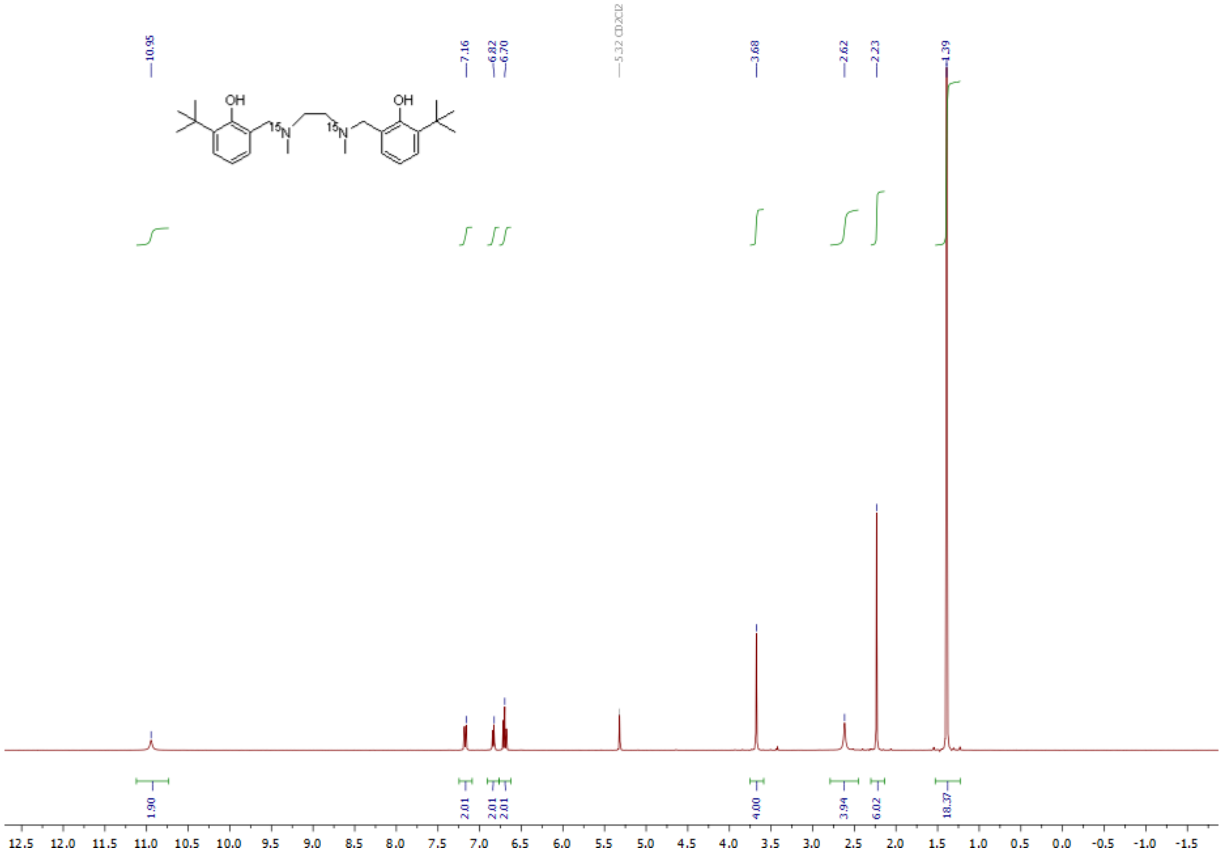

Figure S3. ^{1}H NMR spectrum of ligand ***L4H** in CD_2Cl_2 at room temperature

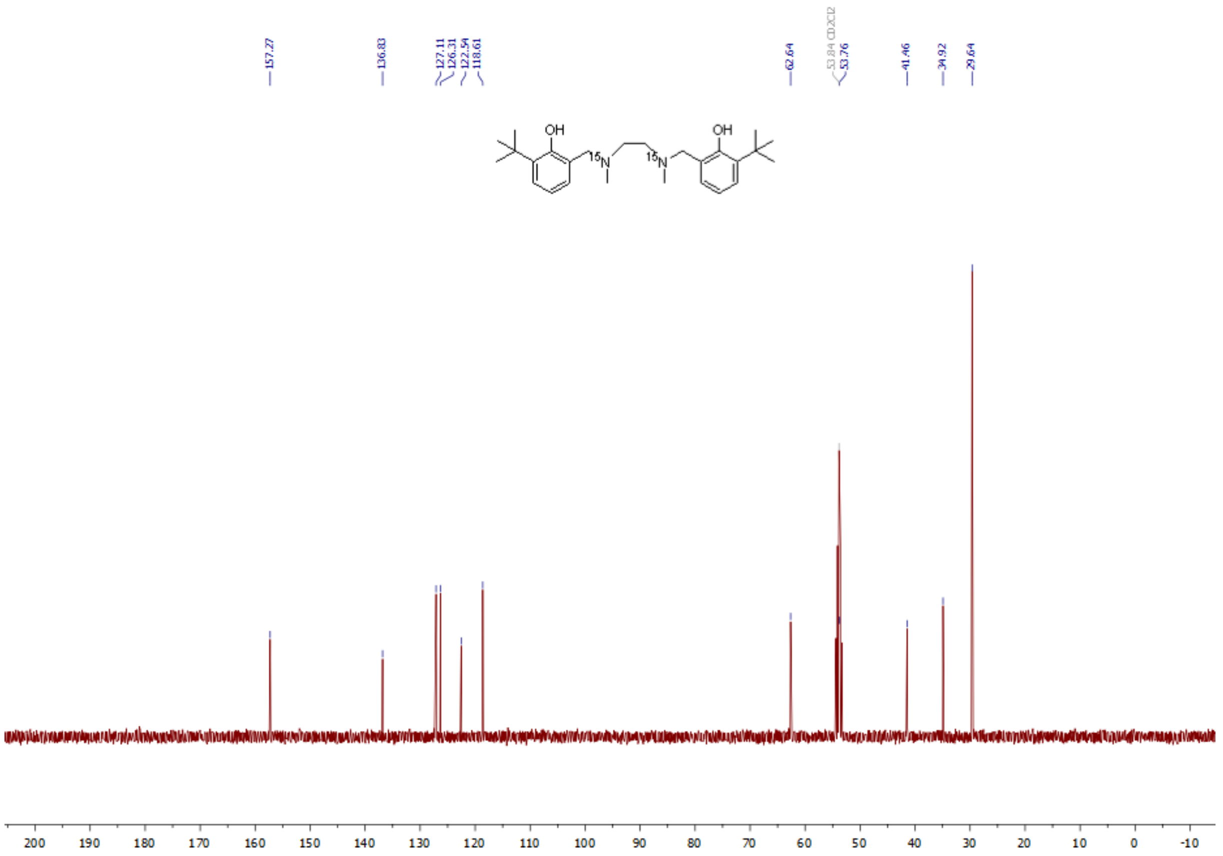

Figure S4. ^{13}C NMR spectrum of ligand ***L4H** in CD_2Cl_2 at room temperature

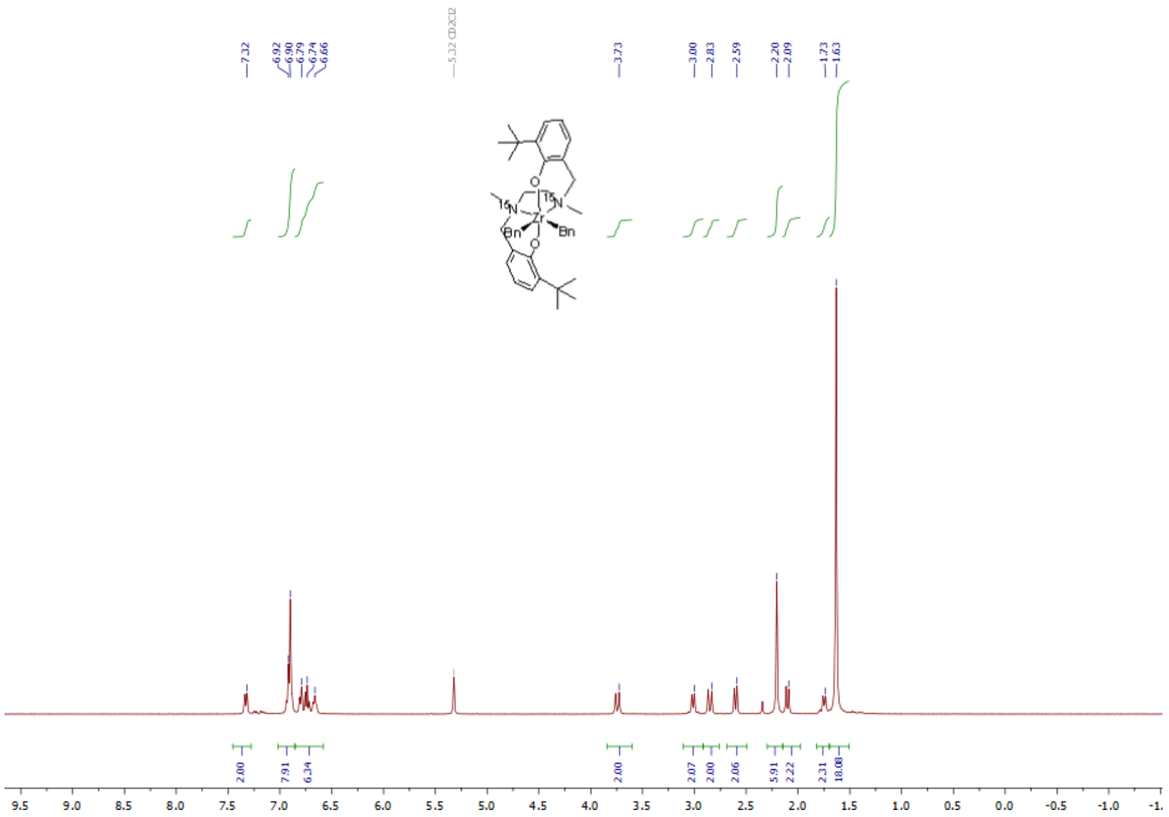

Figure S5. ^{1}H NMR spectrum of complex ***4H** in CD_2Cl_2 at room temperature

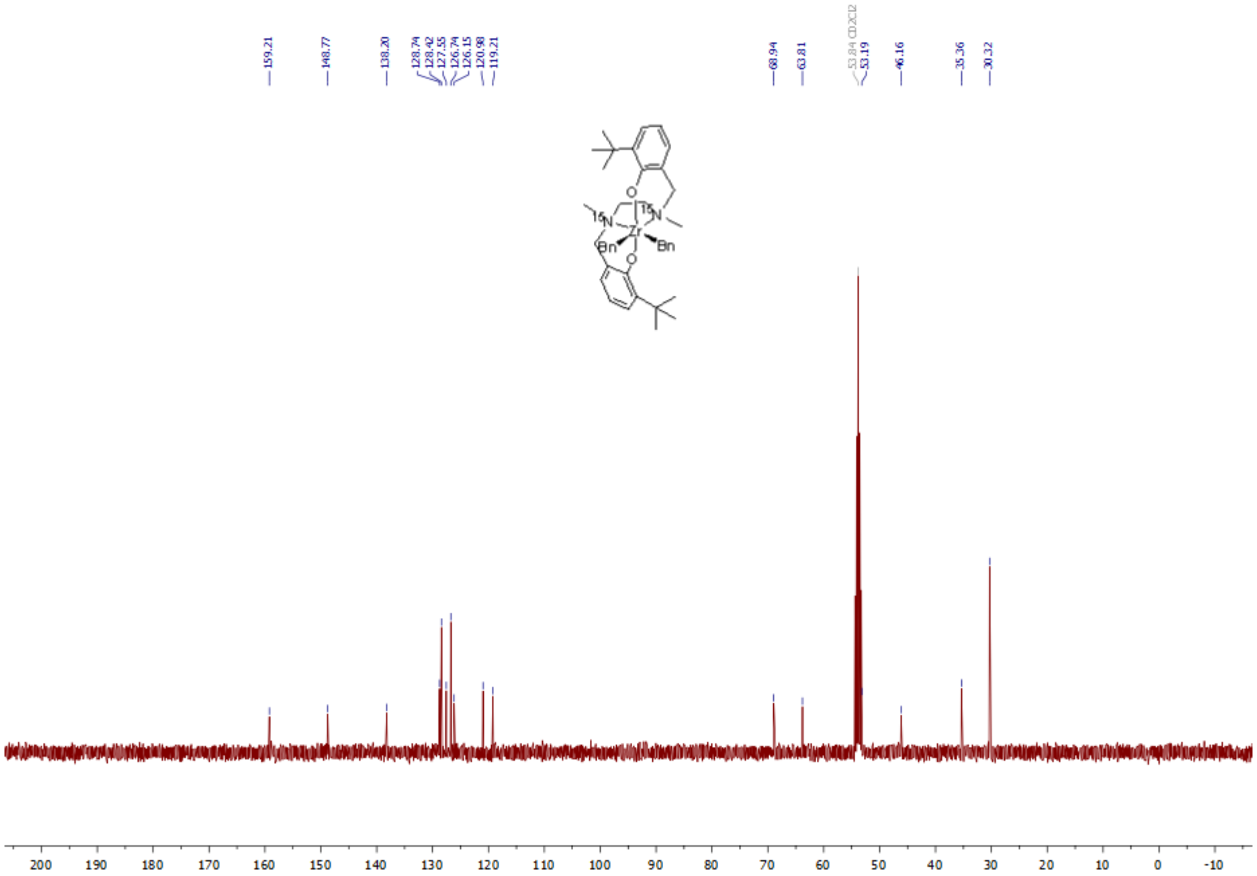

Figure S6. ^{13}C NMR spectrum of complex ***4H** in CD_2Cl_2 at room temperature

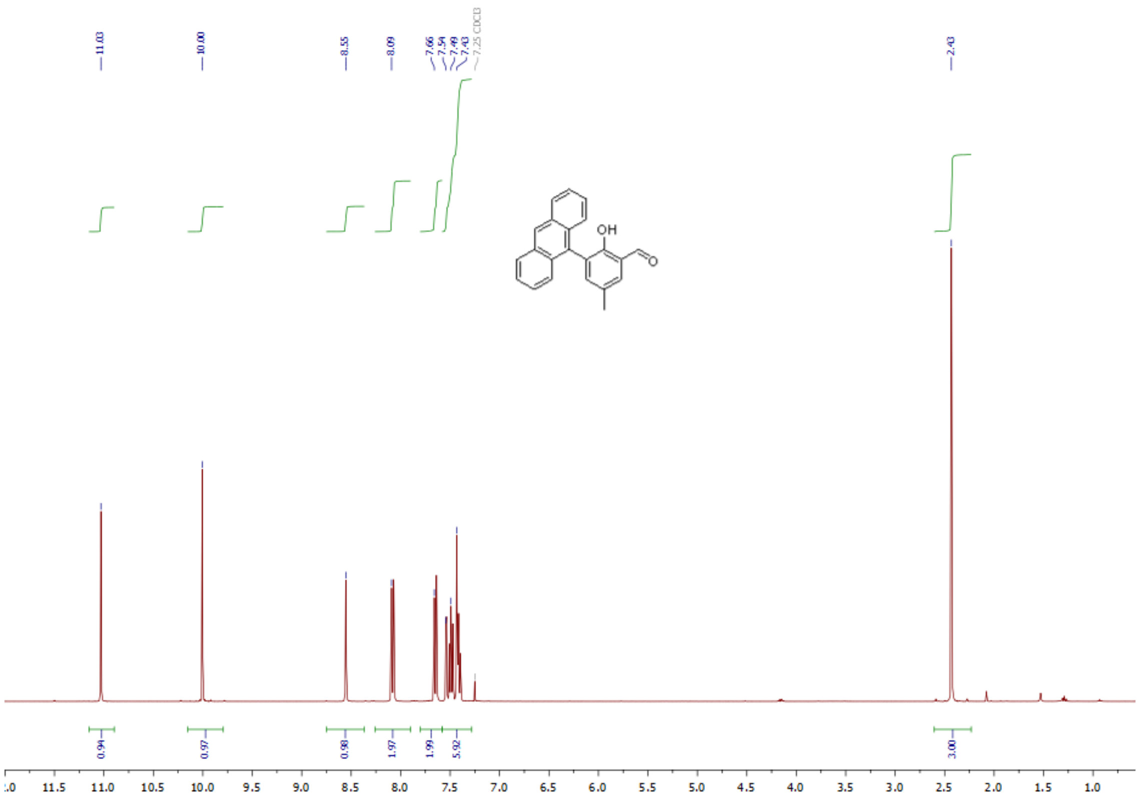

Figure S7. ^{1}H NMR spectrum of 3-(anthracen-9-yl)-2-hydroxy-5-methylbenzaldehyde in $CDCl_3$ at room temperature

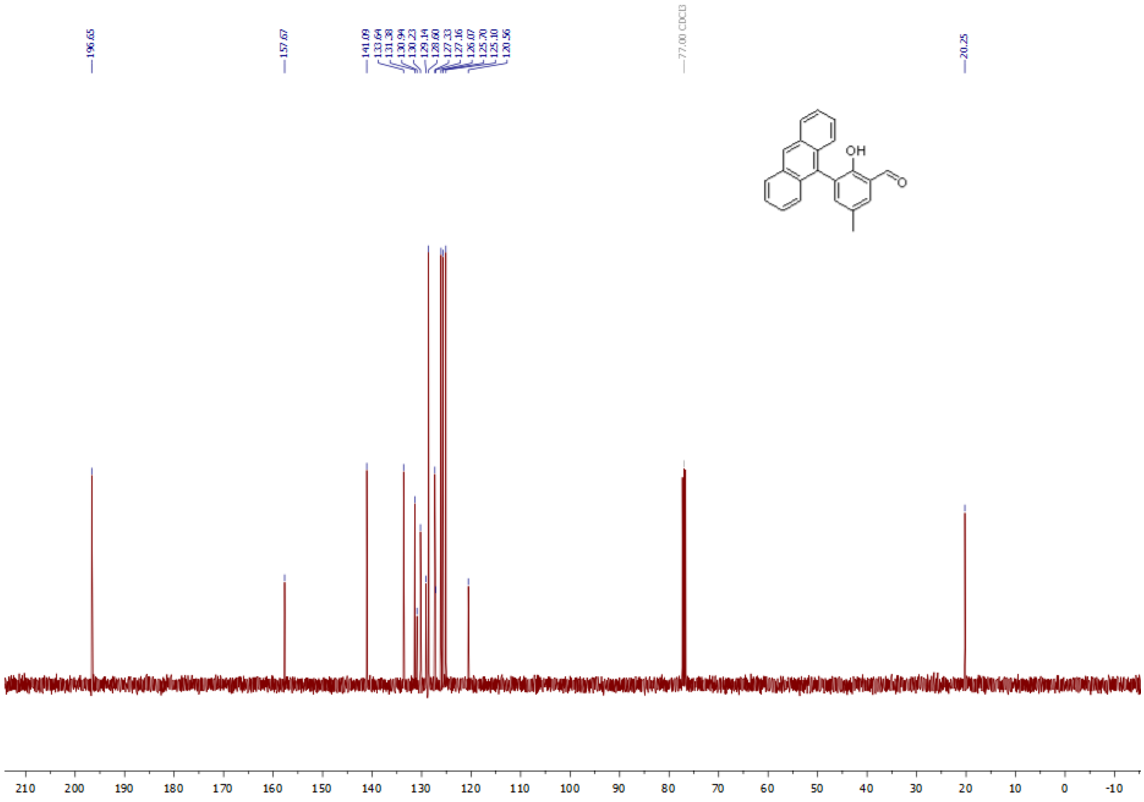

Figure S8. ^{13}C NMR spectrum of 3-(anthracen-9-yl)-2-hydroxy-5-methylbenzaldehyde in $CDCl_3$ at room temperature

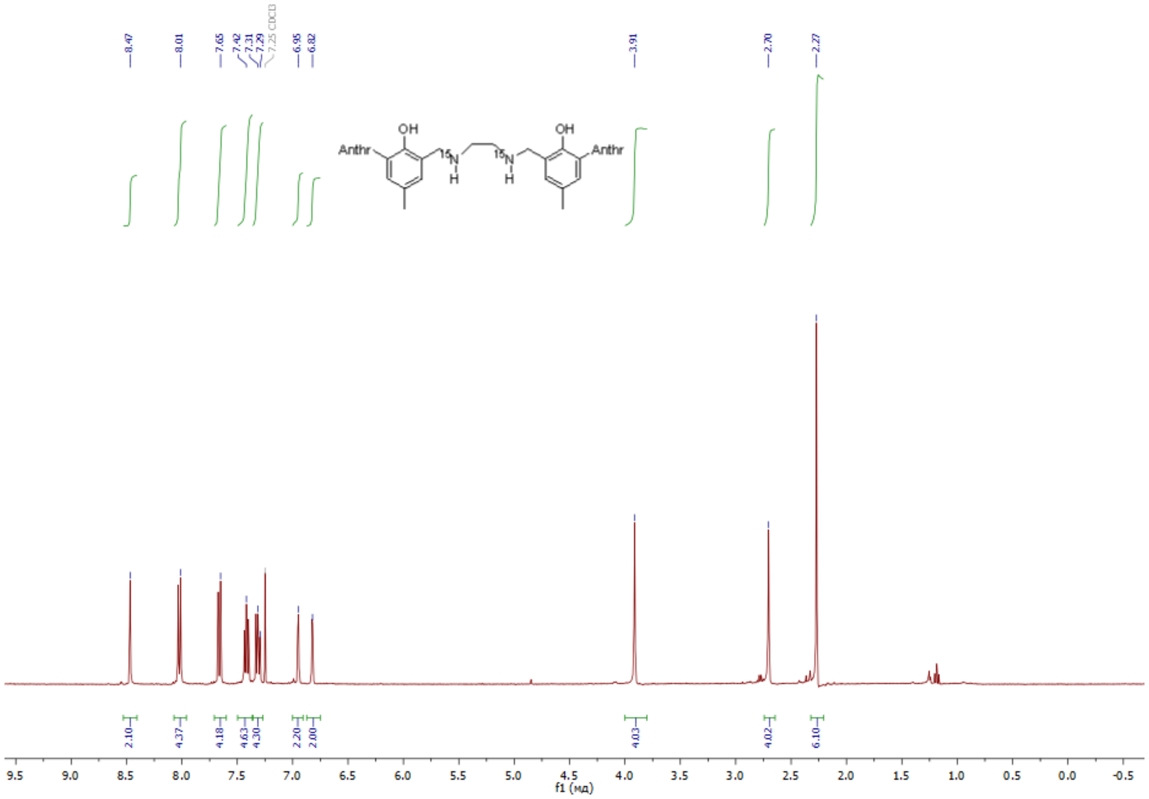

Figure S9. ^{1}H NMR spectrum of ligand ***L3-H** in $CDCl_3$ at room temperature

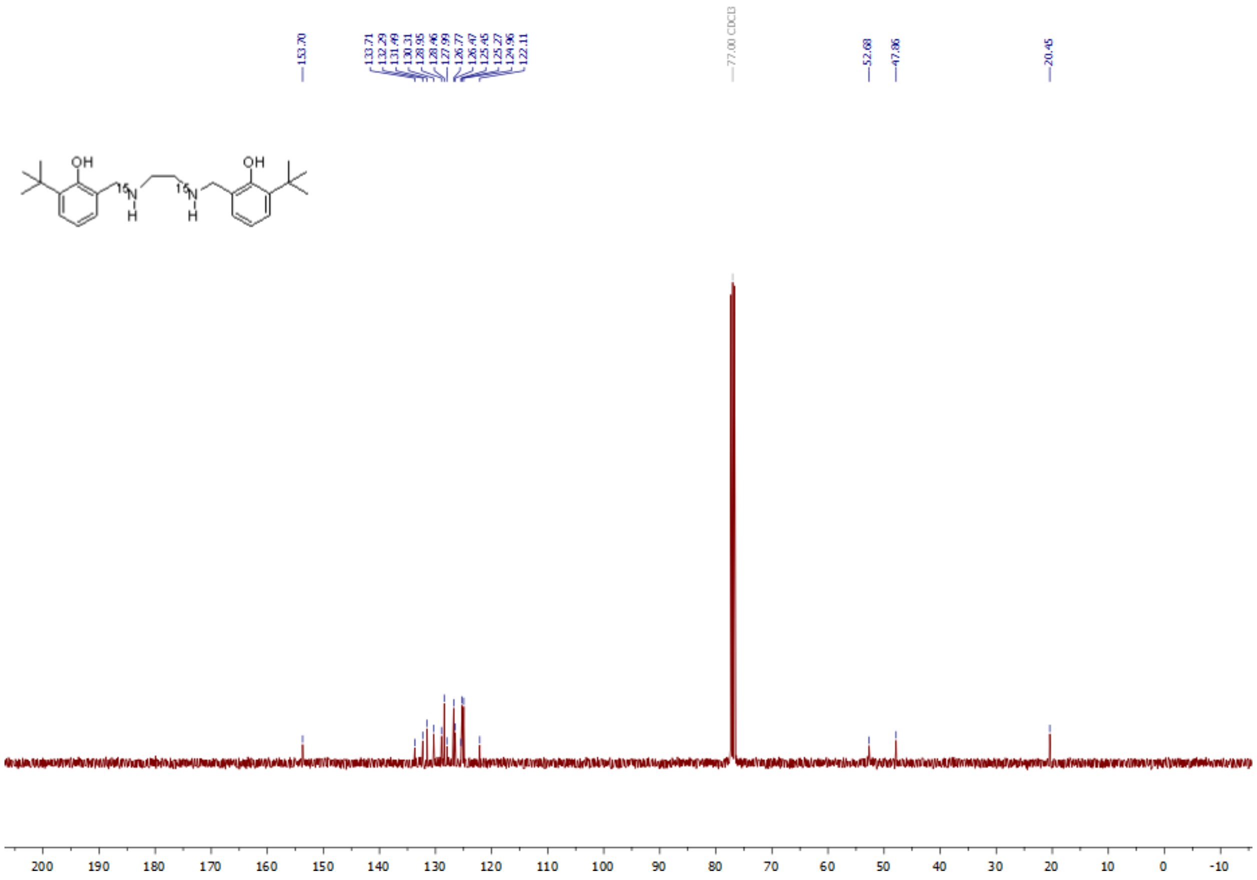

Figure S10. ^{13}C NMR spectrum of ligand ***L3-H** in $CDCl_3$ at room temperature

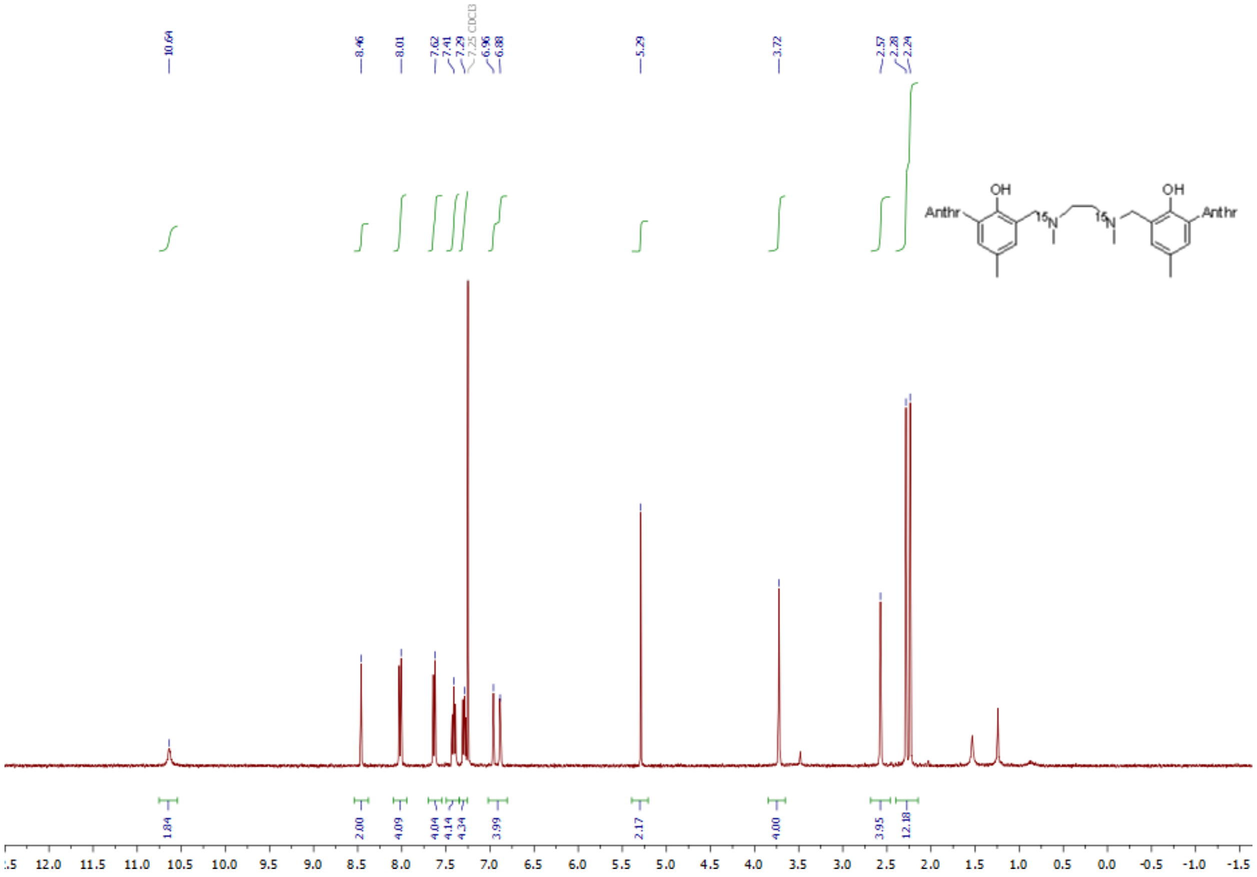

Figure S11. ^{1}H NMR spectrum of ligand ***L3** in $CDCl_3$ at room temperature

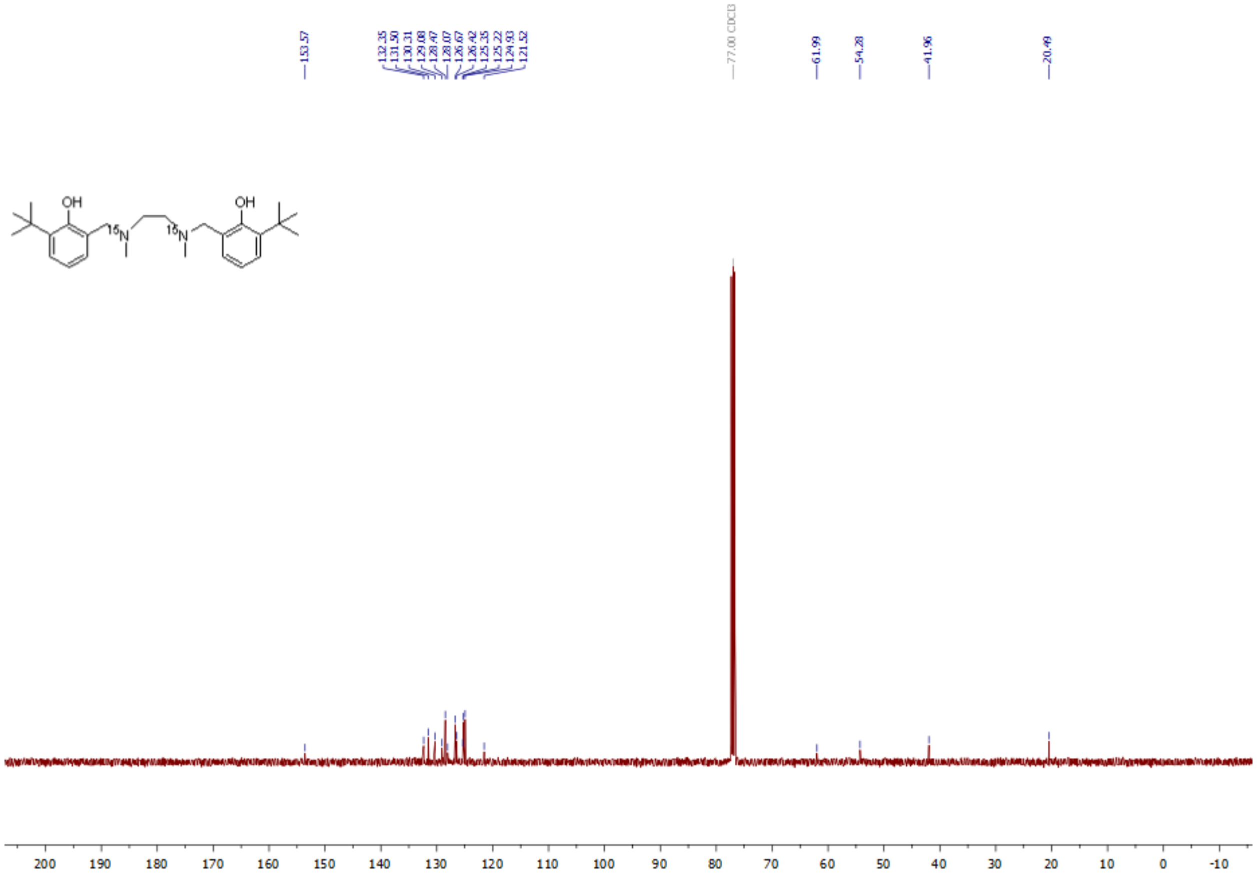

Figure S12. ^{13}C NMR spectrum of ligand ***L3** in $CDCl_3$ at room temperature

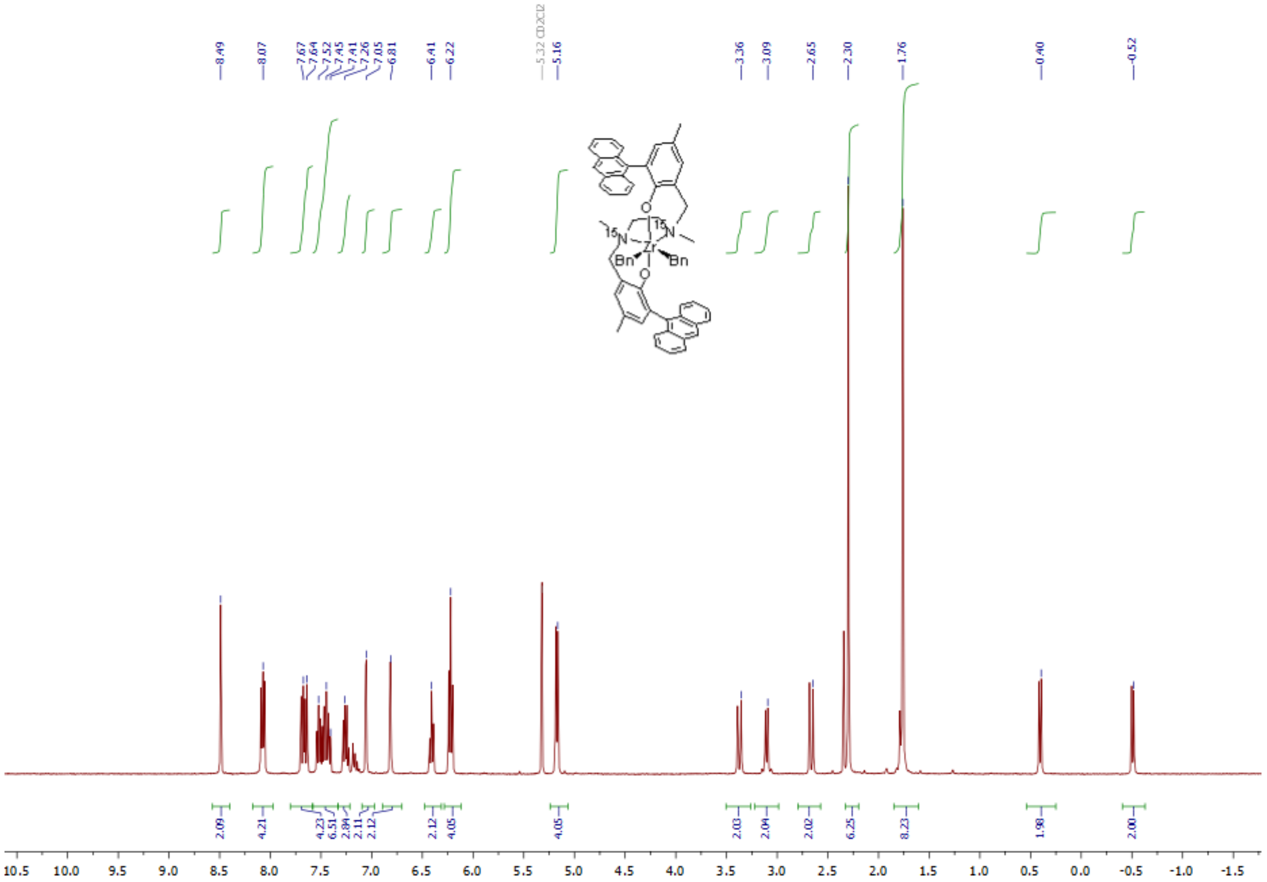

Figure S13. ^{1}H NMR spectrum of complex ***3** in CD_2Cl_2 at room temperature

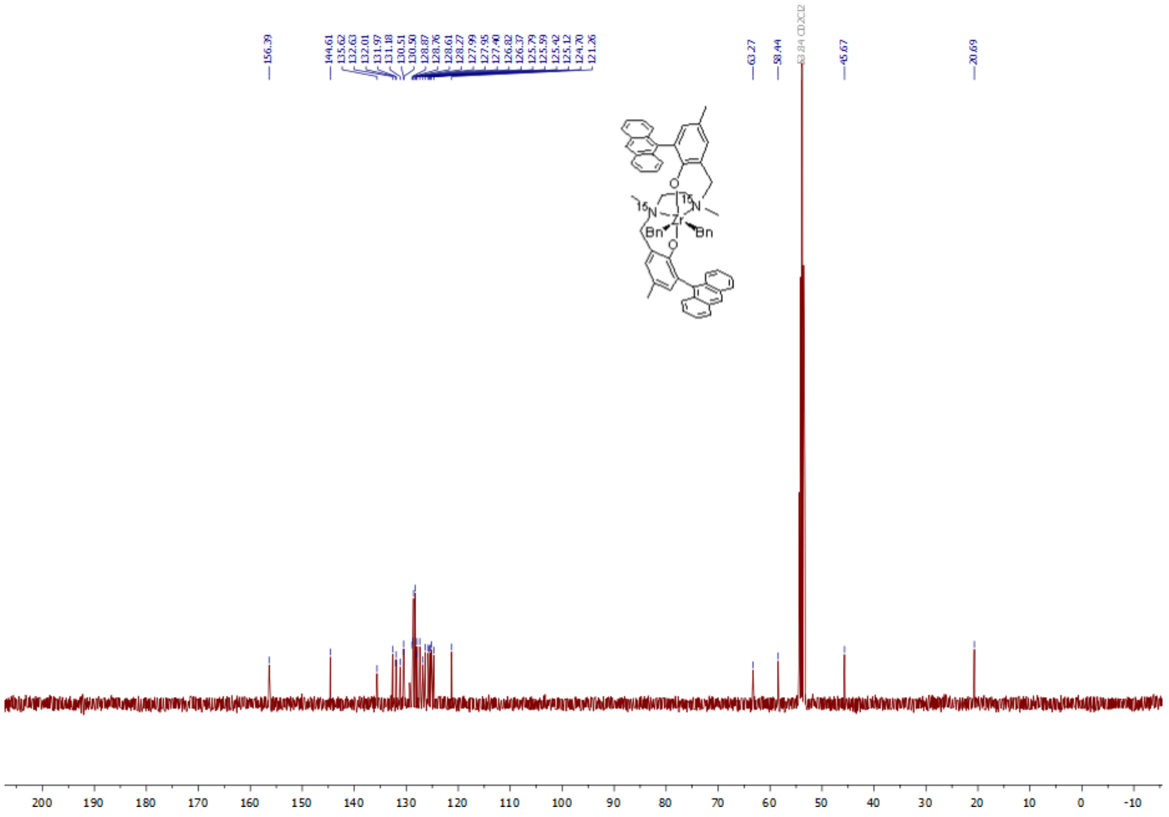

Figure S14. ^{13}C NMR spectrum of complex ***3** in CD_2Cl_2 at room temperature.

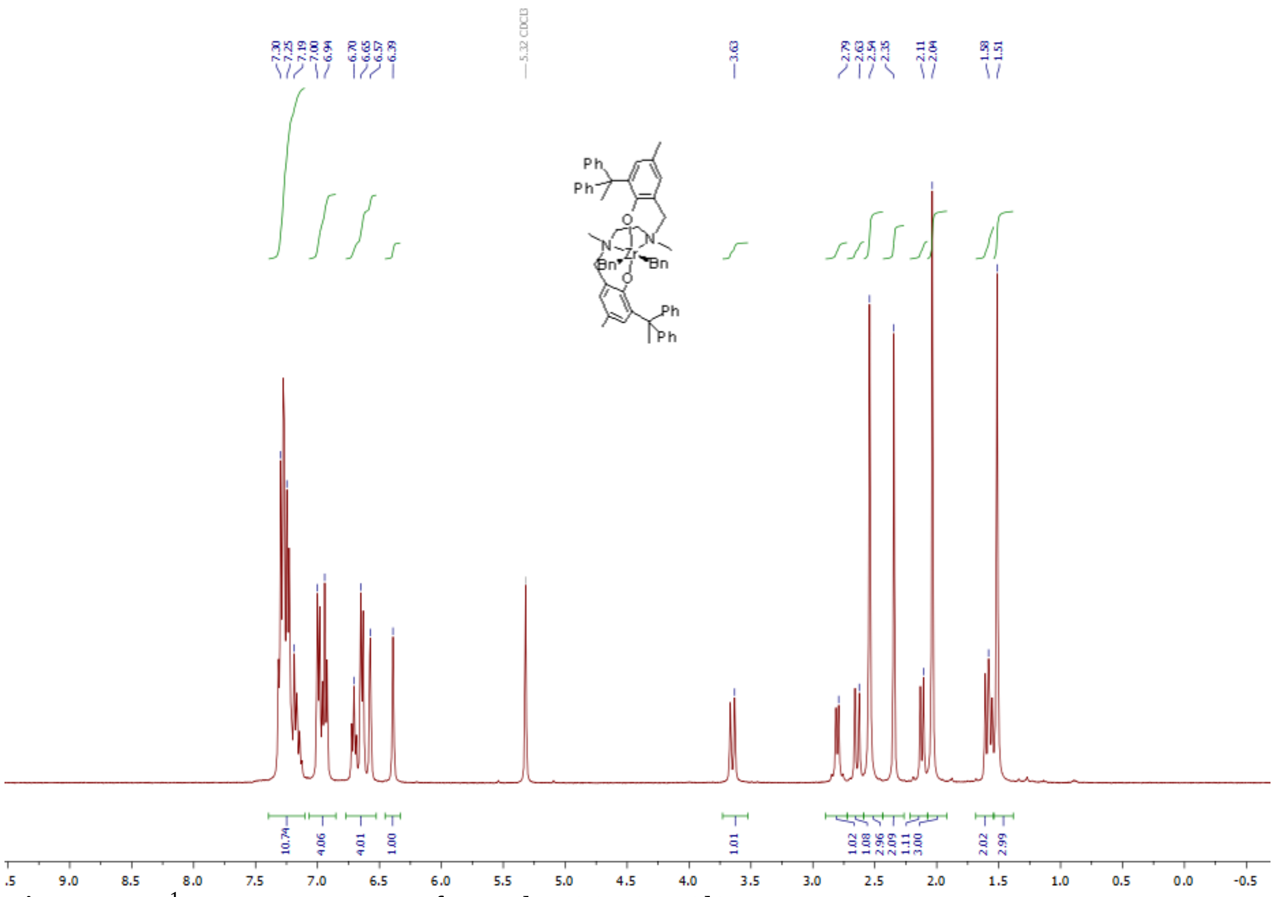

Figure S15. ^{1}H NMR spectrum of complex **6** in CD_2Cl2 at room temperature

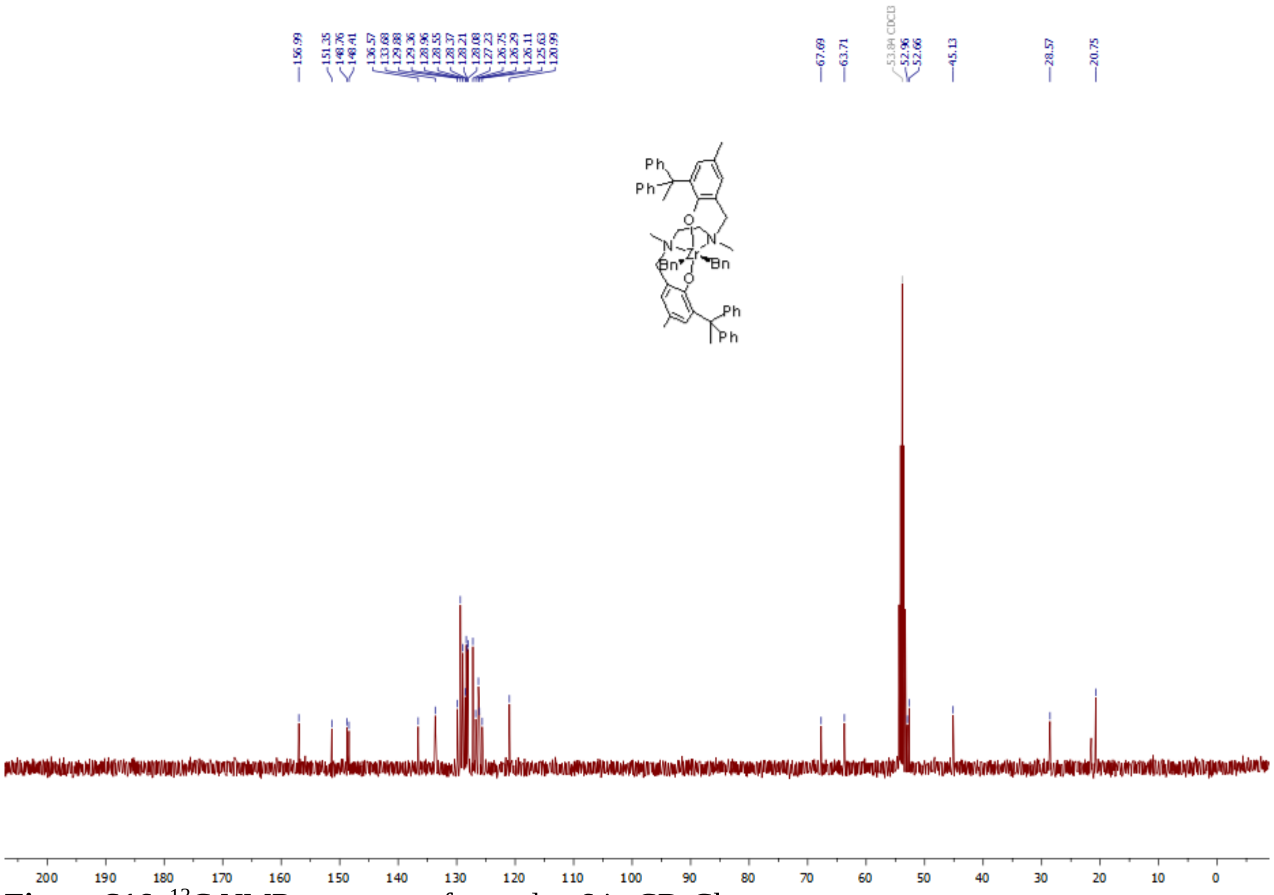

Figure S16. ^{13}C NMR spectrum of complex **6** in CD_2Cl_2 at room temperature

Activation of Salan complexes

<u>*In situ* synthetic procedure</u>: In the glovebox, 1eq of the neutral complex of choice and 0.95 eq of TTB were loaded into a J. Young NMR tube and dissolved in 0.6 mL of the deuterated solvent of choice (toluene-d_8 or chlorobenzene-d_5). The colour of the resulting solutions was orange or yellow, and in some cases the precipitation of a red oil was observed. The solutions were left to decant for approximately 30 min before NMR characterization and experiments.

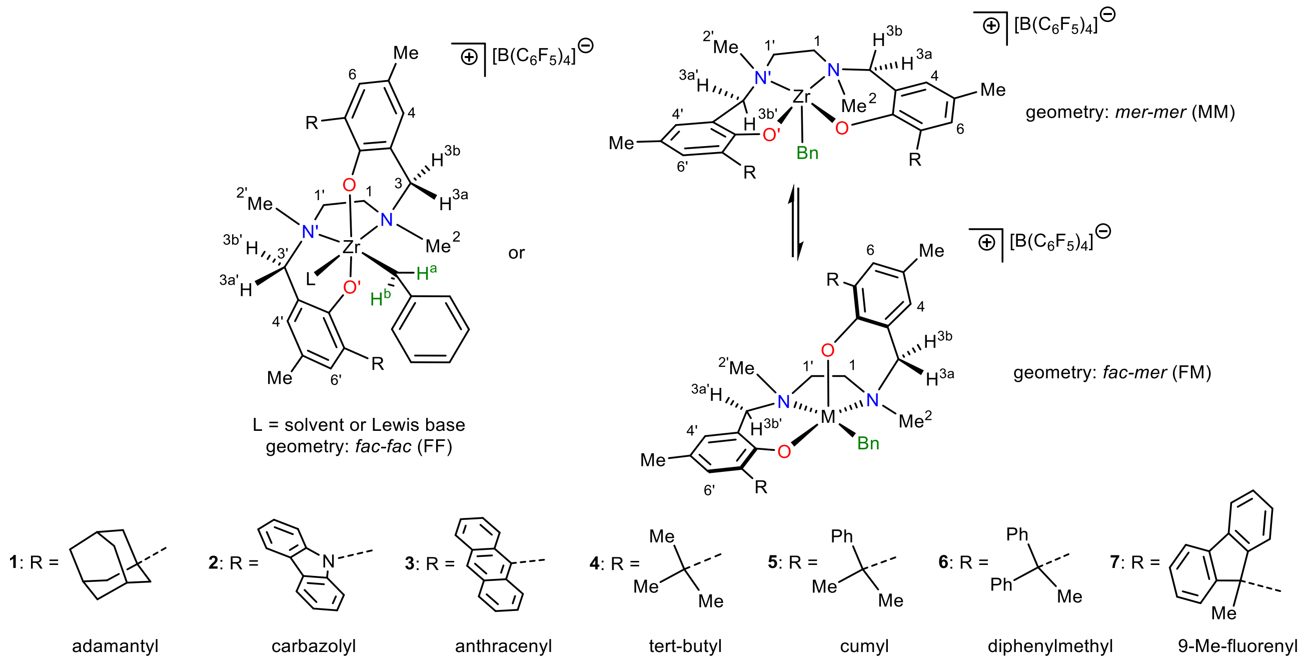

1⁺. ^{1}H NMR (400 MHz, C_7D_8, 233K) δ 6.59 (s, 3H, H4 or H4'), 6.51 (s, 3H, H4 or H4'), 2.20 (s, 3H, H5 or H5'), 2.03 (m, 6H, H5 or H5'+ H2 or H2'), 1.64 (s, 3H, H2 or H2').

^{19}F NMR (400 MHz, C_7D_8): δ -131.7 (br, 8F, o-F), -161.5 (br, 4F, p-F), -165.6 (br, 8F, m-F). The concentration in toluene was too low to characterize the compound completely.

^{1}H NMR (400 MHz, C_6D_5Cl, 233K) δ 7.49 (d, J = 7.3 Hz, 1H, Ho), 7.19 (s buried, H6'), 7.18 (d buried, Ho '), 7.15 (s buried, H6), 6.92 (t buried, Hp), 6.75 (m, 1H, Hm'), 6.60 (s, 1H, H4), 6.44 (t, J = 7.7 Hz, 1H, Hm), 6.39 (s, 1H, H4'), 4.51 (d, J = 13.0 Hz, 1H, H3), 3.60 (d, J = 14.3 Hz, 1H, H3'), 3.12 (d, J = 8.4 Hz, 1H, Ha), 3.07-2.98 (m, 2H, Hb + H3), 2.94 (t, 1H, H1), 2.70 (d, J = 14.3 Hz, 1H, H3'), 2.59 (t, 1H, H1'), 2.32 (s, 3H, H5), 2.24 (s, 3H, H2), 2.22 (s, 3H, H5'), 1.80 (s, 3H, H2'), 1.62 (d, J = 12.6 Hz, 1H, H1), 1.31 (d, J = 12.2 Hz, 1H, H1').

^{13}C NMR (400 MHz, C_6D_5Cl): δ 156.0 (C-OPh), 154.1 (C-OPh'), 137.7 (Cm'), 132.0 (C6'), 131.9 (Co'), 131.7 (Cm), 131.3 (Cipso), 130.8 (Co), 130.3 (Cp), 130.0 (C6), 129.7 (C4), 129.4 (C4'), 72.6 (Cab), 66.0 (C3), 63.6 (C3'), 55.2 (C1), 51.6 (C1'), 48.0 (C2), 46.6 (C2'), 20.8 (C5), 20.7 (C5').

^{19}F NMR (400 MHz, C_6D_5Cl): δ -131.6 (d, JoF-mF = 10.5, 8F, o-F), -162.0 (t, JpF-mF = 20.8 Hz, 4F, pF), -165.9 (m, 8F, mF).

2^+. ^{1}H NMR (400 MHz, C_7D_8, 233K) δ 8.19 – 7.77 (m, 4H, H carbazolyl), 6.74 (s, 1H, H6'), 6.50 (s, 1H, H6), 6.44 (s broad, H4'), 6.23 (s broad, H4), 3.87 (broad, H3), 2.83 (broad, H1' + H3), 2.36 (broad, H1), 2.22 (broad, H3'), 2.10 (m, H5 + H5' + toluene), 1.88 (H2), 1.75 (broad, H1'), 1.36 (broad, H1), 1.09 (H2'), 0.93 (m, H3' + pentane).

^{13}C NMR (400 MHz, C_7D_8) δ 65.0 (C3), 60.3 (C3'), 54.8 (C1'), 52.1 (C1), 48.7 (C2), 44.1 (C2'), 19.9 (C5'), 20.2 (C5).

^{19}F NMR (400 MHz, C_7D_8): δ -131.6 (s br, 8F, o-F), -161.6 (t br, 4F, pF), -165.5 (m br, 8F, mF). The concentration in toluene was too low to characterize the compound completely.

^{1}H NMR (400 MHz, C_6D_5Cl, 233K) δ 6.48 (t, 1H, Hp), 6.00 (t, 1H, Hm), 5.89 (d, 1H, Ho'), 5.83 (t, 1H, Hm'), 4.65 (d, 1H, Ho), 4.03 (d, J = 13.6 Hz, 1H, H3), 3.03 (d, J = 13.6 Hz, 1H, H3), 2.92 (t, J = 12.1 Hz, 1H, H1'), 2.54 (t broad, 1H, H1), 2.36 (d, J = 15.6 Hz, 1H, H3'), 2.28 (s, 3H, H5'), 2.24 (d, J = 7.5 Hz, 1H, Hb), 2.18 (d buried, 1H, H3'), 2.16 (s, 3H, H5), 1.98 (s, 3H, H2), 1.83 (d, J = 12.1 Hz, 1H, H1'), 1.50 (s, 3H, H2' + H1) 1.31 (d, J = 7.5 Hz, 1H, Ha).

^{13}C NMR (400 MHz, C_6D_5Cl) δ 65.0 (C3), 61.8 (C3'), 54.8 (C1'), 51.6 (C1), 47.7 (C2), 45.9 (C2').

^{19}F NMR (400 MHz, C_6D_5Cl): δ -131.7 (br, JoF-mF = 13.2, 8F, o-F), -161.5 (t, JpF-mF = 21.3 Hz, 4F, pF), -165.4 (m, 8F, mF).

3^+. ^{1}H NMR (400 MHz, C_7D_8, 233K) δ 6.76 (m, H4'), 6.56 (m, H4), 3.87 (broad, H3), 2.98 (broad, H1'), 2.84 (broad, H3), 2.49 (broad, H1), 2.23 (broad, H3'), 2.22 (H5'), 2.15 (H5), 1.87 (H2), 1.75 (broad, H1'), 1.36 (broad, H1), 1.16 (H2').

^{13}C NMR (400 MHz, C_7D_8) δ 65.0 (C3), 60.7 (C3'), 54.8 (C1'), 52.0 (C1), 48.5 (C2), 44.3 (C2').

^{19}F NMR (400 MHz, C_7D_8): δ -131.6 (br, 8F, o-F), -161.6 (br, 4F, p-F), -165.6 (br, 8F, m-F). The concentration in toluene was too low to characterize the compound completely.

^{1}H NMR (400 MHz, C_6D_5Cl, 233K) δ 6.89 (d, J = 12.2 Hz, 1H, H6'), 6.86 (d, J = 12.2 Hz, 1H, H6), 6.80 (d, J = 12.2 Hz, 1H, H4), 6.54 (d, J = 12.2 Hz, 1H, H4'), 6.39 (t, J = 7.6 Hz, 1H, Hp), 5.89 (d, J = 12.2 Hz, 1H, Ho'), 5.73 (t, J = 7.6 Hz, 1H, Hm), 5.61 (t, J = 7.6 Hz, 1H, Hm'), 4.01 (m, 2H, Ho + H3), 3.05 - 2.90 (m, H, H1 + H3), 2.56 (m, H1'), 2.32 (s, 3H, H5), 2.27 (d, J = 14.3 Hz, 1H, H3'), 2.19 (s, 3H, H5'), 2.16 (d buried, Ha), 2.02 (d, J = 14.3 Hz, 1H, H3'), 1.93 (s, 3H, H2), 1.79 (d, J = 11.6 Hz, 1H, H1), 1.44 (d, J = 13.1 Hz, 1H, H1'), 1.37 (s, 3H, H2'), 1.02 (d, J = 7.0 Hz, 1H, Hb).

^{13}C NMR (400 MHz, C_6D_5Cl) δ 136.6 (Co), 134.8 (Cm'), 133.3 (C6'), 132.9 (C6), 131.0 (C4), 130.9 (Co'), 130.7 (Cm), 130.4 (C4'), 130.0 (Cp), 128.8 (Cipso), 71.2 (Cab), 65.4 (C3), 61.9 (C3'), 54.5 (C1), 51.6 (C1'), 47.7 (C2), 45.6 (C2'), 20.7 (C5), 20.5 (C5').

^{19}F NMR (400 MHz, C_6D_5Cl): δ -131.7 (m, 8F, o-F), -161.4 (t, JpF-mF = 21.4 Hz, 4F, pF), -165.3 (m, 8F, mF).

$*3^+$-THF. ^{1}H NMR (400 MHz, C_6D_5Cl, 233K) δ 6.92 (m, H6'), 6.88 (m, H6), 6.83 (m H4'), 6.79 (“s”, 1H, H4), 6.62 (d, J = 7.5 Hz, 2H, Ho), 6.40 (t, J = 7.5 Hz, 1H, Hp), 5.96 (t, J = 7.56 Hz, 2H, Hm), 4.07 (d, J = 14.2 Hz, 1H, H3), 3.09 (m, H3'), 3.05 (m, H1), 2.93 (d, J = 14.2 Hz, 1H, H3), 2.83 (m, H1' + H3'), 2.81 (m, 2H, THF), 2.62 (m, 2H, THF), 2.26 (s, 3H, H5'), 2.23 (s, 3H, H5), 1.97 (d, J = 7.8 Hz, 1H, Hb), 1.89 (s, 3H, H2), 1.76 (d, J = 14.0 Hz, 1H, H1), 1.57 (d, J = 12.9 Hz, 1H, H1'), 1.45 (s, 3H, H2'), 0.97 (d, J = 7.8 Hz, 1H, Ha), 1.18 (m, 2H, THF), 0.68 (m, 2H, THF).
^{13}C NMR (400 MHz, C_6D_5Cl) δ 133.4 (C6'), 133.2 (C6), 131.1 (C4'), 131.0 (Co), 129.9 (Cm), 130.6 (C4), 126.7 (Cp), 132.2 (Cipso), 78.8 (THF), 69.1 (Cab), 65.2 (C3), 63.0 (C3'), 54.7 (C1), 51.8 (C1'), 46.9 (C2), 45.3 (C2'), 24.6 (THF), 20.2 (C5, C5').
^{19}F NMR (400 MHz, C_6D_5Cl): δ -131.5 (m, 8F, o-F), -161.2 (t, JpF-mF = 20.8 Hz, 4F, pF), -164.9 (m, 8F, mF).

$*3^+$-3,5-lutidine. ^{1}H NMR (400 MHz, C_6D_5Cl, 298K) δ 6.94 (s, 1H, H5'), 6.78 (m, H4 + Ho' lutidine), 6.71 (s, 1H, H4'), 6.54 (s, 1H, H5), 6.44 (s, 1H, Hp lutidine), 6.11 (d, J = 7.4 Hz, 1H, Ho'), 5.71 (t, J = 7.6 Hz, 1H, Hm), 5.58 (t, J = 7.6 Hz, 1H, Hp), 5.28 (t, J = 7.6 Hz, 1H, Hm'), 5.17 (s, 1H, Ho lutidine), 4.12 (m, 2H, H3 + Ho), 3.10 (t, J = 13.9 Hz, 1H, H1), 2.91 (m, 2H, H1' + H3), 2.66 (d, J = 14.6 Hz, 1H, H3'), 2.56 (d, J = 14.6 Hz, 1H, H3'), 2.23 (s, 3H, H5'), 2.20 (s, 3H, H5),2.05 (d, J = 6.5 Hz, 1H, Hb), 1.90 (s, 3H, H2), 1.69 (m, 4H, H1 + CH_3 lutidine), 1.60 (d, J = 12.6 Hz, 1H, H1'), 1.42 (s, 3H, H2'), 1.33 (s, 3H, CH_3 lutidine), 1.08 (d, J = 6.5 Hz, 1H, Ha).
^{13}C NMR (400 MHz, C_6D_5Cl, 298K) δ 146.5 (Co' lutidine), 144.9 (Co lutidine), 140.9 (Cp lutidine), 135.2 (Co), 133.8 (C6), 133.3 (C6'), 131.6 (Co'), 131.4 (C4), 131.1 (C4'), 131.0 (Cm), 129.9 (Cm'), 128.9 (Cp), 131.2 (Cipso), 66.9 (Cab), 65.2 (C3), 63.1 (C3'), 54.1 (C1), 52.0 (C1'), 47.1 (C2), 45.4 (C2'), 20.7 (C5'), 20.5 (C5), 18.0 and 17.5 (CH_3 lutidine).
^{19}F NMR (400 MHz, C_6D_5Cl): δ -131.5 (m, 8F, o-F), -161.3 (t, JpF-mF = 21.4 Hz, 4F, pF), -165.0 (m, 8F, mF).

4^+. ^{1}H NMR (400 MHz, C_7D_8, 233K) δ 7.19 (s buried, H6 or H6'), 6.46 (s, 1H, H4 or H4'), 3.00 (s broad, 2H, H3), 2.68 (d, J = 14.5 Hz, 1H, H3'), 2.57 (m, 2H, Ha + H1), 2.41 (t, J = 13.7 Hz, 1H, H1'), 2.26 (s, 3H, H5'), 2.22 (m, H1'), 2.17 (s, 4H, H5 + H3'), 2.01 (d, J = 13.0 Hz, H1), 1.98 (d, J = 11.4 Hz, Hb), 1.88 (s, 3H, H2), 1.53 (s, 9H, tBu'), 1.46 (s, 9H, tBu), 1.37 (s, 3H, H2').
^{13}C NMR (400 MHz, C_7D_8, 233K) δ 73.1 (Cab), 64.8 (C3), 61.5 (C3'), 56.9 (C1), 54.9 (C1'), 41.9 (C2), 37.2 (C2'), 29.7 (CtBu), 29.6 (CtBu'), 20.5 (C5), 20.4 (C5').
^{19}F NMR (400 MHz, C_7D_8): δ -131.7 (m, 8F, o-F), -161.7 (t, JpF-mF = 21.0 Hz, 4F, pF), -165.8 (m, 8F, mF).

^{1}H NMR (400 MHz, C_6D_5Cl, 263K, MM species) δ 6.67 (s buried, H4 or H4'), 6.52 (s, 1H, H4 or H4'), 3.41 (m, 2H, H3), 2.91 (d, J = 14.6 Hz, 1H, H3'), 2.80 – 2.50 (m, 4H, H3' + H1' + H1 + Ha/b), 2.39 – 2.08 (m, 11H, H5'+ H5 + H2 + H1' + H1 + Ha/b), 1.69 (s, 3H, H2'), 1.52 (s, 9H, tBu'), 1.41 (s, 9H, tBu).

^{13}C NMR (400 MHz, C_6D_5Cl) δ 73.0 (Cab), 65.1 (C3), 62.0 (C3'), 57.7 (C1), 54.8 (C1'), 42.9 (C2), 38.4 (C2'), 29.8 (CtBu), 29.7 (CtBu'), 20.7 (C5), 20.6 (C5').

^{1}H NMR (400 MHz, C_6D_5Cl, 233K, FF species) δ 6.67 (s buried, H4 or H4'), 6.52 (s, 1H, H4 or H4'), 3.34 (d broad, 2H, H3), 2.99 (d broad, 1H, Ha/b), 2.86 (m, 3H, H3' + Ha/b), 2.78 – 2.49 (m, 2H, H1' + H1), 2.26 (s, 3H, H5'), 2.18 (s, 3H, H5), 2.13 (buried, 1H, H1), 2.00 (s, 3H, H2), 1.82 (s, 3H, H2'), 1.54 (s, 9H, tBu'), 1.34 (s broad, 10H, tBu + H1').

^{13}C NMR (400 MHz, C_6D_5Cl) δ 73.0 (Cab), 65.1 (C3), 61.8 (C3'), 57.2 (C1), 51.4 (C1'), 47.3 (C2, C2'), 30.5 (CtBu), 29.8 (CtBu'), 20.8 (C5), 20.7 (C5').

^{19}F NMR (400 MHz, C_6D_5Cl): δ -131.6 (d br, JoF-mF = 11.6, 8F, o-F), -161.8 (t, JpF-mF = 20.6 Hz, 4F, pF), -165.7 (m, 8F, mF).

$*4H^{+}$. ^{1}H NMR (400 MHz, C_7D_8, 268K) δ 7.23 (m, 1H, H6), 7.19 (m, Hm), 7.18 (m, 1H, H6'), 6.98 (m, Ho'), 6.89 (m, Hm'), 6.83 (m, 2H, Hp + H4), 6.79 (m, 1H, H5'), 6.64 (m, Ho + H4'), 3.02 (d, J = 12.8 Hz, 1H, H3), 2.95 (d, J = 12.8 Hz, 1H, H3), 2.73 (d, J = 14.1 Hz, H3'), 2.62 – 2.50 (m, 2H, H1 + Hb), 2.39 (t, J = 14.6 Hz, 1H, H1'), 2.27 – 2.09 (m, H1' + H3'), 1.99 (d, J = 12.0 Hz, H1), 1.96 (d, J = 11.7 Hz, 1H, Ha), 1.87 (s, 3H, H2), 1.50 (s, 9H, tBu'), 1.40 (s, 9H, tBu), 1.35 (s, 3H, H2').

^{13}C NMR (400 MHz, C_7D_8, 268K) δ 136.3 (Cipso), 129.8 (C4), 129.2 (Co), 129.1 (C5), 128.9 (C4), 128.7 (Co'), 128.2 (Cm), 127.9 (Hp), 127.8 (C6), 127.3 (C6'), 127.1 (Cm'), 126.6 (C5'), 127.8 (C4'), 73.6 (Cab), 64.6 (C3), 61.2 (C3'), 56.7 (C1), 54.9 (C1'), 41.7 (C2), 37.7 (C2'), 29.5 (CtBu), 29.4 (CtBu').

^{1}H NMR (400 MHz, C_6D_5Cl, 243K) δ 7.36 (very broad, Hp), 7.31 (d, buried, H6), 7.26 (d, J = 8.2 Hz, 1H, H6'), 7.03 (m, buried, Hm + Hm'), 6.98 (buried, H4 + H4'), 6.88 (buried, H5 + H5'), 3.33 (d br, J = 13.8 Hz, 2H, H3), 2.99 (d, J = 8.9 Hz, 1H, Ha), 2.90 (d br, J = 14.6 Hz, 2H, H3'), 2.72 (t, buried, H1), 2.66 (d, buried, Hb), 2.56 (t br, J = 13.0 Hz, H1'), 2.27 (br, H1'), 2.11 (d, buried, H1), 2.00 (s, 1H, H2), 1.81 (s, 1H, H2'), 1.50 (s, 1H, tBu'), 1.39 (s, 1H, tBu).

^{13}C NMR (400 MHz, C_6D_5Cl) δ 131.4 (C4'/C4), 129.7 (Cm'/Cm), 129.5 (Co, Co'), 129.1 (C5'/C5), 129.0 (C6), 128.9 (C6'), 128.6 (Cp), 127.6 (C5'/C5), 127.0 (Cipso), 126.3 (C4'/C4), 126.1 (Cm'/Cm), 73.7 (Cab), 65.2 (C3), 61.8 (C3'), 57.4 (C1), 55.0 (C1'), 47.5 (C2), 47.4 (C2'), 30.0 (CtBu), 29.9 (CtBu').

^{19}F NMR (400 MHz, C_6D_5Cl): δ -131.6 (m, 8F, o-F), -161.2 (t, JpF-mF = 21.4 Hz, 4F, pF), -165.1 (m, 8F, mF).

***4H$^+$-THF.** ^1H NMR (400 MHz, C_6D_5Cl, 292K) δ 7.33 (m, 2H, H6 + H6'), 7.20 – 7.11 (m, Ho + Ho' + Hm + C_6D_5Cl), 7.07 (m, 1H, Hm'), 6.94 (m, 1H, Hp), 6.88 (m, 1H, H5), 6.82 (m, H5'), 6.80 (m, 1H, H4'), 6.64 (m, 1H, H4), 4.39 (d, J = 14.3 Hz, 1H, H3), 3.50 (m, THF), 3.42 (d, J = 14.3 Hz, 1H, H3'), 3.02 (d, J = 14.3 Hz, 1H, H3), 2.96 (d, J = 14.3 Hz, 1H, H3'), 2.92 (t, J = 13.7 Hz, 1H, H1), 2.91 (d, J = 9.9 Hz, 1H, Hb), 2.71 (t, 1H, H1'), 2.66 (d, J = 9.9 Hz, 1H, Ha), 2.06 (s, 3H, H2), 1.90 (s, 3H, H2'), 1.58 (d, J = 14.8 Hz, 1H, H1), 1.49 (s, 9H, tBu'), 1.44 (d, 1H, H1'), 1.40-1.32 (m, THF), 1.31 (s, 9H, tBu).
^{13}C NMR (400 MHz, C_6D_5Cl) δ 135.1 (Cipso), 133.3 (Co), 131.3 (C4), 130.4 (Cm), 129.6 (C5'), 129.3 (C4'), 128.9 (C6, C6'), 128.5 (Co'), 127.6 (Cm'), 127.3 (C5), 127.1 or 126.1 (Cp), 76.3 (THF), 71.4 (Cab), 66.5 (C3), 63.6 (C3'), 55.3 (C1'), 51.5 (C1), 47.2 (C2), 47.1 (C2'), 30.3 (CtBu), 30.1 (CtBu').

***4H$^+$-3,5-lutidine.** ^1H NMR (400 MHz, C_6D_5Cl, 298K) δ 7.97 (s, 1H, Hp lutidine), 7.51 (m, 3H, H6 + H6'+ Hm'), 7.09 - 7.01 (m, buried, H4 + H5 + Ho,o' lutidine), 6.94 (t, J = 8.0 Hz, 1H, H5'), 6.83 (m, H4' + Hm), 6.47 (t, 1H, J = 7.97 Hz, Ho), 4.67 (d, J = 14.6 Hz, 1H, H3), 3.34 (d, J = 13.9 Hz, 1H, H3'), 3.24 (d, J = 14.6 Hz, 1H, H3), 3.13 (t, J = 14.2 Hz, 1H, H1), 3.06 (d, J = 8.6 Hz, 1H, Ha), 2.94 (t, J = 14.2 Hz, 1H, H1'), 2.84 (d, J = 13.9 Hz, 1H, H3'), 2.81 (d, J = 8.6 Hz, 1H, Hb), 2.29 (s, 3H, H2), 2.07 (s, 6H, CH_3 lutidine), 1.94 (s, 3H, H2'), 1.74 (d, J = 14.2 Hz, 1H, H1), 1.69 (s, 9H, tBu'), 1.60 (d, 1H, H1'), 1.59 (s, 9H, tBu).
^{13}C NMR (400 MHz, C_6D_5Cl) δ 141.62, 136.7 (Co,o' lutidine), 129.9 (Co, C5'/Cm), 129.7 (C4/C4'/C5/Co'), 129.3 (C5'/Cm), 129.1 (C6'/C6), 128.8 (C6'/C6), 127.4 (C4/C4'/C5/Co'), 126.2 (C4/C4'/C5/Co'), 122.0 (Cp), 131.9 (Cipso), 121.7 (C4/C4'/C5/Co'), 70.0 (Cab), 66.8 (C3), 64.0 (C3'), 55.0 (C1), 52.2 (C1'), 47.4 (C2), 47.4 (C2'), 30.4 (CH_3 tBu), 30.1 (CH_3 tBu'), 17.4 (CH_3 lutidine).

5$^+$. ^1H NMR (400 MHz, C_7D_8, 268K) δ 7.26 (s br, 1H, H6 or H6'), 7.22 (s buried, H6 or H6'), 6.73 (t, Hm Ph cumyl), 6.63 (m, Hp Ph cumyl), 6.62 (s buried, H4 or H4'), 6.51 (s br, 1H, H4 or H4'), 6.01 (d, J = 7.5 Hz, Ho Ph cumyl), 3.13 (br, H3 or H3'), 2.88 (d, J = 12.7 Hz, H3 or H3'), 2.63 (d, J = 14.1 Hz, H3 or H3'), 2.45 (m, 2H, H3 or H3' + H1 or H1'), 2.24 (s, 3H, H5 or H5'), 2.21 (s, 3H, H5 or H5'), 1.93 (d buried, H1 or H1'), 1.89 (s br, 3H, H2 or H2'), 1.82 (s br, 3H, Me cumyl), 1.67

(s br, 3H, Me cumyl), 1.62 (s br, 3H, Me cumyl), 1.51 (s br, 3H, Me cumyl), 1.09 (s br, 3H, H2 or H2').

^{13}C NMR (400 MHz, C_7D_8): δ 130.0 (C6 or C6'), 129.5 (C6 or C6'), 129.4 (C4 or C4'), 128.9 (C4 or C4'), 127.7 (Co Ph cumyl), 63.9 (C3 or C3'), 61.3 (C3 or C3'), 57.0 (C1 or C1'), 43.8 (C2 or C2'), 38.4 (C2 or C2'), 32.2, 30.8, 29.8, 28.1 (C Me cumyl), 20.5, 20.4 (C5 or C5').

^{19}F NMR (400 MHz, C_7D_8): δ -131.8 (d br, 8F, o-F), -161.6 (t, JpF-mF = 21.3 Hz, 4F, pF), -165.6 (m, 8F, mF).

^{1}H NMR (400 MHz, C_6D_5Cl, 233K) δ 4.41 (broad, 1H, H3), 2.75 (broad, 2H, Hab), 2.66 (m broad, 2H, H3' + H1), 2.36 (s broad, 4H, H5' + H1'), 2.23 (s broad, 4H, H5 + H3), 2.05 (s broad, H3' + H2), 1.84 (s broad, 3H, Me cumyl), 1.58 (s broad, 3H, Me cumyl), 1.46 (m, 6H, Me cumyl + H1), 1.14 (d broad, 1H, H1'), 0.80 (s broad, 3H, H2').

^{13}C NMR (400 MHz, C_6D_5Cl, 233K) δ 72.4 (Cab), 62.6 (C3), 56.9 (C3'), 54.7 (C1), 51.0 (C1'), 48.3 (C2), 44.7 (C2'), 36.3, 35.2, 27.7, 26.6 (Me cumyl), 20.7 (C5, C5')

The concentration in chlorobenzene was too low to completely characterize the compound.

6^+. ^{1}H NMR (400 MHz, C_7D_8, 268K) δ 3.25, 3.09 (d, J = 15.1 Hz, H3), 2.75 (d br, H3'), 2.55 (br, H1'), 2.44 (t, J = 15.1 Hz, H1'), 2.33 (br, *Me*Ph_2), 2.11 (s buried, H5 + *Me*Ph_2), 1.94 (s br, H5'), 2.04 (m, H1' + H2), 1.29 (s br, 3H, H2').

^{13}C NMR (400 MHz, C_6D_6) δ 64.1 (C3), 61.3 (C3'), 56.9 (C1), 54.2 (C1'), 43.7 (C2), 38.6 (C2'), 29.3, 27.9 (*Me*Ph_2), 21.0 (C5), 20.1 (C5').

^{19}F NMR (400 MHz, C_7D_8): δ -131.8 (br, 8F, o-F), -161.5 (t, JpF-mF = 21.2 Hz, 4F, pF), -165.6 (m, 8F, mF).

^{1}H NMR (400 MHz, C_6D_5Cl) δ 6.99 (m, Hp), 6.88 (m, Hm'), 6.63 (m, Ho'), 6.52 (m, H6), 6.43 (broad, 1H, Hm), 6.14 (s broad, 1H, H4), 5.44 (broad, 1H, Ho), 4.50 (d broad, 1H, H3), 3.04 (d broad, 1H, H3), 2.87 (broad, 2H, Ha/b), 2.76 (m, 2H, H1' + H3'), 2.54 (m, 4H, PhMe_2 + H1), 2.38 (s broad, 3H, H2), 2.00 (m, 6H, H5' + PhMe_2), 1.85 (s broad, 3H, H5), 1.18 (d broad, 1H, H1), 0.91 (s broad, 3H, H2')

^{13}C NMR (400 MHz, C_6D_5Cl) δ 135.1 (C6'), 132.6 (Co), 131.3 (C4'), 130.1 (Cm), 129.8 (C4), 129.6 (Cp), 129.2 (C6), 128.3 (Cm'), 127.5 (Co'), 72.4 (Cab), 66.2 (C3), 62.6 (C3'), 54.6 (C1'), 50.9 (C1), 48.8 (C2), 44.7 (C2'), 27.5, 27.2 (C PhMe_2), 20.4 (C5, C5')

^{19}F NMR (400 MHz, C_7D_8): δ -131.8 (br, 8F, o-F), -161.3 (t, JpF-mF = 18.9 Hz, 4F, pF), -165.3 (m, 8F, mF).

7. ^{1}H NMR (400 MHz, CD_2Cl_2, 233K) δ 4.05 (d, J = 13.0 Hz, 1H, H3 species 1), 3.09 (d, J = 14.1 Hz, 1H, H3 species 2), 2.73 (m, 3H, H3 species 1 + H3 species 3), 2.50 (m, 2H, H3 species 3 + Ha/b species 1/2/3), 2.37 (s, 3H, H5 species 1/2/3), 2.31 (m, H3 species 2 + residual toluene), 2.26 (m, 4H, H5 species 1/2/3 + H1 species 1/2/3), 2.11 (s, 3H, H2 species 1/2/3), 2.04 (s, 3H, Me Fluorenyl species 1/2/3), 1.75 (m, 4H, H5 species 1/2/3 + Ha/b species 1/2/3), 1.71 (s, 3H, Me Fluorenyl species 1/2/3), 1.50 (m, 5H, Me Fluorenyl species 1/2/3 + H1 species 1/2/3 + Ha/b species 1/2/3), 1.39 (d, J = 10.9 Hz, 1H, Ha/b species 1/2/3), 1.21 (m, H1 species 1/2/3), 1.17 (m, 4H, H2 species 1/2/3 + Ha/b species 1/2/3), 0.77 (s, 3H, H2 species 1/2/3), 0.57 (d, J = 11.4 Hz, Ha/b species 1/2/3)
^{13}C NMR (400 MHz, C_6D_6) δ 73.0, 72.1, 71.0 (Cab species 1/2/3), 63.0, 62.0, 61.9 (C3 species 1/2/3), 52.2, 52.0, 51.3 (C1 species 1/2/3), 45.5, 44.0, 43.7 (C2 species 1/2/3), 32.9, 31.9, 27.9 (Me Fluorenyl species 1/2/3), 20.8, 20.5, 20.1 (C2 species 1/2/3).

7^{+}. ^{1}H NMR (400 MHz, C_7D_8) δ 2.45 (s buried, H5'), 2.17 (s buried, Me fluorenyl), 1.93 (s broad, H2), 1.59 (s buried, H5), 1.39 (broad, Me fluorenyl), 1.24 (m, H2' + pentane).
^{13}C NMR (400 MHz, C_7D_8) δ 42.5 (C2), 39.0 (C2'), 31.5, 27.4 (Me fluorenyl), 20.4 (C5'), 19.6 (C5).
^{19}F NMR (400 MHz, C_7D_8): δ -131.7 (s broad, 8F, o-F), -161.5 (t broad, 4F, pF), -165.6 (m, 8F, mF).
^{1}H NMR (400 MHz, C_6D_5Cl) δ 2.74 (br, H3 or H3'), 2.46 (br, H1'), 2.41 (s br, 3H, H5'), 1.64 (br, H5), 1.47, 1.45 (br, Me fluorenyl), 1.14 (br, 4H, H2' + H1').
^{13}C NMR (400 MHz, C_6D_5Cl) δ 63.3 (C3 or C3'), 51.2 (C1), 46.0 (C2, C2'), 34.5, 31.7 (Me fluorenyl), 20.7 (C5'), 20.0 (C5).
^{19}F NMR (400 MHz, C_7D_8): δ -131.7 (s broad, 8F, o-F), -161.8 (t, J pF-mF = 21.6 Hz, 4F, pF), -165.7 (m, 8F, mF). The NMR spectrum in chlorobenzene was not clear enough to allow for a complete characterization of the ion pair.

NMR spectra of activated complexes

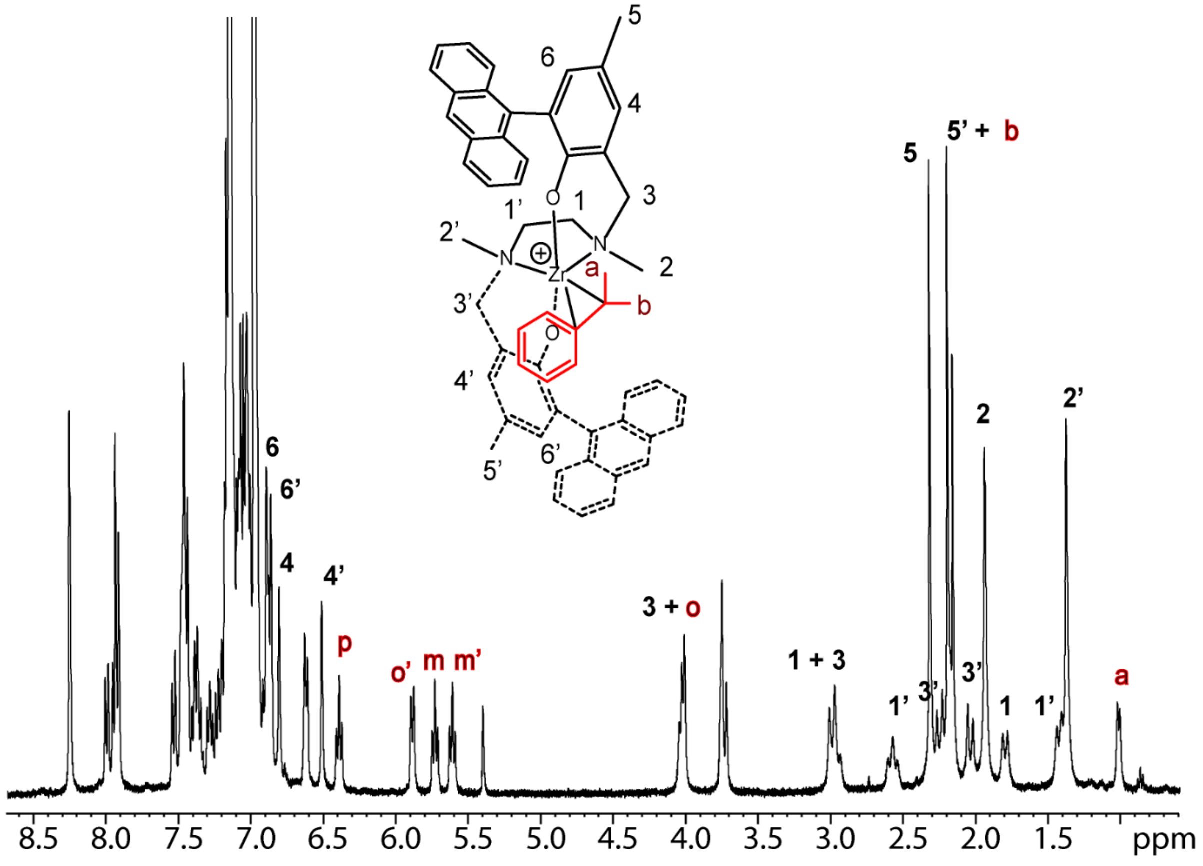

Figure S17. **^{1}H NMR spectrum (233K, C_6D_5Cl) of 3^+_{FF}.**

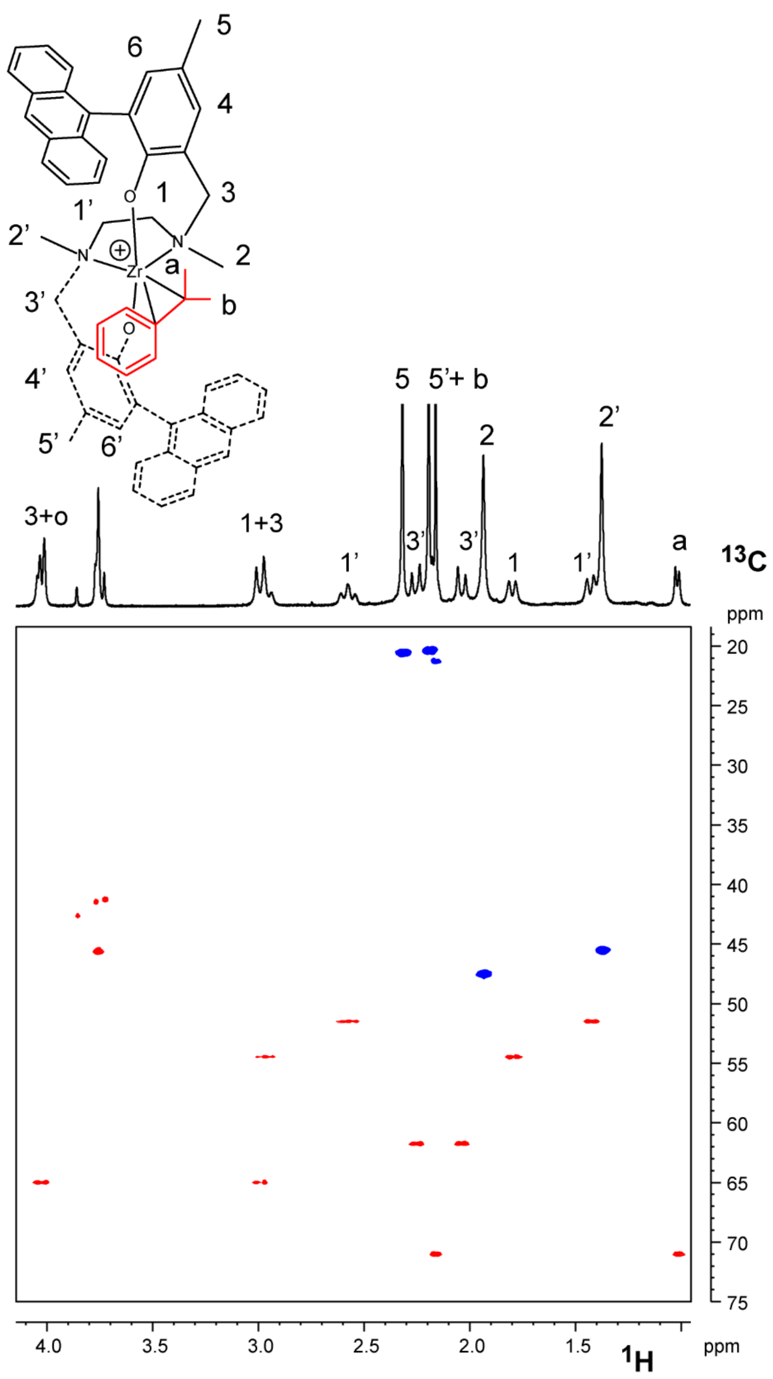

Figure S18. $^{1}H,^{13}C$ HSQC NMR spectrum (233K, C_6D_5Cl) of $\mathbf{3^{+}_{FF}}$.

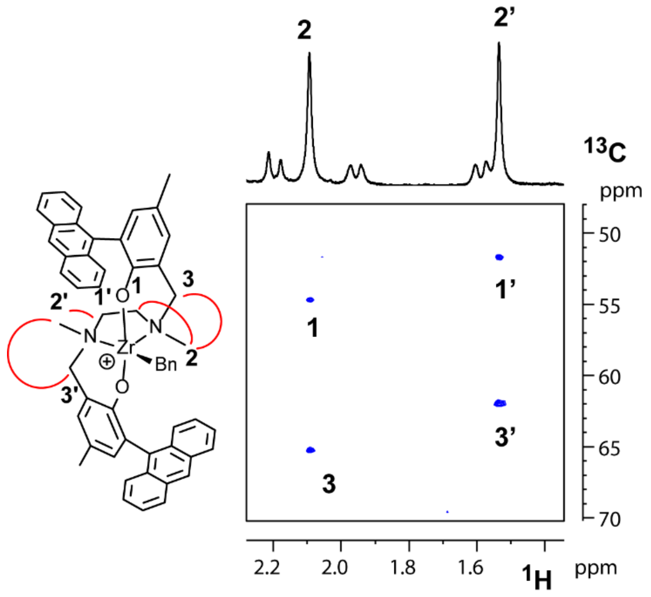

Figure S19. $^{1}H,^{13}C$ HMBC NMR spectrum (233K, C_6D_5Cl) of $\mathbf{3^{+}_{FF}}$.

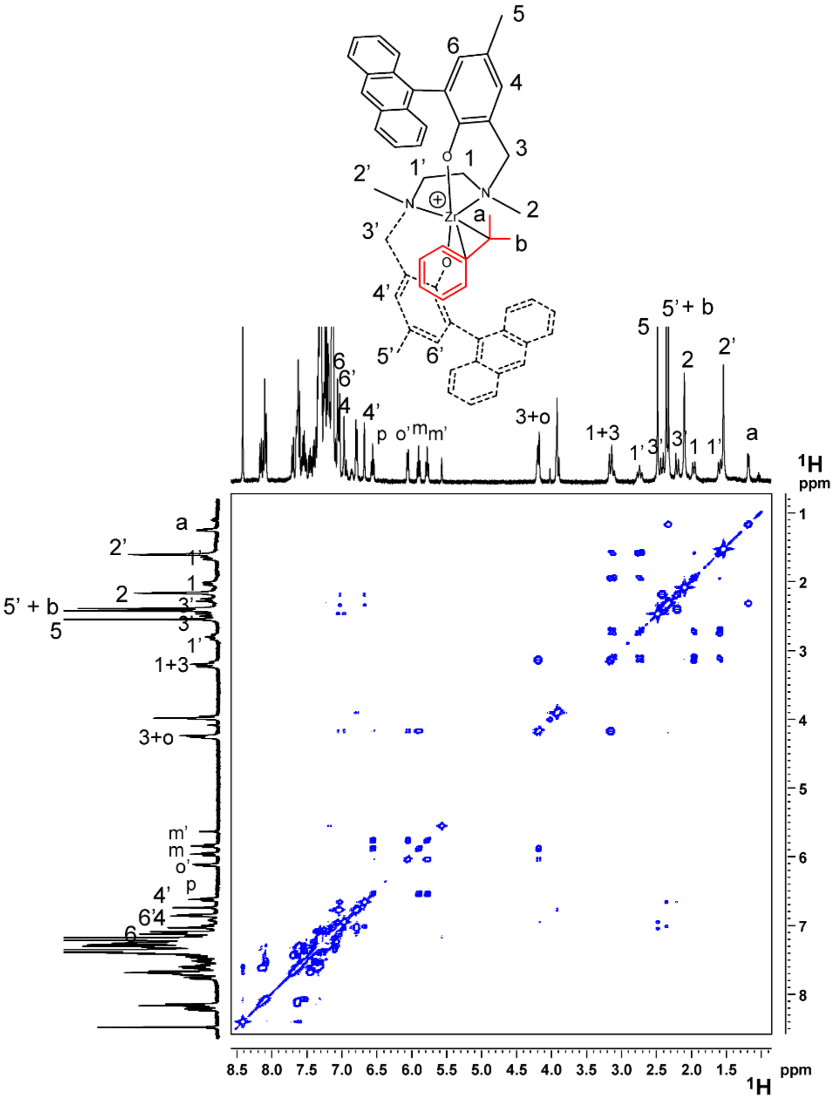

Figure S20. $^{1}H,^{1}H$ COSY NMR spectrum (233K, C_6D_5Cl) of $\mathbf{3^{+}_{FF}}$.

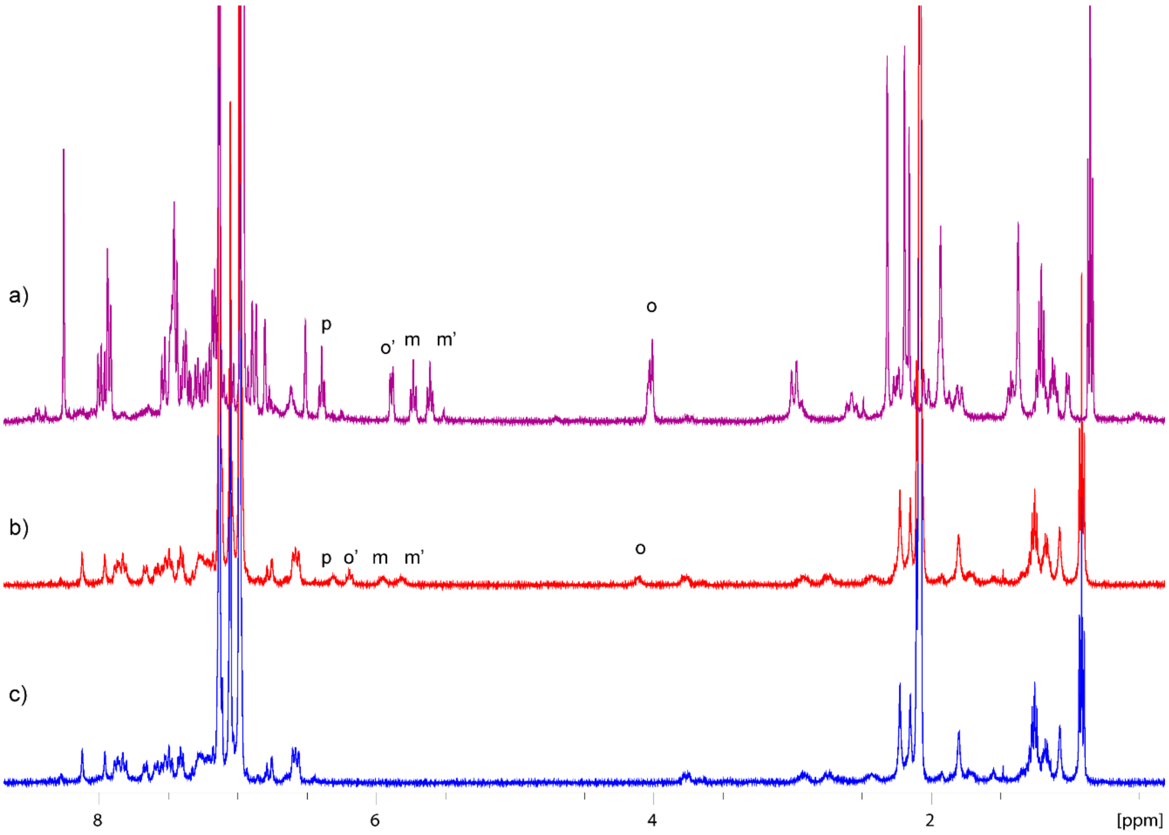

Figure S21. Stacked ^{1}H NMR spectra (233K) of: a) **3**$^{+}$ synthesized in C_7H_8 and precipitated from *n*-pentane, redissolved in C_6D_5Cl; b) **3**$^{+}$ synthesized in C_7H_8 and precipitated from *n*-pentane, redissolved in C_7D_8; c) spectrum b) recorded after several minutes.

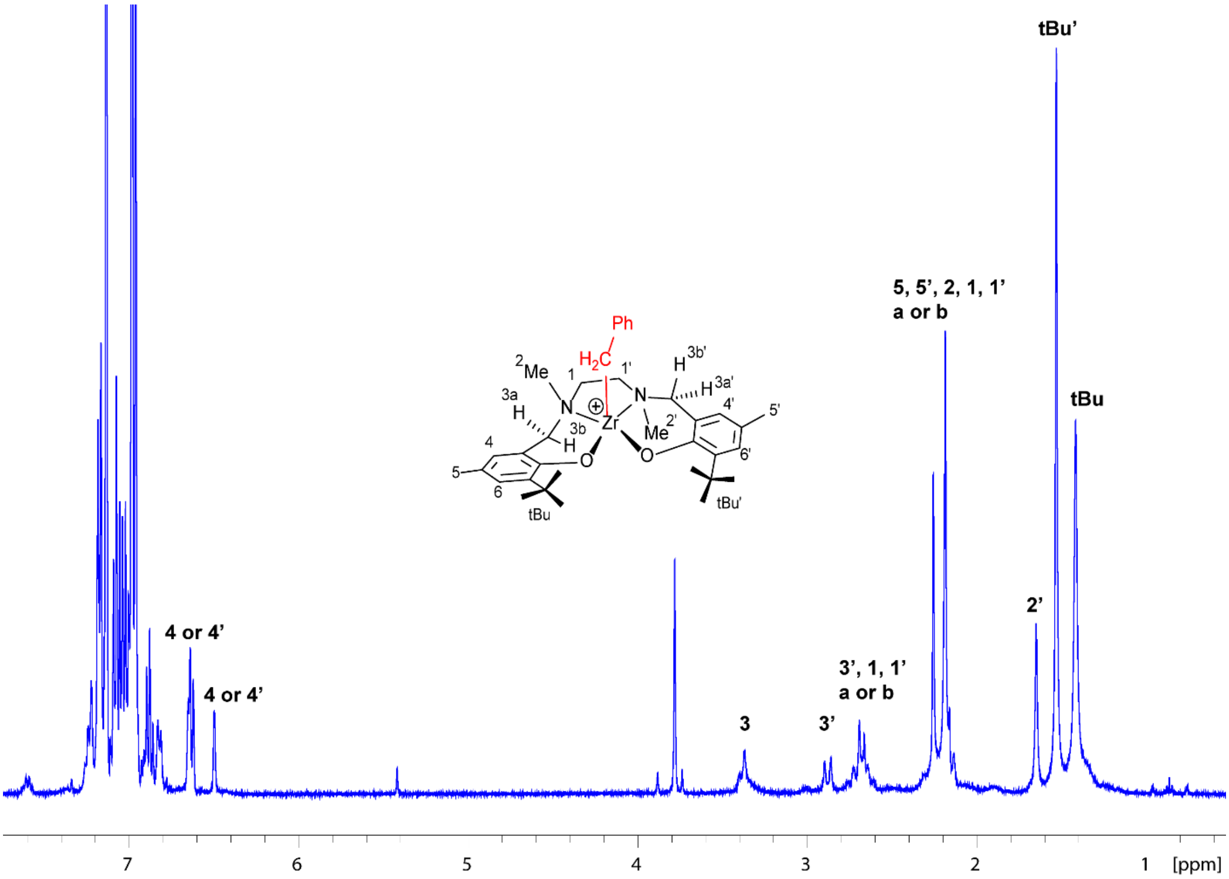

Figure S22. ^{1}H NMR spectrum (253K) of $\mathbf{4^+_{MM}}$ in C_6D_5Cl.

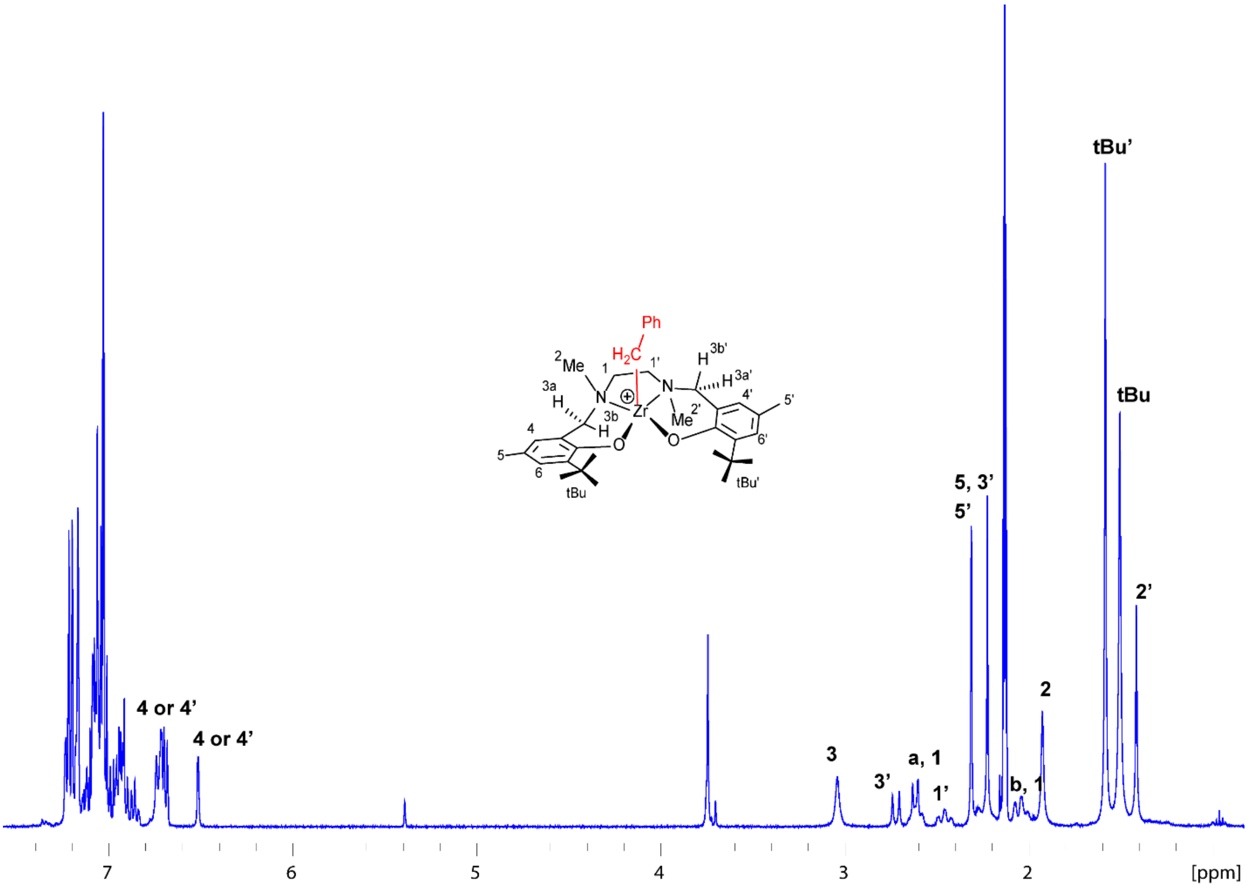

Figure S23. ^{1}H NMR spectrum (253K) of $\mathbf{4^+_{MM}}$ in C_7D_8.

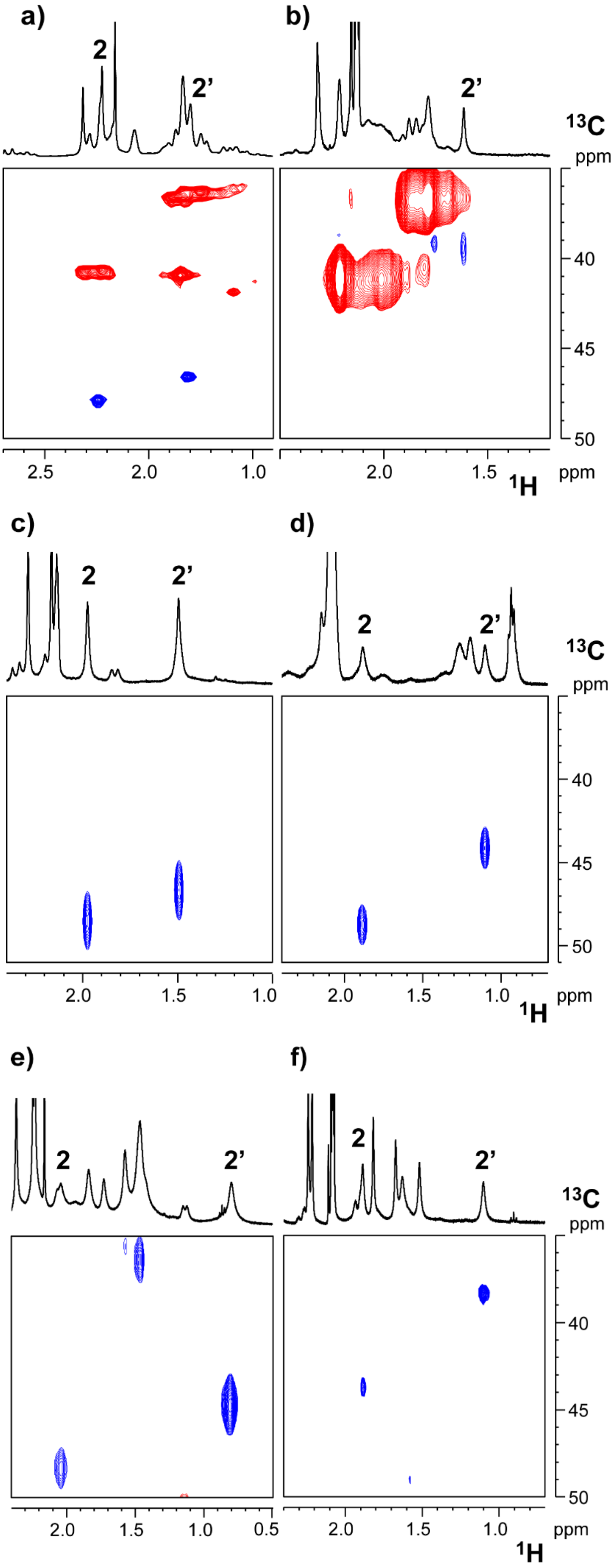
a)
b)
2
2'
2'
13C
ppm
40
45
50
2.5
2.0
1.0
2.0
1.5
1H
ppm
c)
d)
2
2'
2
2'
13C
ppm
40
45
50
2.0
1.5
1.0
2.0
1.5
1.0
ppm
1H
e)
f)
2
2'
2
2'
13C
ppm
40
45
50
2.0
1.5
1.0
0.5
2.0
1.5
1.0
ppm
1H

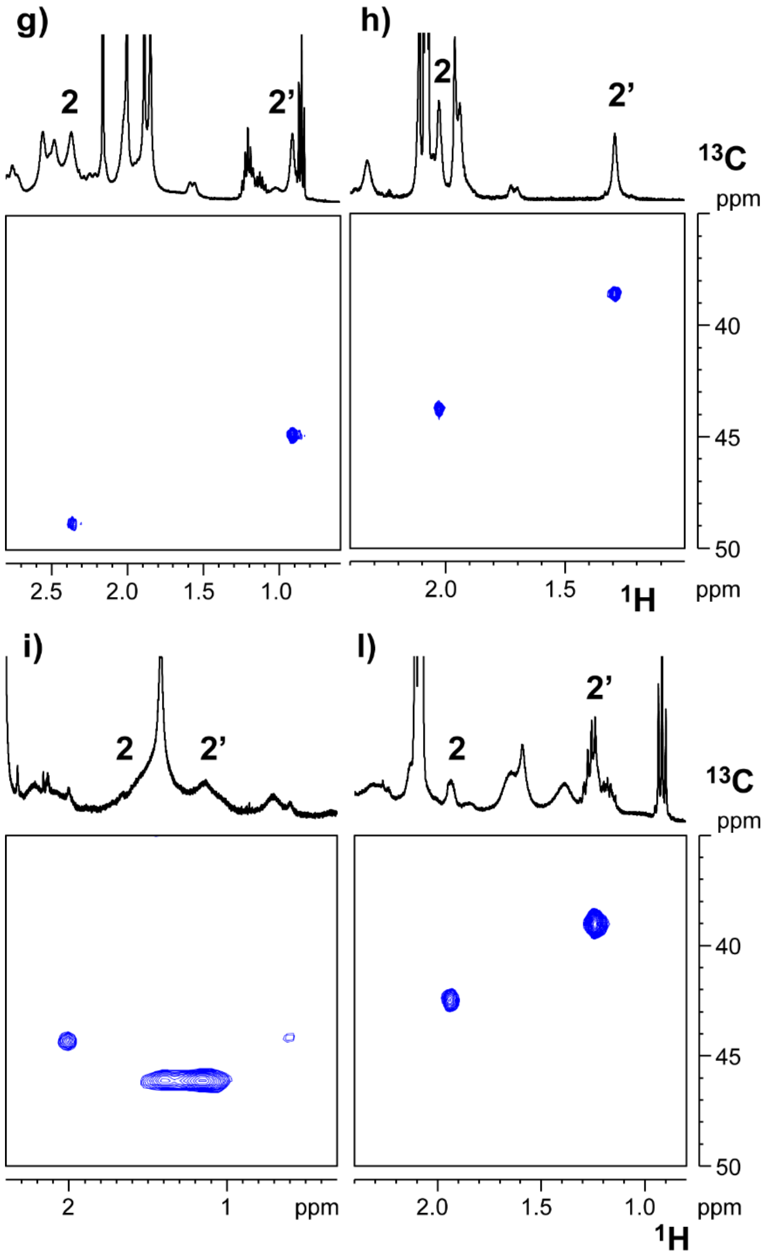

Figure S24. Sections of $^{1}H,^{13}C$ HSQC NMR spectra, region of NMe groups (35 – 50 ppm), of: a) $\mathbf{1^{+}}_{FF}$ (C_6D_5Cl, 233K), b) $\mathbf{1^{+}}_{MM}$ (C_7D_8, 253K), c) $\mathbf{2^{+}}_{FF}$ (C_6D_5Cl, 233K), d) $\mathbf{2^{+}}_{FF}$ (C_7D_8, 233K), e) $\mathbf{5^{+}}_{FF}$ (C_6D_5Cl, 233K), f) $\mathbf{5^{+}}_{MM}$ (C_7D_8, 268K), g) $\mathbf{6^{+}}_{FF}$ (C_6D_5Cl, 233K), h) $\mathbf{6^{+}}_{MM}$ (C_7D_8, 268K), i) $\mathbf{7^{+}}_{FF}$ (C_6D_5Cl, 233K), l) $\mathbf{7^{+}}_{FF}$ (C_7D_8, 233K).

Reactivity of *3^{+}_{FF} with 1-hexene

To a solution in C_6D_5Cl of 10 mg of $\mathbf{3^{+}}_{FF}$ and 0.95 eq of TrBT, 30 eq of 1-hexene have been added, and the reaction followed with *in situ* NMR over the course of some minutes in order to track the consumption of the starting material. The formation of poly-1-hexene and a new organometallic species was evidenced. The newly formed organometallic species, as explained in the main text, retains the *fac-fac* geometry of $\mathbf{3^{+}}_{FF}$. It is proposed that such species is indeed the 1-hexene

monoinserted species into the Zr-benzyl bond. The main NMR evidences that point out to this species are the following: the $^{1}H,^{13}C$ HSQC NMR shows two two doublets (-0.72, 0.21 ppm) with a carbon resonating at 71.1 ppm, that are assigned to a Zr-*CH_2* moiety. Following the $^{1}H,^{1}H$ COSY correlation, these signals show coupling with a signal at 0.09 ppm, that in turn has the relevant carbon in the positive phase at 54.5 ppm. This is thus assigned to the Zr-CH_2-*CH*. The latter shows correlations, both in the $^{1}H,^{1}H$ COSY and in the $^{1}H,^{1}H$ ROESY with two signals at 0.38 and 0.63 ppm, that shares the carbon at 41.9 ppm, that is compatible with a Zr-CH_2-CH(R)-*CH_2*Ph. The rest of chain of the monoinserted species is difficult to assign, since its signals fall under the poly-1-hexene signal, however the $^{1}H,^{1}H$ ROESY spectrum allows to individuate a CH_2 at 0.80 ppm and another one at 1.86 ppm that shows NOE contacts with the CH of the inserted monomer. Moreover, the Zr-CH_2, in the ROESY spectrum, shows NOE contacts with the signals that are typically assigned to the H1 and H3 of the ligand framework, and a small NOE contact of the signal at 0.21 ppm with the CH_2Ph further corroborates the formation of the monoinserted species.

***3^{+}_{FF}-$CH_2CH(C_4H_9)CH_2Ph$:** ^{1}H NMR (400 MHz, 233K, C_6D_5Cl) δ -0.71 (d, 1H, Zr-*CH_2*), 0.08 (br, 1H, Zr-CH_2*CH*), 0.21 (t, 1H, Zr-*CH_2*), 0.37 (m br, Zr-CH_2CH*CH_2*R), 0.65 (m br, Zr-CH_2CH*CH_2*R), 1.17 (buried, Zr-CH_2CH(R)*CH_2*Ph), 1.29 (buried, H1), 1.31 (buried, H2'), 1.38 (buried, H1'), 1.40 (buried, H2), 1.84 (buried, Zr-CH_2CH(R)*CH_2*Ph), 2.40 (d, 1H, H3), 2.50 (t, 1H, H1), 2.77 (m, 2H, H3'+H1'), 3.26 (d, 1H, H3'), 3.84 (t, 1H, m-Ph), 5.22 (d, 1H, o-Ph), 5.75 (d, 1H, o'-Ph), 5.97 (t, 1H, p-Ph), 6.53 (t, 1H, m'-Ph).

^{13}C NMR (400 MHz, 233K, C_6D_5Cl) δ 40.5 (Zr-CH_2CH(R)*CH_2*Ph), 42.0 (Zr-CH_2CH*CH_2*R), 45.6 (C2'), 47.0 (C2), 50.5 (C1), 55.0 (C1'), 63.1 (C3), 63.3 (C3'), 71.1 (Zr-*CH_2*), 125.9 (Cp), 130.4 (Co), 131.3 (Cm), 131.4 (Cm'), 132.8 (Co').

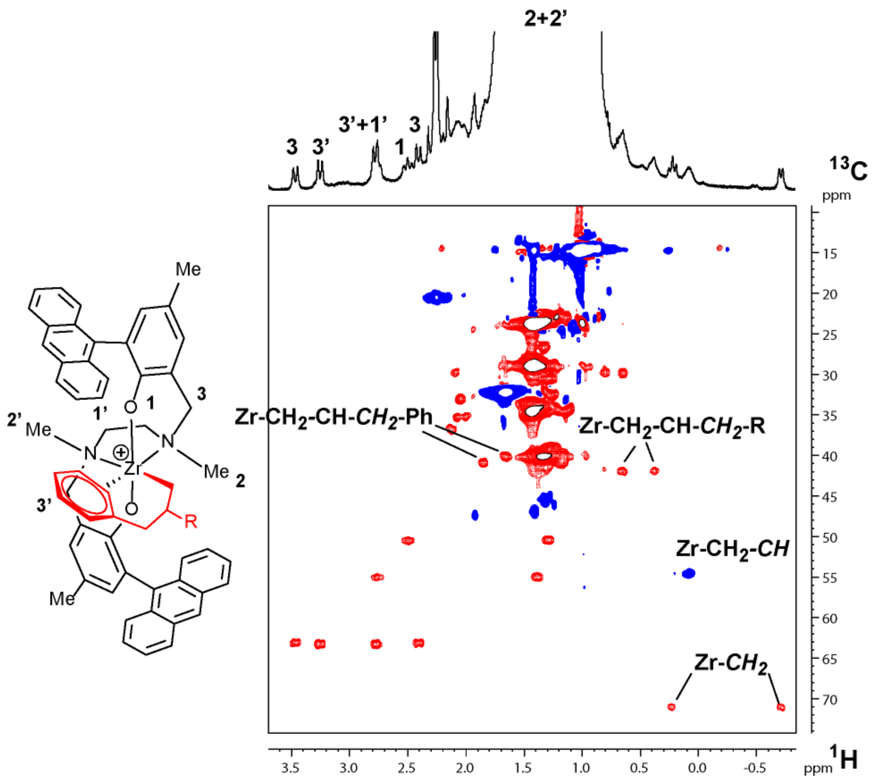

Figure S25. Aliphatic region of the $^1H,^{13}C$ HSQC NMR spectrum of the reaction between $\mathbf{3^+}_{\mathbf{FF}}$ and 1-hexene (233K, C_6D_5Cl). R on the CH fragment omitted for clarity.

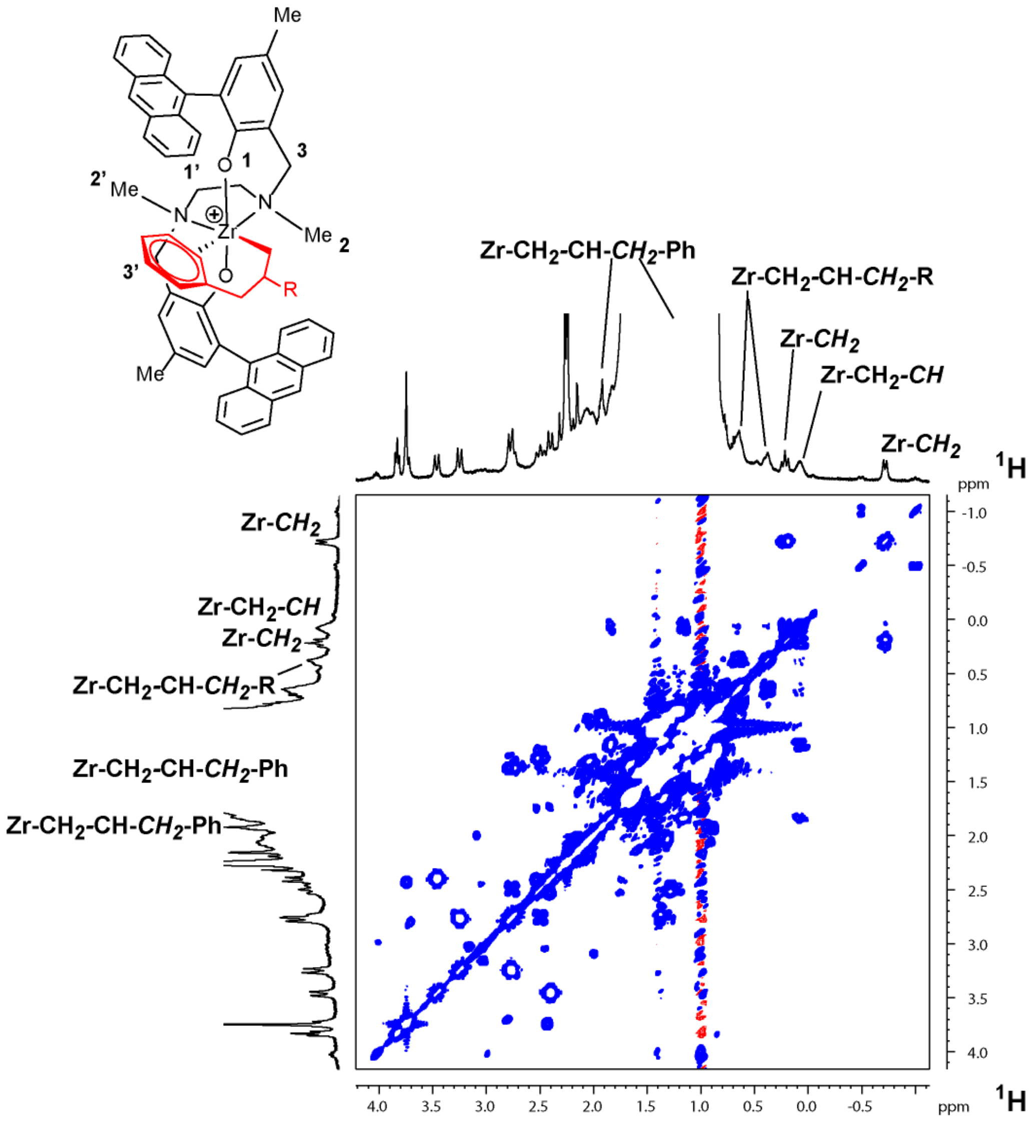

Figure S26. Aliphatic region of the ^{1}H,^{1}H COSY NMR spectrum of the reaction between **3**$^{+}_{FF}$ and 1-hexene (233K, C_6D_5Cl). R on the CH fragment omitted for clarity.

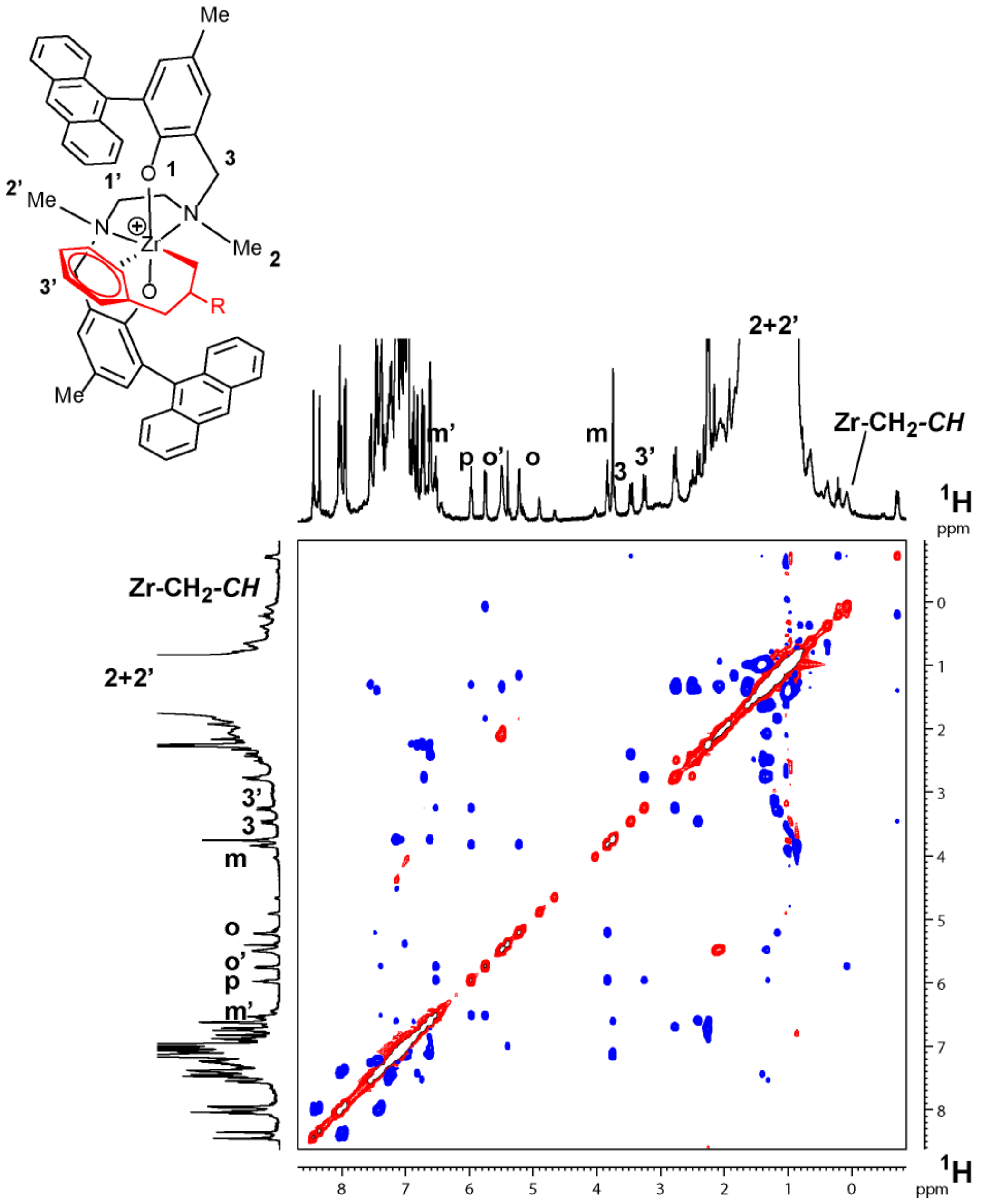

Figure S27. $^1H,^1H$ ROESY NMR spectrum of the reaction between $\mathbf{3^+_{FF}}$ and 1-hexene (233K, C_6D_5Cl). R on the CH fragment omitted for clarity.

Reactivity of $\mathbf{*4H^+_{MM}}$ with 1-hexene

To a solution in C_6D_5Cl of 9.5 mg of $\mathbf{*4H^+_{MM}}$ and 0.95 eq of TrBT, 30 eq of 1-hexene have been added, and the reaction followed with *in situ* NMR over the course of some minutes in order to highlight the disappearance of the starting material. The formation of poly-1-hexene and a new organometallic species was evidenced. The newly formed organometallic species, as explained in the main text, retains the *mer-mer* geometry of $\mathbf{*4H^+_{MM}}$. It is proposed that such species is in this case

the polymeryl species. The complete characterization of this species it's a bit more complex with respect to the relevant **3^+_{FF}** derivative, however there is NMR evidence that points out to **$*4H^+$-P_n**: from the ^{1}H,^{13}C HSQC it is possible to recognize two different set of signals at 1.01, 1.46 ppm (with the relevant carbon at 43.1 ppm) and at 2.49, 2.66 ppm (with the relevant carbon at 40.9 ppm). These two sets of signals can be assigned to a Zr-*CH_2* or a Zr-($CH_2CH(C_4H_9)_n$-*CH_2*. The difference in chemical shift of the Zr-*CH_2* group with respect to the previously analysed **$*3^+_{FF}$-$CH_2CH(C_4H_9)CH_2Ph$** could be ascribed to the different geometry of the two complexes and to the absence of an interaction of the terminal phenyl group, due to the presence of a longer chain, that prevents the stiffening that is present in the monoinserted derivative. Other signals belonging to the chain that could be easily recognized are those at 1.83 ppm (δ_C: 37.8 ppm) and at 2.24 ppm (δ_C: 43.7 ppm), that are instead assigned to Zr-CH_2*CH* or Zr-(polymeryl)-CH_2*CH*. All the signals aforementioned show in the ^{1}H,^{1}H ROESY spectrum contacts with other signals in the aliphatic region, likely due to the C_4H_9 chains of the growing polymeryl; it is however rather difficult to precisely assign these signals due to the presence of the poly-1-hexene signal. The fragments relevant to the ligand backbone can be easily recognized from both the ^{1}H,^{13}C HSQC, the ^{1}H,^{1}H ROESY and ^{1}H,^{1}H COSY NMR spectra.

$*4H^+_{MM}$-polymeryl: ^{1}H NMR (400 MHz, 253K, C_6D_5Cl) δ 1.01 (buried, Zr-*CH_2*), 1.46 (buried, Zr-*CH_2*), 1.83 (br, Zr-CH_2*CH* or Zr-(polymeryl)-CH_2*CH*), 2.24 (br, Zr-CH_2*CH* or Zr-(polymeryl)-CH_2*CH*), 1.77 (s, 3H, H2'), 2.27 (s, 3H, H2), 2.36-2.54 (m, 3H, H1 + H1' + Zr-(polymeryl)-*CH_2*CH), 2.66 (dd, 1H, Zr-(polymeryl)-*CH_2*CH), 2.78 (t, 1H, H1'), 3.00 (t, 1H, H1), 3.33 (d, 1H, H3), 3.47 (d, 2H, H3+H3'), 4.32 (d, 1H, H3').

^{13}C NMR (400 MHz, 233K, C_6D_5Cl) δ 43.1 (Zr-*CH_2*), 37.0, 38.0 (Zr-CH_2*CH* or Zr-(polymeryl)-CH_2*CH*), 37.8 (C2'), 40.9 (Zr-(polymeryl)-*CH_2*CH), 43.7 (C2), 54.9 (C1'), 56.4 (C1), 60.4 (C3'), 60.8 (C3).

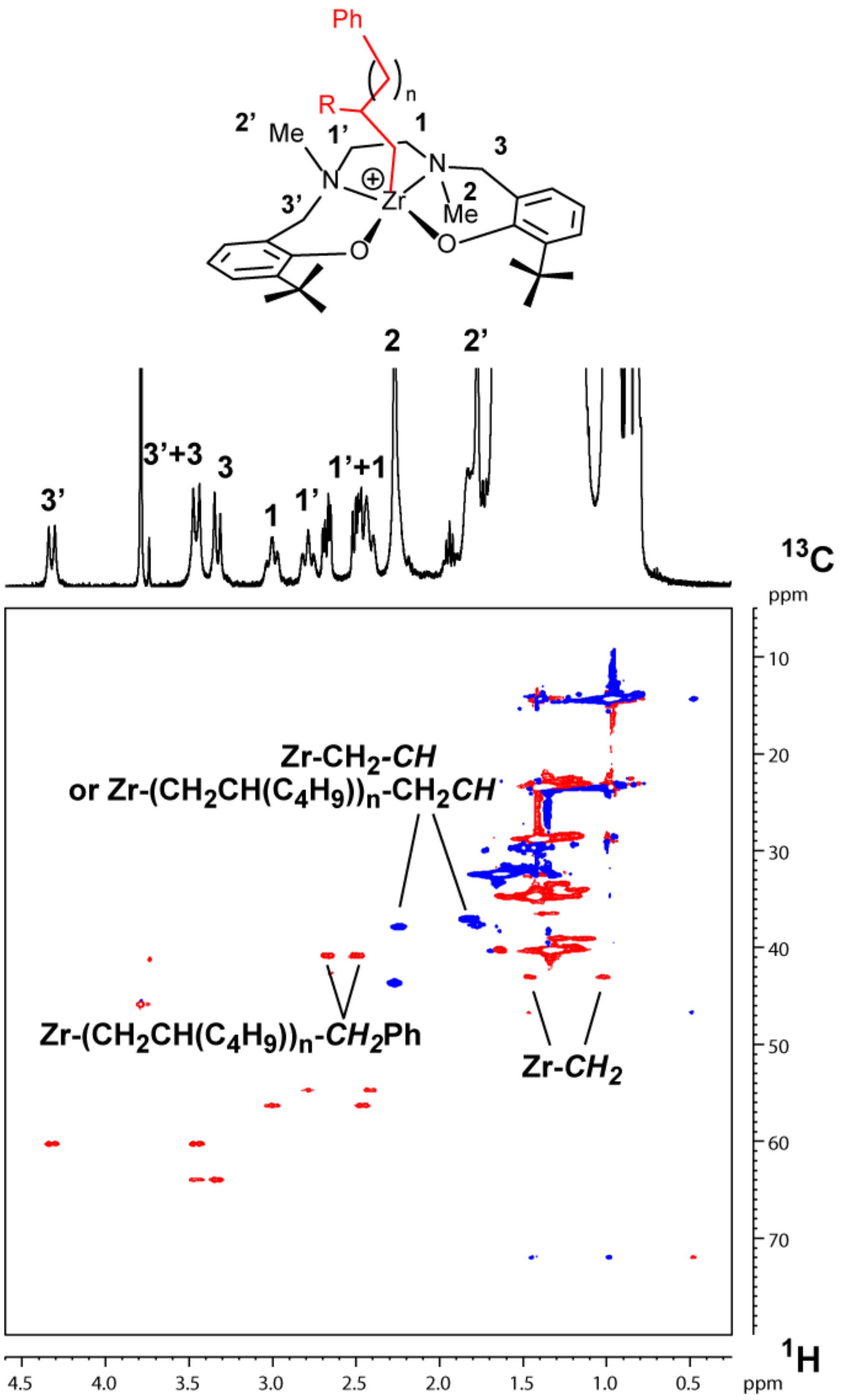

Figure S28. ^{1}H,^{13}C HSQC NMR spectrum of **$4H^{+}_{MM}$** with 1-hexene (233K, C_6D_5Cl), aliphatic region.

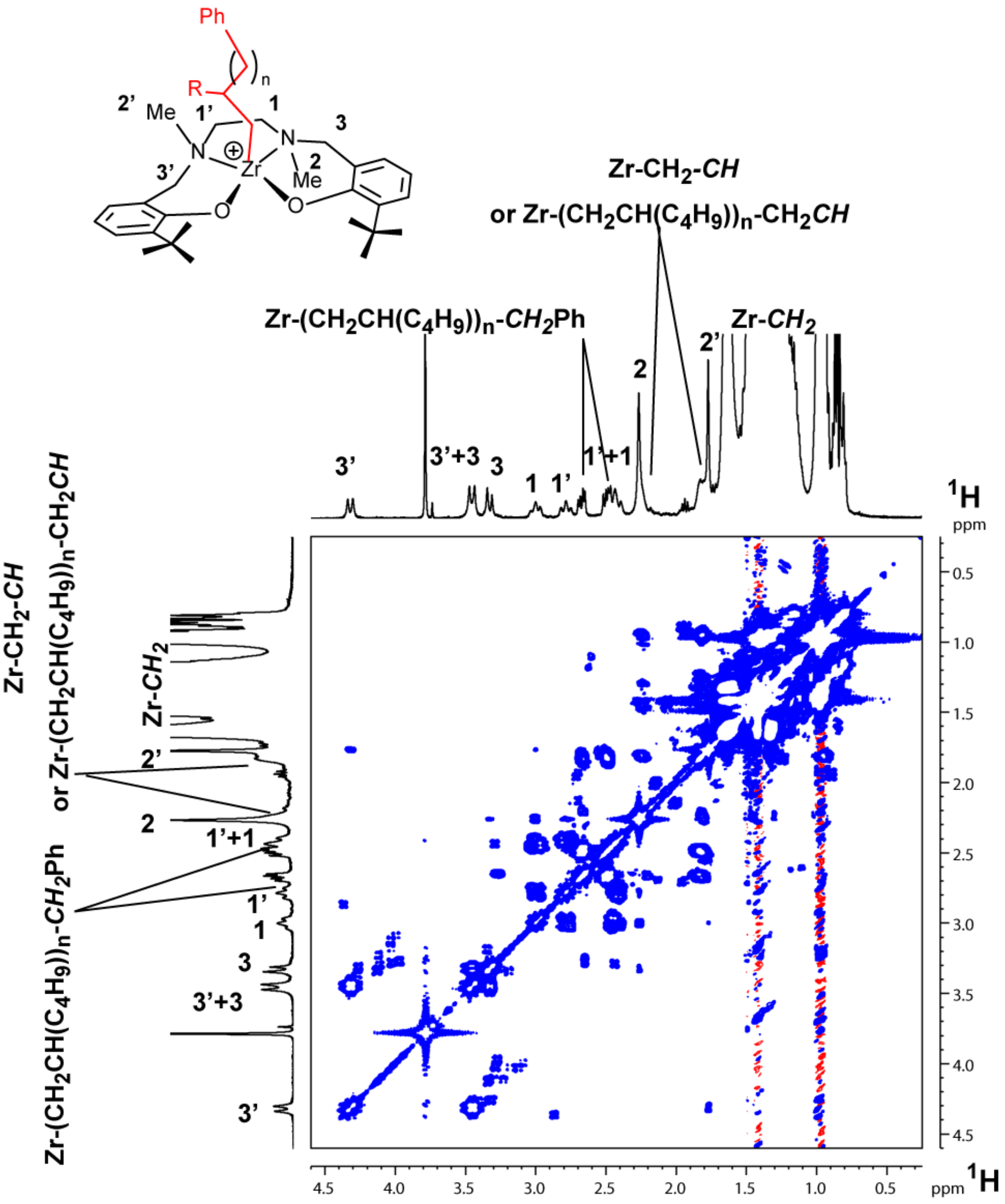

Figure S29. ^1H,^1H COSY NMR spectrum of **$4H^+_{MM}$** with 1-hexene (233K, C_6D_5Cl), aliphatic region.

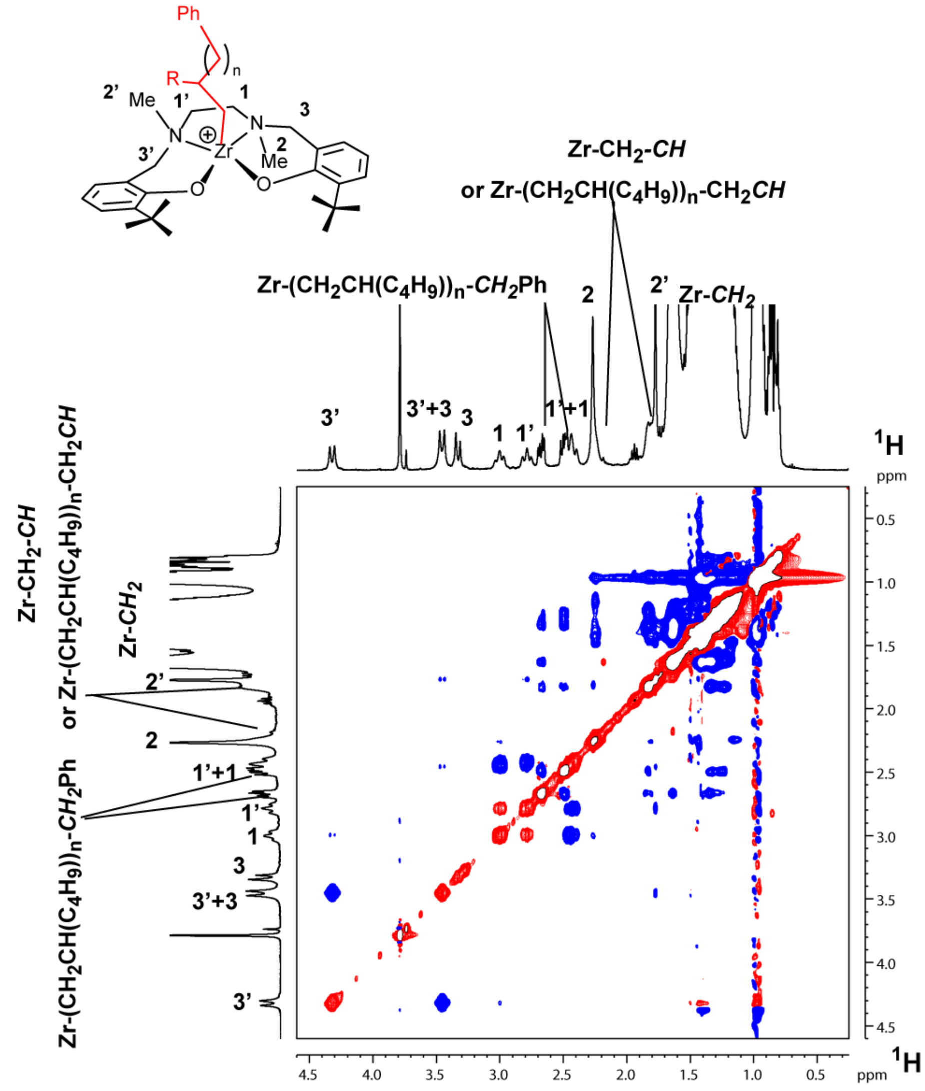

Figure S30. $^{1}H,^{1}H$ ROESY NMR spectrum of **$4H^{+}_{MM}$** with 1-hexene (233K, C_6D_5Cl), aliphatic region.

Table S1. ^{13}C NMR chemical shifts of C2 and C2' (δC2 and δC2', externally referenced to TMS in ppm) for complexes **1**$^+$ - **7**$^+$ and **8**$^+$.

Entry	Complex	S	δ C2	δ C2'	T	Geom	Entry	Complex	S	δ C2	δ C2'	T	Geom
1	1	C_6D_6	45.2	45.2	298	FF	16	*4H$^+$·Lut	C_6D_5Cl	47.4	47.2	298	FF
2	1$^+$	C_7D_8	-	39.3	253	MM	17	*4H$^+$·THF	C_6D_5Cl	47.3	47.2	292	FF
3	1$^+$	C_6D_5Cl	48.0	46.6	233	FF	18	5	C_6D_6	44.4	44.4	298	FF
4	2	C_6D_6	45.2	45.2	298	FF	19	5$^+$	C_7D_8	43.7	38.4	268	MM
5	2$^+$	C_7D_8	48.7	44.1	233	FF	20	5$^+$	C_6D_5Cl	48.3	44.7	233	FF
6	2$^+$	C_6D_5Cl	47.7	45.9	233	FF	21	5$^+$	C_6D_5Cl	48.7	44.5	223	FF
7	3	C_6D_6	45.2	45.2	298	FF	22	5+	C_6D_5Cl	46.0<br>45.1<br>44.3	39.0<br>38.6<br>39.1	298<br>298<br>298	MM<br>MM<br>MM
8	3$^+$	C_7D_8	48.5	44.5	233	FF	23	6	C_6D_6	44.6	44.6	298	FF
9	3$^+$	C_6D_5Cl	47.7	45.6	233	FF	24	6$^+$	C_7D_8	43.7	38.6	268	MM
10	3$^+$·Lut	C_6D_5Cl	47.0	45.2	298	FF	25	6$^+$	C_6D_5Cl	48.8	44.8	233	FF
11	3$^+$·THF	C_6D_5Cl	46.8	45.3	298	FF	26	6$^+$	C_6D_5Cl	44.9	39.3	273	MM
12	*4H	C_6D_6	46.3	46.3	298	FF	27	7	C_6D_6	44.0<br>45.4<br>45.9	43.5<br>45.4<br>45.9	298<br>298<br>298	FF<br>FF<br>FF
13	4$^+$	C_7D_8	41.9	37.6	263	MM	28	7$^+$	C_7D_8	42.5	39.0	233	MM
14	*4H$^+$	C_6D_5Cl	47.3	47.3	233	FF	29	7$^+$	C_6D_5Cl	46.0	46.0	233	FF
15	*4H$^+$	C_6D_5Cl	42.9	38.4	263	MM	30	8$^+$	C_6D_5Cl	42.7	37.6	233	MM

Table S2. Site Epimerization rate constants (k_{SE}, s^{-1}) at different temperatures (T, K) and corresponding activation parameters ($\Delta H^{\neq}$, kcal·mol^{-1}; $\Delta S^{\neq}$, cal·mol^{-1}·K^{-1}; $\Delta G^{\neq}_{(298)}$, kcal·mol^{-1}; $\Delta G^{\neq}_{(333)}$, kcal·mol^{-1})[a] for the Ion Pair Symmetrisation process of **3**$^+_{FF}$**, 4**$^+_{MM}$ and ***4H**$^+_{MM}$ in C_7D_8.

T (K)	K_{SE}	$\Delta H^{\neq}$	$\Delta S^{\neq}$	$\Delta G^{\neq}_{(298)}$	$\Delta G^{\neq}_{(333)}$
	**3**$^+_{FF}$				
218	0.34	15.2 ± 0.6	10 ± 2	12.3 ± 0.9	11.9 ± 1.0
223	0.74				
233	4.32				
238	7.43				
243	17.21				
248	22.48				
250	34.53				
253	59.86				
258	139.66				
263	199.64				
268	442.74				
288	2579.02				
293	2913.95				
298	4087.44				
303	12251.10				

313	25907.14				
	**4^{+}_{MM}**				
243	0.020	14.0 ± 2.1	-8 ± 4	16.4 ± 1.5	16.7 ± 1.6
253	0.077				
263	0.272				
273	0.525				
283	2.004				
293	4.559				
303	8.167				
313	16.807				
	**$*4H^{+}_{MM}$**				
243	0.09	11.4 ± 0.9	-16.4 ± 3.3	16.3 ± 1.3	16.9 ± 1.4
253	0.16				
258	0.28				
263	0.44				
268	0.63				
273	1.00				
278	1.81				
283	2.43				
288	3.48				
293	5.17				

[a] computed from the slopes and intercepts of the corresponding Eyring plots; errors determined from the quality of linear fitting and computed at 95% confidence interval.

Table S3. Site Epimerization rate constants (k_{SE}, s^{-1}) at different temperatures (T, K) and corresponding activation parameters ($\Delta H^{\neq}$, $kcal \cdot mol^{-1}$; $\Delta S^{\neq}$, $cal \cdot mol^{-1} \cdot K^{-1}$; $\Delta G^{\neq}_{(298)}$, $kcal \cdot mol^{-1}$; $\Delta G^{\neq}_{(333)}$, $kcal \cdot mol^{-1}$)[a] for the Ion Pair Symmetrisation process of **3^{+}_{FF}** and **$*4H^{+}_{MM}$** in C_6D_5Cl.

T (K)	k_{IPS}	$\Delta H^{\neq}$	$\Delta S^{\neq}$	$\Delta G^{\neq}_{(298)}$	$\Delta G^{\neq}_{(333)}$
	**3^{+}_{FF}**				
253	0.10	17.3 ± 0.1	5.9 ± 1	15.5 ± 0.2	15.3 ± 0.2
258	0.23				
263	0.47				
268	0.91				
273	1.33				
278	4.21				
283	6.83				
288	8.76				
293	15.53				
298	17.80				
	**$*4H^{+}_{MM}$**				
243	0.38	12.2 ± 0.4	-9.8 ± 1.5	15.1 ± 0.6	15.5 ± 0.6
248	0.64				
253	1.03				
258	1.67				

263	3.09				
268	4.89				
273	7.17				
278	11.19				
283	14.35				
288	21.76				
293	27.63				
298	53.07				
303	73.88				
308	109.44				

[a] computed from the slopes and intercepts of the corresponding Eyring plots; errors determined from the quality of linear fitting and computed at 95% confidence interval.

X-Ray crystallography

Data collection of **3^{+}_{FF}·C_7D_8 and 3^{+}_{FF}·C_6D_5Cl** was performed at the University of Perugia on a Bruker D8 Venture X-ray diffractometer equipped with a low temperature device and Icoatec ImuS 3.0 microfocus sealed-tube MoKα (λ = 0.71073). Complete dataset was collected at 100 K with the CCD PHOTON II detector placed at a distance 40.00 mm from the crystal. The data collected through generic φ and ω scans were integrated and reduce using the Bruker AXS SAINT V8 software. The structure was solved and anisotropically refined using the SHELXT and SHELXL packages of the Olex2 software. Hydrogen atoms were placed at calculated positions and forced to ride on the attached atom. Crystal data and details of refinements are given in Table S4-5.

Table S4. X-ray diffraction data for complex $\mathbf{3^{+}_{FF}{\cdot}C_7D_8}$.

Elemental formula	C398.63 H290.43 B4 F80 N8 O8 Zr4
Formula weight	7248.46
Crystal system	Triclinic
Space group	P 1
Unit cell dimensions:<br>a = (Å)	 17.4078(17)
b =	17.9387(17)
c =	27.841(3)
α = (°)	77.636(2)
β =	82.163(2)
γ =	76.870(2)
Volume (^{3})	8235.1(14)
Z, Calculated density (g/cm^3)	1, 1.462
F(000)	3702.0
Absorption coefficient (mm^{-1})	0.233
Temperature (K)	100(2)
Crystal colour, shape	Yellow, Plate
Crystal size (mm)	0.200 x 0.120 x 0.060
On the diffractometer:	
Theta range for data collection	1.84 to 25.00
Limiting indices	-20<=h<=20, -21<=k<=21, -33<=l<=33
Completeness to theta = 30.52 (%)	99.9%
Max. and min. transmission	0.6886 and 0.7457
Reflns collected (not incl. absences)	261799
No of unique reflns, R(int) for equivs	57905, 0.0470
No. of ‘observed’ reflns ($I > 2\sigma_I$)	53435

Refinement:	
Data/restraints/parameters	57905/204/4595
Goodness-of-fit on F^2	1.041
Final R indices ('obsd' data)	R_1 = 0.0332, wR_2 = 0.0745
Final R indices (all data)	R_1 = 0.0400, wR_2 = 0.0788
Reflns weighted: 1/w = [a]	[\s^2^(Fo^2^)+(0.0345P)^2^+3.5029P]
Largest diff. peak and hole (e. Å $^{-3}$)	0.795 and -0.346

[a] where P=(Fo^2^+2Fc^2^)/3'

Table S5. X-ray diffraction data for complex $\mathbf{3^+_{FF}{\cdot}C_6D_5Cl}$.

Elemental formula	C191.95 H131 B2 Cl3.12 F40 N4 O4 Zr2
Formula weight	3632.05
Crystal system	Monoclinic
Space group	C 1 C 1
Unit cell dimensions:<br>a = (Å)	 22.0184(11)
b =	27.9463(14)
c =	27.0511(15)
α = (°)	90
β =	99.755(2)
γ =	90
Volume (^{3})	16404.8(15)
Z, Calculated density (g/cm^3)	4, 1.471
F(000)	7983
Absorption coefficient (mm^{-1})	0.283
Temperature (K)	100(2)
Crystal colour, shape	Clear light gold, Plate
Crystal size (mm)	0.660 x 0.240 x 0.100
On the diffractometer:	
Theta range for data collection	1.80 to 26.51

Limiting indices	-26<=h<=27, -35<=k<=34, -33<=l<=33
Completeness to theta = 30.52 (%)	99.3%
Max. and min. transmission	0.6847 and 0.7457
Reflns collected (not incl. absences)	144918
No of unique reflns, R(int) for equivs	31458, 0.0343
No. of ‘observed’ reflns ($I > 2\sigma_I$)	29643
Refinement:	
Data/restraints/parameters	31458/749/2402
Goodness-of-fit on F^2	1.047
Final R indices (‘obsd’ data)	R_1 = 0.0336, wR_2 = 0.0867
Final R indices (all data)	R_1 = 0.0369, wR_2 = 0.0903
Reflns weighted: 1/w = [a]	[\s^2^(Fo^2^)+(0.0518P)^2^+11.0323P]
Largest diff. peak and hole (e. Å $^{-3}$)	0.958 and -0.332

[a] where P=(Fo^2^+2Fc^2^)/3'

Conformer Sampling and DFT NMR predictions

Solvent free systems. Suitable guess structures were generated for a *fac-fac* and *mer-mer* isomer of the complex and optimized with Gaussian as detailed in the Experimental Section. The optimized structures were then both submitted to the CREST workflow with the following keywords: *--ethr 0.5 --cluster --noreftopo --subrmsd -alpb toluene.*

After completion, the clustered output was screened for duplicates and all unique structures were pre-optimized with TPSSh/cc-pVDZ(-PP) utilizing a smaller grid to reduce computational time (*int=grid=sg1*) and loose convergence criteria. The optimized structures were screened again for duplicates and only unique conformers were fully optimized and evaluated as detailed in the Experimental Section. Conformers are numbered as obtained from the CREST output.

Solvent/Donor coordinated systems. A similar workflow was employed but only starting structures with the solvent/donor in the 1st coordination sphere were considered (*fac-fac* geometry).

Relative Energies of Different Conformers for 3^+ and its solvent/donor complexes

							(t=298.15 K, p=1.0 bar)								
**Conformer**	**Formula**	**Energy(DZ)**	**Energy(TZ)**	**D0**	**NumNegative**	**ZPE**	**EnthalpyCorr**	**EntropyCorr**	**E**	**H**	**G**	**ΔH(rel)**	**ΔG(rel)**	**Conformer**	**τ**
**o-Anth**															
**Cation**															
ONNO_oAnth_ZrBn+_7	C53H45N2O2Zr	-2354.004495	-2354.581111	-0.13164827	0	0.836486	0.88568188	0.131952985	-2354.71	-2353.83	-2353.915485	0.2	0.0	FM	0.38
ONNO_oAnth_ZrBn+_2	C53H45N2O2Zr	-2354.003729	-2354.579791	-0.1335333	0	0.836855	0.885894511	0.131039562	-2354.71	-2353.83	-2353.915226	0.0	0.2	FF	0.4
ONNO_oAnth_ZrBn+_C2x_3	C53H45N2O2Zr	-2353.999621	-2354.581606	-0.12853134	0	0.835491	0.885185131	0.134586989	-2354.71	-2353.82	-2353.915126	1.6	0.2	MM	0.26
ONNO_oAnth_ZrBn+_1	C53H45N2O2Zr	-2354.003864	-2354.580291	-0.1322386	0	0.836941	0.88600761	0.131441427	-2354.71	-2353.83	-2353.914588	0.6	0.6	FF	0.57
ONNO_oAnth_ZrBn+_C2x_5	C53H45N2O2Zr	-2353.999158	-2354.576607	-0.13137979	0	0.836289	0.885489887	0.131621827	-2354.71	-2353.82	-2353.910684	3.1	3.0	FM	0.09
ONNO_oAnth_ZrBn+_C2x_6	C53H45N2O2Zr	-2353.995872	-2354.579562	-0.1256974	0	0.835615	0.885271347	0.134656655	-2354.71	-2353.82	-2353.910208	4.7	3.3	MM	0.06
**chlorobenzene**															
ONNO_oAnth_ZrBn+_chlorobenzene_1	C59H50ClN2O2Zr	-3045.927112	-3046.566746	-0.16182466	0	0.929262	0.985665365	0.148275232	-3046.73	-3045.74	-3045.84225	0.0	0.0		
ONNO_oAnth_ZrBn+_chlorobenzene_6	C59H50ClN2O2Zr	-3045.92171	-3046.5609	-0.1631443	0	0.929698	0.985915487	0.147475992	-3046.72	-3045.74	-3045.836938	3.0	3.3		
ONNO_oAnth_ZrBn+_chlorobenzene_5	C59H50ClN2O2Zr	-3045.916911	-3046.557249	-0.16228447	0	0.929283	0.985714546	0.148155169	-3046.72	-3045.73	-3045.833083	5.7	5.8		
ONNO_oAnth_ZrBn+_chlorobenzene_4	C59H50ClN2O2Zr	-3045.915086	-3046.555173	-0.16071187	0	0.929102	0.985609247	0.149578599	-3046.72	-3045.73	-3045.830493	7.9	7.4		
**toluene**															
ONNO_oAnth_ZrBn+_toluene_10	C60H53N2O2Zr	-2625.625557	-2626.259085	-0.16706271	0	0.966442	1.023251944	0.148049814	-2626.43	-2625.4	-2625.502089	0.0	0.0		
ONNO_oAnth_ZrBn+_toluene_5	C60H53N2O2Zr	-2625.626242	-2626.259865	-0.16575944	0	0.966234	1.023133192	0.148699782	-2626.43	-2625.4	-2625.50212	0.3	0.0		
ONNO_oAnth_ZrBn+_toluene_4	C60H53N2O2Zr	-2625.621008	-2626.261722	-0.15792092	0	0.965065	1.022666785	0.153950402	-2626.42	-2625.4	-2625.500123	3.7	1.3		
ONNO_oAnth_ZrBn+_toluene_1	C60H53N2O2Zr	-2625.615348	-2626.252932	-0.15959392	0	0.964725	1.022120322	0.152186807	-2626.41	-2625.39	-2625.492371	7.8	6.1		
**THF**															
ONNO_ZrBn_pMe_oAnth_Bn+_THF_1	C57H53N2O3Zr	-2586.531462	-2587.161673	-0.16339923	0	0.955594	1.010849679	0.144665591	-2587.33	-2586.31	-2586.411148	0.0	0.0		
ONNO_ZrBn_pMe_oAnth_Bn+_THF_9	C57H53N2O3Zr	-2586.527684	-2587.158136	-0.16371428	0	0.955481	1.010674373	0.144149485	-2587.32	-2586.31	-2586.407757	1.9	2.1		
ONNO_ZrBn_pMe_oAnth_Bn+_THF_6	C57H53N2O3Zr	-2586.526195	-2587.156255	-0.16464099	0	0.955875	1.011030688	0.144197483	-2587.32	-2586.31	-2586.406478	2.7	2.9		
ONNO_ZrBn_pMe_oAnth_Bn+_THF_2	C57H53N2O3Zr	-2586.52586	-2587.156455	-0.16402038	0	0.955425	1.010813024	0.144788417	-2587.32	-2586.31	-2586.406671	2.9	2.8		
ONNO_ZrBn_pMe_oAnth_Bn+_THF_7	C57H53N2O3Zr	-2586.52507	-2587.155108	-0.16585497	0	0.955903	1.010994787	0.143238715	-2587.32	-2586.31	-2586.405938	2.7	3.3		
ONNO_ZrBn_pMe_oAnth_Bn+_THF_14	C57H53N2O3Zr	-2586.522481	-2587.152641	-0.16413406	0	0.955567	1.010746242	0.144202703	-2587.32	-2586.31	-2586.402645	5.1	5.3		
ONNO_ZrBn_pMe_oAnth_Bn+_THF_21	C57H53N2O3Zr	-2586.522033	-2587.155758	-0.1538138	0	0.953924	1.009780092	0.148492186	-2587.31	-2586.3	-2586.399281	9.1	7.4		
ONNO_ZrBn_pMe_oAnth_Bn+_THF_15	C57H53N2O3Zr	-2586.517248	-2587.147733	-0.16268687	0	0.955607	1.010835846	0.144808481	-2587.31	-2586.3	-2586.396606	9.2	9.1		
ONNO_ZrBn_pMe_oAnth_Bn+_THF_20	C57H53N2O3Zr	-2586.515969	-2587.149063	-0.15566961	0	0.95422	1.009975274	0.14792549	-2587.3	-2586.29	-2586.393868	12.2	10.8		
**3,5-Lutidine**															
ONNO_ZrBn_pMe_oAnth_Bn+_35Lut_1	C60H54N3O2Zr	-2681.028345	-2681.675173	-0.16867945	0	0.982182	1.040713466	0.152615827	-2681.84	-2680.8	-2680.905392	0.0	0.0		
ONNO_ZrBn_pMe_oAnth_Bn+_35Lut_2	C60H54N3O2Zr	-2681.024763	-2681.671194	-0.17059931	0	0.982689	1.040938568	0.150696337	-2681.84	-2680.8	-2680.901822	1.4	2.2		
ONNO_ZrBn_pMe_oAnth_Bn+_35Lut_3	C60H54N3O2Zr	-2681.016886	-2681.663892	-0.16958808	0	0.982394	1.040835058	0.151986687	-2681.83	-2680.79	-2680.894476	6.6	6.8		

Relative Energies of Different Conformers for 4^+ and its solvent/donor complexes

							(t=298.15 K, p=1.0 bar)								
Conformer	Formula	Energy(DZ)	Energy(TZ)	D0	NumNegative	ZPE	EnthalpyCorr	EntropyCorr	E	H	G	ΔH(rel)	ΔG(rel)	Conformer	τ
					o-tBu										
					Cation										
ONNO_o_tBu_ZrBn+_C2x_1	C33H45N2O2Zr	-1591.697554	-1592.126817	-0.10436678	0	0.711606	0.752037781	0.110563707	-1592.23	-1591.48	-1591.553224	0.0	0.0	MM	0.28
ONNO_o_tBu_ZrBn+_C2x_5	C33H45N2O2Zr	-1591.695421	-1592.124836	-0.10545563	0	0.71205	0.752248986	0.110059906	-1592.23	-1591.48	-1591.551783	0.7	0.9	FM	0.7
ONNO_o_tBu_ZrBn+_1	C33H45N2O2Zr	-1591.697615	-1592.123089	-0.10619991	0	0.712474	0.752396393	0.108653674	-1592.23	-1591.48	-1591.54969	1.4	2.2	FM	0.51
ONNO_o_tBu_ZrBn+_C2x_9	C33H45N2O2Zr	-1591.695436	-1592.121401	-0.10653361	0	0.712306	0.752298694	0.10863089	-1592.23	-1591.48	-1591.548418	2.2	3.0	FM	0.63
ONNO_o_tBu_ZrBn+_C2x_17	C33H45N2O2Zr	-1591.689195	-1592.120913	-0.10081022	0	0.710939	0.751620297	0.112101464	-1592.22	-1591.47	-1591.545211	5.7	5.0	MM	0.17
ONNO_o_tBu_ZrBn+_6	C33H45N2O2Zr	-1591.690515	-1592.117163	-0.10911348	0	0.712989	0.752588066	0.106429931	-1592.23	-1591.47	-1591.544996	3.4	5.2	FF	0.42
					chlorobenzene										
ONNO_otBu_ZrBn+_chlorobenzene_1	C39H50ClN2O2Zr	-2283.610631	-2284.10174	-0.13268614	0	0.805056	0.852177307	0.125436869	-2284.23	-2283.38	-2283.466292	0.0	0.0		
ONNO_otBu_ZrBn+_chlorobenzene_25	C39H50ClN2O2Zr	-2283.60902	-2284.099577	-0.13377311	0	0.805306	0.852366788	0.125069971	-2284.23	-2283.38	-2283.464781	0.8	0.9		
ONNO_otBu_ZrBn+_chlorobenzene_14	C39H50ClN2O2Zr	-2283.608034	-2284.09808	-0.13504282	0	0.805873	0.852733875	0.124151436	-2284.23	-2283.38	-2283.46357	1.2	1.7		
ONNO_otBu_ZrBn+_chlorobenzene_6	C39H50ClN2O2Zr	-2283.608868	-2284.100234	-0.13116038	0	0.805158	0.852286102	0.124842348	-2284.23	-2283.38	-2283.462752	2.0	2.2		
					toluene										
ONNO_otBu_ZrBn+_toluene_4	C40H53N2O2Zr	-1863.306863	-1863.799161	-0.13018893	0	0.840949	0.88919458	0.129947454	-1863.93	-1863.04	-1863.12722	0.6	0.0		
ONNO_otBu_ZrBn+_toluene_2	C40H53N2O2Zr	-1863.307145	-1863.798902	-0.13066059	0	0.841111	0.889285912	0.129420945	-1863.93	-1863.04	-1863.126988	0.5	0.1		
ONNO_otBu_ZrBn+_toluene_1	C40H53N2O2Zr	-1863.30753	-1863.794014	-0.13725235	0	0.842683	0.890187168	0.125207395	-1863.93	-1863.04	-1863.124969	0.0	1.4		
					THF										
ONNO_ZrBn_otBu_Bn+_THF_2	C37H53N2O3Zr	-1824.212609	-1824.695169	-0.13504218	0	0.832311	0.877990038	0.120028569	-1824.83	-1823.95	-1824.03264	0.0	0.0		
ONNO_ZrBn_otBu_Bn+_THF_3	C37H53N2O3Zr	-1824.212037	-1824.694563	-0.13461305	0	0.83226	0.878012853	0.120858514	-1824.83	-1823.95	-1824.032139	0.7	0.3		
ONNO_ZrBn_otBu_Bn+_THF_8	C37H53N2O3Zr	-1824.209148	-1824.691625	-0.13630088	0	0.831802	0.877484498	0.119462237	-1824.83	-1823.95	-1824.030481	1.1	1.4		
ONNO_ZrBn_otBu_Bn+_THF_4	C37H53N2O3Zr	-1824.208428	-1824.690916	-0.13586712	0	0.831699	0.877474885	0.119927836	-1824.83	-1823.95	-1824.02966	1.8	1.9		
ONNO_ZrBn_otBu_Bn+_THF_1	C37H53N2O3Zr	-1824.207615	-1824.690366	-0.13580424	0	0.831837	0.877711238	0.120395661	-1824.83	-1823.95	-1824.029124	2.4	2.2		
ONNO_ZrBn_otBu_Bn+_THF_14	C37H53N2O3Zr	-1824.207958	-1824.690319	-0.13549034	0	0.832299	0.878001187	0.120212618	-1824.83	-1823.95	-1824.028351	2.8	2.7		
ONNO_ZrBn_otBu_Bn+_THF_15	C37H53N2O3Zr	-1824.206982	-1824.690066	-0.13421079	0	0.831803	0.877686755	0.121175807	-1824.82	-1823.95	-1824.027778	3.5	3.1		
ONNO_ZrBn_otBu_Bn+_THF_9	C37H53N2O3Zr	-1824.206471	-1824.689222	-0.13439083	0	0.832356	0.878161405	0.121002709	-1824.82	-1823.95	-1824.026523	4.2	3.8		
ONNO_ZrBn_otBu_Bn+_THF_10	C37H53N2O3Zr	-1824.201962	-1824.684894	-0.13582062	0	0.83196	0.877807337	0.120405226	-1824.82	-1823.94	-1824.023579	5.8	5.7		
					3,5-Lutidine										
ONNO_ZrBn_otBu_Bn+_35Lut_1	C40H54N3O2Zr	-1918.710947	-1919.209585	-0.14020729	0	0.85882	0.907795866	0.12890813	-1919.35	-1918.44	-1918.528365	0.0	0.0		
ONNO_ZrBn_otBu_Bn+_35Lut_2	C40H54N3O2Zr	-1918.707898	-1919.206306	-0.14076902	0	0.858972	0.907830789	0.127713287	-1919.35	-1918.44	-1918.524812	1.7	2.2		
ONNO_ZrBn_otBu_Bn+_35Lut_4	C40H54N3O2Zr	-1918.707903	-1919.20666	-0.13913263	0	0.858326	0.907327772	0.127859286	-1919.35	-1918.44	-1918.52413	2.2	2.7		
ONNO_ZrBn_otBu_Bn+_35Lut_3	C40H54N3O2Zr	-1918.701817	-1919.20057	-0.14001923	0	0.858626	0.907667833	0.128055086	-1919.34	-1918.43	-1918.518719	5.7	6.1		

NMR predictions for 3^+ and its solvent/donor complexes

					Solvent = Chlorobenzene						Solvent = Toluene					
Name()	ΔH(rel)	ΔG(rel)	Conformer		N-shifts			C-Shifts			N-shifts			C-Shifts		
								o-Anth								
				τ	N1	N2	Δ	NMe1	NMe2	DFT Δ	N1	N2	Δ	NMe1	NMe2	Δ
								Cation								
**ONNO_oAnth_ZrBn+_7**	**0.2**	**0.0**	**FM**	**0.38**	**82.05**	**79.8**	**2.25**	**48.4**	**42.6**	**5.8**						
**ONNO_oAnth_ZrBn+_2**	**0.0**	**0.2**	**FF**	**0.4**	**79.26**	**71.81**	**7.45**	**46.3**	**46.9**	**0.6**						
**ONNO_oAnth_ZrBn+_C2x_3**	**1.6**	**0.2**	**MM**	**0.26**	**84.82**	**82.06**	**2.76**	**43.1**	**38.8**	**4.3**						
**ONNO_oAnth_ZrBn+_1**	**0.6**	**0.6**	**FF**	**0.57**	**82.22**	**77.14**	**5.08**	**41.8**	**52.4**	**10.6**						
**ONNO_oAnth_ZrBn+_C2x_5**	**3.1**	**3.0**	**FM**	**0.09**	**83.93**	**72.62**	**11.31**	**44.4**	**41.4**	**3**						
**ONNO_oAnth_ZrBn+_C2x_6**	**4.7**	**3.3**	**MM**	**0.06**	**81.61**	**79.69**	**1.92**	**44.3**	**39**	**5.3**						
								chlorobenzene								
**ONNO_oAnth_ZrBn+_chlorobenzene_1**	**0.0**	**0.0**			**82.45**	**78.56**	**3.89**	**48.7**	**46.4**	**2.3**						
ONNO_oAnth_ZrBn+_chlorobenzene_6	3.0	3.3														
ONNO_oAnth_ZrBn+_chlorobenzene_5	5.7	5.8														
ONNO_oAnth_ZrBn+_chlorobenzene_4	7.9	7.4														
								toluene								
**ONNO_oAnth_ZrBn+_toluene_10**	**0.0**	**0.0**									**83.81**	**78.38**	**5.43**	**49.9**	**43.3**	**6.6**
**ONNO_oAnth_ZrBn+_toluene_5**	0.3	0.0									**83.64**	**78.18**	**5.46**	**49.9**	**46.4**	**3.5**
ONNO_oAnth_ZrBn+_toluene_4	3.7	1.3														
ONNO_oAnth_ZrBn+_toluene_1	7.8	6.1														
								THF								
**ONNO_ZrBn_pMe_oAnth_Bn+_THF_1**	**0.0**	**0.0**									**79.17**	**78.51**	**0.66**	**48.5**	**46.5**	**2**
**ONNO_ZrBn_pMe_oAnth_Bn+_THF_9**	**1.9**	**2.1**									**75.85**	**72.46**	**3.39**	**47.9**	**45.9**	**2**
**ONNO_ZrBn_pMe_oAnth_Bn+_THF_6**	**2.7**	**2.9**									**82.63**	**80.48**	**2.15**	**53.6**	**51.1**	**2.5**
**ONNO_ZrBn_pMe_oAnth_Bn+_THF_2**	**2.9**	**2.8**									**76.16**	**72.14**	**4.02**	**48.1**	**46.1**	**2**
ONNO_ZrBn_pMe_oAnth_Bn+_THF_7	2.7	3.3														
ONNO_ZrBn_pMe_oAnth_Bn+_THF_14	5.1	5.3														
ONNO_ZrBn_pMe_oAnth_Bn+_THF_21	9.1	7.4														
ONNO_ZrBn_pMe_oAnth_Bn+_THF_15	9.2	9.1														
ONNO_ZrBn_pMe_oAnth_Bn+_THF_20	12.2	10.8														
								3,5-Lutidine								
ONNO_ZrBn_pMe_oAnth_Bn+_35Lut_1	**0.0**	**0.0**									**77.13**	**76.89**	**0.24**	**48.3**	**46.4**	**1.9**
ONNO_ZrBn_pMe_oAnth_Bn+_35Lut_2	**1.4**	**2.2**									**79.9**	**80.98**	**1.08**	**54**	**50.8**	**3.2**
ONNO_ZrBn_pMe_oAnth_Bn+_35Lut_3	6.6	6.8														

NMR predictions for 4^+ and its solvent/donor complexes

					Solvent = Chlorobenzene						Solvent = Toluene					
Name()	ΔH(rel)	ΔG(rel)	Conformer		N-shifts			C-Shifts			N-shifts			C-Shifts		
o-tBu																
				τ	N1	N2	Δ	NMe1	NMe2	DFT Δ	N1	N2	Δ	NMe1	NMe2	Δ
Cation																
ONNO_o_tBu_ZrBn+_C2x_1	0.0	0.0	MM	0.28	85.75	82.18	3.57	49.4	42.8	6.6						
ONNO_o_tBu_ZrBn+_C2x_5	0.7	0.9	FM	0.7	88.42	81.91	6.51	47.2	39.2	8						
ONNO_o_tBu_ZrBn+_1	1.4	2.2	FM	0.51	84.81	81.98	2.83	49.4	42.8	6.6						
ONNO_o_tBu_ZrBn+_C2x_9	2.2	3.0	FM	0.63	86.03	74.3	11.73	45.5	41	4.5						
ONNO_o_tBu_ZrBn+_C2x_17	5.7	5.0	MM	0.17	85.63	84.02	1.61	39.1	44.3	5.2						
ONNO_o_tBu_ZrBn+_6	3.4	5.2	FF	0.42	80.73	72.41	8.32	48.2	48.3	0.1						
chlorobenzene																
ONNO_otBu_ZrBn+_chlorobenzene_1	0.0	0.0			77.61	75.43	2.18	49.1	48.2	0.9						
ONNO_otBu_ZrBn+_chlorobenzene_25	0.8	0.9			83.47	81.11	2.36	54.1	51.2	2.9						
ONNO_otBu_ZrBn+_chlorobenzene_14	1.2	1.7			87.05	84.34	2.71	48.2	46.7	1.5						
ONNO_otBu_ZrBn+_chlorobenzene_6	2.0	2.2			77.93	77.63	0.3	48.2	48.9	0.7						
toluene																
ONNO_otBu_ZrBn+_toluene_4	0.6	0.0									80.27	73	7.27	47.8	48.5	0.7
ONNO_otBu_ZrBn+_toluene_2	0.5	0.1									80.26	73	7.26	47.8	48.6	0.8
ONNO_otBu_ZrBn+_toluene_1	0.0	1.4									84.88	81.26	3.62	49	48.1	0.9
THF																
ONNO_ZrBn_otBu_Bn+_THF_2	0.0	0.0									81.14	80.6	0.54	48.6	48.1	0.5
ONNO_ZrBn_otBu_Bn+_THF_3	0.7	0.3									80.74	80.97	0.23	48.4	48.4	0
ONNO_ZrBn_otBu_Bn+_THF_8	1.1	1.4									76.84	73.9	2.94	49.1	47.4	1.7
ONNO_ZrBn_otBu_Bn+_THF_4	1.8	1.9									76.81	74.05	2.76	49.3	47.5	1.8
ONNO_ZrBn_otBu_Bn+_THF_1	2.4	2.2														
ONNO_ZrBn_otBu_Bn+_THF_14	2.8	2.7														
ONNO_ZrBn_otBu_Bn+_THF_15	3.5	3.1														
ONNO_ZrBn_otBu_Bn+_THF_9	4.2	3.8														
ONNO_ZrBn_otBu_Bn+_THF_10	5.8	5.7														
3,5-Lutidine																
ONNO_ZrBn_otBu_Bn+_35Lut_1	0.0	0.0									79.58	79.7	0.12	48.5	48	0.5
ONNO_ZrBn_otBu_Bn+_35Lut_2	1.7	2.2									82.5	84.8	2.3	53.2	51.7	1.5
ONNO_ZrBn_otBu_Bn+_35Lut_4	2.2	2.7														
ONNO_ZrBn_otBu_Bn+_35Lut_3	5.7	6.1														

Additional Polymerization Details

Table S6. Detailed polymerization results. T_p = 60°C, $p_{propene}$ = 6.6 bar.

# ID	Cat (nmol)	t_p (s)	Yield (mg)	R_p*	M_n, kDa	M_w, kDa	PDI	σ	$σ_{av}$
**1**	800	6875	59	0.04	3.5	5.9	1.7	0.9979	0.997_5
	800	3415	56	0.07	2.7	4.8	1.8	0.9973	
**2**	25	339	72	30.6	1.3	1.8	1.5	0.80	0.80
	25	879	73	12.0	1.3	1.9	1.4	0.80	
**3**	50	277	85	22.1	1.5	2.3	1.5	(a)	(a)
	50	470	73	11.2	1.5	2.3	1.5	(a)	(a)
**4**	2400	3155	27	0.01	0.7	0.8	1.2	0.93	0.93
	2400	3541	28	0.01	0.7	0.8	1.2	0.93	
**5**	1200	939	38	0.12	4.6	8.1	1.8	0.986	0.986
	1200	669	38	0.17	5.5	9.2	1.7	0.986	
**6**	800	2546	40	0.07	3.0	4.8	1.6	0.972	0.974
	800	2145	34	0.07	3.0	5.0	1.6	0.975	
**7**	50	231	62	19.3	67	141	2.1	≥0.9998	>0.9998
	50	223	63	20.3	70	146	2.1	≥0.9998	

(a) Chain-end controlled syndiotactic polypropylene, * in kg $mmol^{-1}$ h^{-1}

References

(1) Perrin, C. L.; Dwyer, T. J. Application of Two-Dimensional NMR to Kinetics of Chemical Exchange. *Chem. Rev.* **1990**, *90* (6), 935–967.

(2) https://mestrelab.com/software/freeware/.

(3) Bain, A. D. Chemical Exchange in NMR. *Prog. Nucl. Magn. Reson. Spectrosc.* **2003**, *43* (3–4), 63–103.

(4) Busico, V.; Cipullo, R.; Friederichs, N.; Ronca, S.; Talarico, G.; Togrou, M.; Wang, B. Block Copolymers of Highly Isotactic Polypropylene via Controlled Ziegler−Natta Polymerization, *Macromolecules* **2004**, *37*, 8201-8203.

(5) Ye, X.; Atienza, C.C.H.; Holtcamp, M.W.; Sanders, D.F.; Day, G.S.; Titone, M.E.; Cano, D.A.; Bedoya, M.S. *Int. pat. appl. WO2017058388, 2017*

(6) Cipullo, R.; Busico, V.; Fraldi, N.; Pellecchia, R.; Talarico, G. *Macromolecules* **2009**, *42*, 3869-3872.

(7) Antinucci, G.; Dereli, B.; Vittoria, A.; Budzelaar, P.H.M.; Cipullo, R.; Goryunov, G.P.; Kulyabin, P.S.; Uborsky, D.V.; Cavallo, L.; Ehm, C.; Voskoboynikov, A.Z.; Busico, V. Selection of Low-Dimensional 3-D Geometric Descriptors for Accurate Enantioselectivity Prediction *ACS Catal*. **2022**, *12*, 6934-6945.

(8) Gisch, N.; Balzarini, J.; Meier, C. Enzymatically Activated *cyclo*Sal-d4T-monophosphates: The Third Generation of *cyclo*Sal-Pronucleotides *Journal of Medicinal Chemistry* **2007**, *50*, 1658-1667.

(9) Chandran, R.; Madhaiyan, K.; Sridhar, R.; Mahalakshmi, S. Synthesis and Characterization of Anthracene Functionalized Novel Fluorescent Amino Acids: Interactions of the

Fluorescent Amino Acids with Metal Ions *Current Organic Synthesis* **2016**, *13*, 646-650.

(10) Zhu, J.-B.; Chen, E. Y.-X.; Catalyst-Sidearm-Induced Stereoselectivity Switching in Polymerization of a Racemic Lactone for Stereocomplexed Crystalline Polymer with a Circular Life Cycle *Angew. Chem. Int. Ed.* **2019**, *58*, 1178–1182.
